# Identification of *Leishmania donovani* Topoisomerase 1 inhibitors *via* intuitive scaffold hopping and bioisosteric modification of known Top 1 inhibitors

**DOI:** 10.1038/srep26603

**Published:** 2016-05-25

**Authors:** Rajinikanth Mamidala, Papiya Majumdar, Kunal Kumar Jha, Chandramohan Bathula, Rahul Agarwal, M. Thirumala Chary, H. K. Mazumdar, Parthapratim Munshi, Subhabrata Sen

**Affiliations:** 1 Department of Chemistry, Jawaharlal Nehru Technological University, Kukatpally, Hyderabad 500085, Telangana, India; 2 GVK Bioscience, 28A IDA Nacharam, Hyderabad, Telengana, India; 3 Institute of Chemical Biology, 4 Raja S.C. Mullick Road, Kolkata 700032, West Bengal, India; 4 Department of Chemistry, School of Natural Sciences, Shiv Nadar University, Chithera, Dadri, Gautam Buddha Nagar 201314, Uttar Pradesh, India; 5 Department of Life Sciences, School of Natural Sciences, Shiv Nadar University, Chithera, Dadri, Gautam Buddha Nagar 201314, Uttar Pradesh, India

## Abstract

A library of arylidenefuropyridinediones was discovered as potent inhibitors of *Leishmania donovani* Topoisomerase 1 (LdTop1) where the active molecules displayed considerable inhibition with single digit micromolar EC_50_ values. This molecular library was designed *via* intuitive scaffold hopping and bioisosteric modification of known topoisomerase 1 inhibitors such as camptothecin, edotecarin and etc. The design was rationalized by molecular docking analysis of the compound prototype with human topoisomerase 1 (HTop1) and *Leishmania donovani* topoisomerase 1(LdTop1). The most active compound **4** displayed no cytotoxicity against normal mammalian COS7 cell line (~100 fold less inhibition at the EC_50_). Similar to camptothecin, **4** interacted with free LdTop1 as observed in the preincubation DNA relaxation inhibition experiment. It also displayed anti-protozoal activity against *Leishmania donovani* promastigote. Crystal structure investigation of **4** and its molecular modelling with LdTop1 revealed putative binding sites in the enzyme that could be harnessed to generate molecules with better potency.

Human visceral leishmania, caused by *Leishmania Donovani* is one of the gruesome diseases causing fatalities to nearly 30% of global population^1^. It primarily affects the spleen and liver^2^. Presently, treatment of leishmaniasis relies on expensive chemotherapeutic agents such as pentamidine, amphotericin B and teratogenic miltefosine^3^,^4^,^5^. Hence impromptu drug therapy for Leishmania infection is indeed desirable and need of the hour. In a bid to discover new molecular entities against Leishmania recent research has been focused on DNA topoisomerases, a class of enzymes that modulates DNA replication, transcription and recombination^6^,^7^,^8^,^9^,^10^. Broadly, topoisomerases are classified as type 1 (Top1) and type 2 (Top 2). The classification depends on their ability to cleave the single or double strands of DNA^11^.

Scaffold hopping is a strategy to design architecturally novel compounds by remolding the central core of known active molecules^12^. Resulting compounds possess chemically different core structure and yet exhibit improved modulation of the same biological target. Scaffold hopping is the focus of attention of traditional and modern day drug discovery and requires intuitive and computational techniques for its execution. For example novel non-benzodiazepine GABA-receptor ligands such as Zopiclone, Zolpidem and Zaleplon were discovered way back in 1950, by scaffold hopping of benzodiazepine core^13^. Another interesting set of examples are of dopamine agonists such as Fenoldopam or Quinpirole which were discovered by scaffold hopping of natural ligands^14^,^15^. Antiinflammatory COX inhibitors such as Lumiracoxib, Sulindac, Celecoxib and rofecoxib with diverse scaffolds were obtained from scaffold hopping of indomethacin^16^,^17^.

Bioisosteric modification is a med-chem strategy for rational design of new drugs *via* replacement of chemical functionalities of a bioactive molecule with moieties that possess similar physical or chemical properties to evoke better biological responses. Many drug attributes such as improvement of selectivity, metabolic stability, reduction of side effects and etc. can be modulated with appropriate bioisosterism. For example fluorine replacing hydrogen as a bioistere has a widespread application in drug discovery^18^. Other than providing metabolic stability it also influences lipophilicity of the resulting molecule. Carboxylic acid bioisosteres such as oxadiazoles, oxazole, tetrazoles and etc. provide enhancement of potency and increase of lipophilicity^19^. In another example biosiosteric replacement of amide with trifluoroethylamine in Cathepsin K inhibitors provided improvement in potency, selectivity and metabolic stability^20^.

Herein we report discovery of a novel, selective noncamptothecin inhibitors of LdTop1, based on arylidenefuropyridinedione scaffold *via* intuitive scaffold hopping and bioisosteric modification of known Top1 inhibitors such as Camptothecin, Edotecarin, Diflomotecan and Rosettacin. The design was rationalized by molecular modeling of the new scaffold with both Ld and HTop1. A library was synthesized based on the designed scaffold and enzymatic profiling of the library revealed that the compounds inhibit LdTop1 in a similar manner as Camptothecin. Hirschfield Surface Analysis of the most active compound **4** and its molecular modelling with LdTop1 revealed potential binding pockets of the enzyme.

## Results and Discussion

### Design and molecular docking

**Figure 1:**
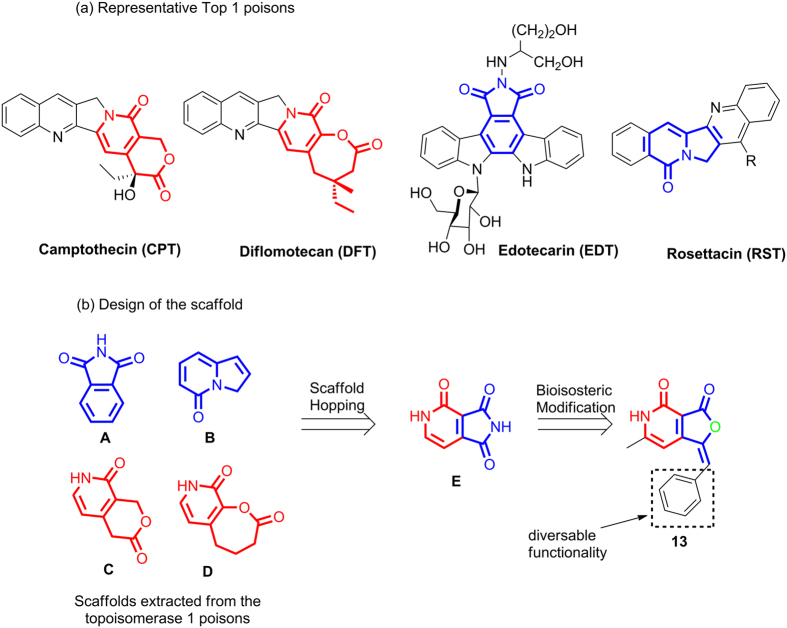
(**a**) Representative Top1 inhibitors; (**b**) Design of the scaffold.

To begin with the design of novel inhibitors of topoisomerase 1 (human or *Leishmania donovani*), we scrutinized the structures of existing topoisomerase inhibitors such as camptothecin derivatives, E-ring modified camptothecin derivatives and non-camptothecin molecules (Fig. 1a)^21^,^22^,^23^.

Careful analysis of the structures in Fig. 1a revealed the presence of four common motifs, the phthalimide (A), indolizinone (B), pyridopyranones (C), and pyridooxepinones (D) (Fig. 1b) within the structural framework of the molecules. Next, these four motifs (**A**–**D**) were utilized to generate **E** by scaffold hopping (Fig. 1b). Subsequent bioisosteric modification of E afforded furopyridinedione derivative **13** as the library scaffold. The aryl moiety provides option for further diversification if required at a later stage (Fig. 1b).

**Figure 2:**
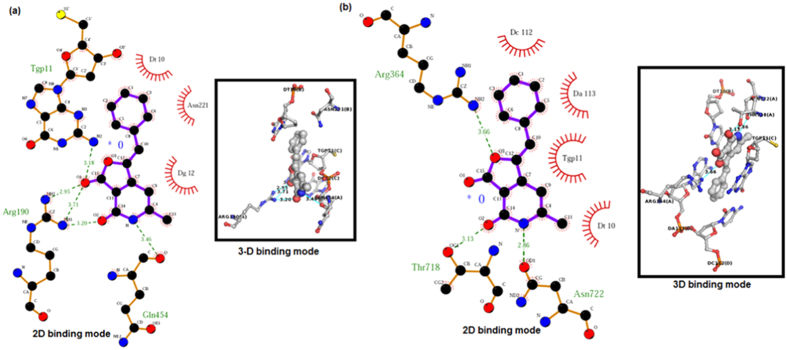
(**a**) 2D and 3D binding mode of **13** with LdTop1; (**b**) 2D and 3D binding mode of **13** with HTop1.

To rationalize our design, **13** was docked with LdTop1. The experiment revealed several binding interactions such as intercalation at the DNA cleavage site, five hydrogen bonding with residues Arg190, Gln454 and Tgp11 and nonbonding interactions with Asn221, Dt10 and Dg12 (Fig. 2a) (refer *SI* for details of the docking experiment involving protein preparation). With HTop1 there were lesser H-bonding interactions (3 *vs* 5) (Fig. 2b). Consequently **13**-LdTop1 complex (−8.07 kcal/mole) was ~0.4 kcal/mole more stable than **13**-HTop1 complex (−7.70 kcal/mole). This was comparable to the binding interactions of camptothecin and edotecarin with both LdTop1 and HTop1 (refer *SI*).

Next, in a bid to understand the physicochemical indications rudimentary to the design we computed few relevant medicinal chemistry descriptors of **13** along with CPT, DFT, EDT and RST (Table 1). From the information it was revealed that **13**, CPT, RST and DFT adhere to the Lipinski rules and that the physicochemical properties of **13** resides well within the range of these but EDT (Table 1). With very high molecular weight (MW), hydrogen bond donor and acceptor (HBD and HBA) ability, total polar surface area (TPSA) and rotatable bond, EDT stands apart from the rest. Interestingly compound **13** has the lowest Log P (Table 1) which indicated better membrane absorption and binding ability to the enzyme but poor aqueous solubility among all the molecules. Low log S_w_ of compound **13** (almost at par with CPT [−3.64 *vs* −3.4]) indicated that substituting **13** with polar functionalities may improve the overall solubility. Finally **13** had the best oral bioavailability amongst all (Table 1).

### Chemistry

Based on these docking results, we decided to generate a library based on **13** using combinatorial synthesis. Accordingly Knoevenagel condensation of furopyridinedione with appropriate aromatic and heteroaromatic aldehydes was explored with different solvents such as ethanol, methanol, isopropanol, *n*-butanol, acetonitrile and tetrahydrofuran. The reaction in ethanol happened to be most efficient where the product precipitates at the end of the reaction and could be isolated and purified by filtration and washing with ethanol without the need for chromatographic separation.

**Figure 3:**
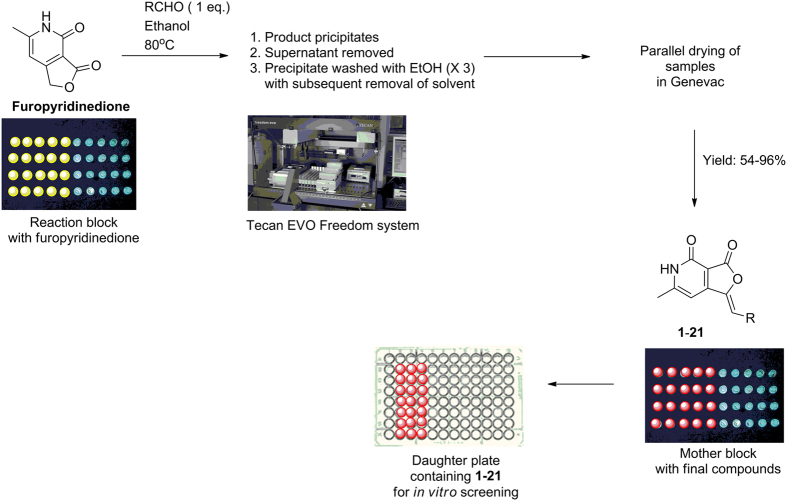
Protocol for the combinatorial synthesis of furopyridinedione derivatives **1–22**. The products precipitate from the reaction mixtures and are isolated by decantation of the supernatant solvent followed by washing.

With the optimized protocol in place, next the Knovenagel reaction was conducted in parallel in custom made metallic reaction blocks containing pre-tared reaction vials with 1 mM solution of furopyridinedione at 80 °C (Fig. 3). As expected the product precipitated on completion of the reaction. The supernatant ethanol was removed and the precipitate was washed thrice with ethanol and the solvent was subsequently removed. The whole operation was executed in parallel using a Tecan Evo Freedom liquid handling system containing robotics arms. The vials were dried in a genevac and weighed to determine the amount. An assay plate was generated with the samples at 50 μM concentration in dimethyl sulfoxide (DMSO), for *in vitro* screening. The average yield of the compounds ranged from 54–96%. The compounds were characterized by ^1^H and ^13^C nuclear magnetic resonance spectroscopy (NMR) and high resolution mass spectroscopy (HRMS). The proton NMR unveiled the characteristic amide proton of the pyridone as broad singlet at ~12 ppm. A sharp singlet at ~6.85 ppm attributed to the alkenyl group present in the compounds.

### *In vitro screening* and structure activity relationship (SAR)

To investigate the inhibitory effect of our library compounds on the DNA relaxation activity of the recombinant Human and *Leishmania* topoisomerase 1 (Table 2), assays were performed involving supercoiled plasmid DNA, the enzymes (HTop1 and LdTop1) and our molecules. The enzymes were purified by the standard literature procedure^9^. The inhibitory activities of our molecules against HTop1 and LdTop1 (EC_50_) were compared to that of the standard top1 inhibitor camptothecin and the results are shown in Table 2. Several molecules (**1**, **2**, **3**, **4**, **9**, **14** and **15**) showed considerable activity against LdTop1. However none of the molecules from the library exhibited inhibition against the HTop1, rendering them selective towards LdTop1. This is interesting when compared to the original molecules such as Camptothecin, Diflomotecan, Edotecarin and Rosettacin, which were used to design this scaffold. Camptothecin a Top1 poison has comparable inhibitory effect on both LdTop1 and HTop1. Nevertheless the other three compounds which are clinical candidates against various cancers are selective HTop1 inhibitors. This is noteworthy because our molecules inspired from majorly selective HTop1 inhibitors displayed particular binding to LdTop1 over HTop1 during *in vitro* screening (Table 2), which was predicted during the molecular docking (Fig. 2) exercise.

**Figure 4:**
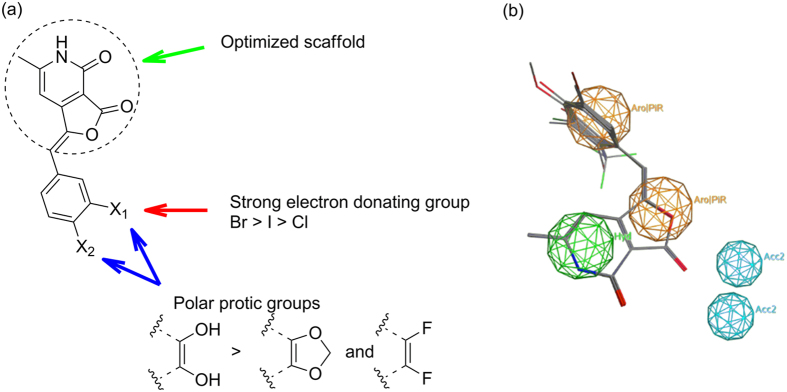
(**a**) Initial structure activity relationship studies. (**b**) Pharmacophore model.

The structure activity relationship study demonstrated the importance of the substituted aromatic moiety towards the LdTop1 inhibitory activity (Fig. 4a). In general the heteroaromatic (**5** and **7**), naphthalene (**11**) and aliphatic (**16**) derivatives, exhibited no inhibitory activity at all. Among the monosubstituted aromatic analogs the electron withdrawing *o*-trifluoromethyl, **3** (EC_50_ ~5 μM) and electron donating *m*-bromo, **9** (EC_50_ ~7.5 μM) substituents on the ring favors the inhibitory activity. Other *meta*- substituted compounds such as chloro and iodo analogs (**14** and **15**) also exhibited inhibitory activities of 17.42 ± 1.86 and 35.57 ± 2.42 μM, respectively. All the *para* substituted analogs rendered inactive. Among the di-substituted compounds, the 3,4-dihydroxyphenyl derivative **4** exhibited the best potency among all the analogs with an EC_50_ of 4 μM. Interestingly, protecting the dihydroxy functionality as in **6** or installing electron withdrawing groups on the same position as in **8** led to the complete loss of activity. The 2,5-dimethyl analog, **1** was moderately active with an EC_50_ of 23 μM, whereas the 2,3-dimethoxy analog **12** was inactive. The only tri-substituted derivative **2** was decently active with EC_50_ of 10 μM. Apart from the importance of the pyridone moiety, the present study unveils two distinct SAR (Fig. 4) trend: (a) may be polar protic functionalities capable for H-bond formation at C_3_ and C_4_ positions of the aromatic ring is beneficial for the activity of the molecules and (b) strong electron donating functionality at *meta* position of the aromatic ring augments the inhibitory activity.

Additionally we performed pharmacophore analysis of our library. Accordingly pharmacophore queries (3D arrangement of molecular features) were devised from both active and inactive compounds of our library against LdTOP1 such that all or most of active compounds satisfied the queries (Fig. 4b). The analysis was done using pharmacophore elucidator module of MOE^TM^.

All the library compounds were sketched and energy minimized using MOE^TM^. The overlap score of 6.6636 indicated that major active molecules (7) were aligned in the design. The best query on the basis of overlap score (alignment score) divulged 2 aromatic/pi ring centers (Aro|PiR) at the arylidene and furanone moiety, one hydrophobic centroid (Hyd) at the pyridone domain and two hydrogen-bond acceptor projection (Acc2):2) that are strategic and could be harnessed for further diversification (Fig. 4b).

### Studies with compound 4

**Figure 5:**
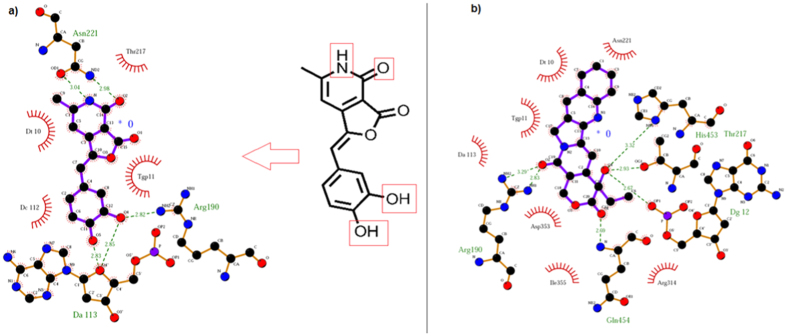
(**a**) Binding pose of **4** in 2D with LdTop1; (**b**) Binding pose of Camptothecin in 2D with LdTop1.

In a bid to justify highest *in vitro* activity of **4**, it was further docked with LdTop1 enzyme (Fig. 5b). The experiment revealed that at the best pose, **4** and LdTOP1-DNA complex intercalates at the DNA cleavage site (Fig. 2, *SI*). The key binding sites in **4** as depicted in Fig. 5a are the pyridine nitrogen, the amide carbonyl and the two aromatic hydroxy functionalities. They form hydrogen bonding interactions with Arg190, Asn221 and DA113 and nonbonding interactions with Thr217, DT10, TGP11, DC112 residues of the LdTop1 enzyme (Fig. 5a). These were comparable to the interactions of camptothecin and LdTop1 (Fig. 5b).

**Figure 6:**
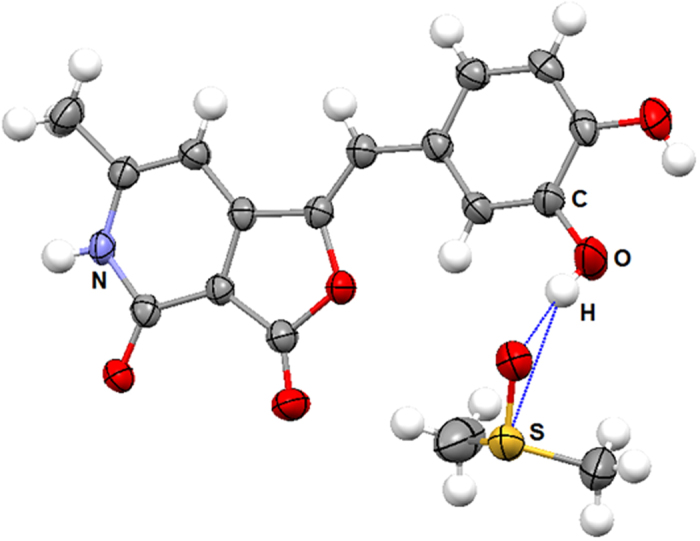
Ellipsoid plot of 4, co-crystallized with DMSO. Ellipsoids of non-H atoms are drawn at 50% probability level. The color codes of different atom types are shown in the figure.

Further to comprehend the interactive nature of **4** a detailed three-dimensional structural investigation was carried out. The X-ray crystal structure determination revealed that compound **4** has crystallized along with dimethyl sulfoxide (DMSO); the solvent used for the crystallization. The molecule is in planar geometry and its *m*-hydroxy group forming strong O-H…O (1.824 Å) and O-H…S (2.900 Å) hydrogen bonds with the Oxygen and Sulfur atoms of DMSO, respectively, as depicted in Fig. 6.

**Figure 7:**
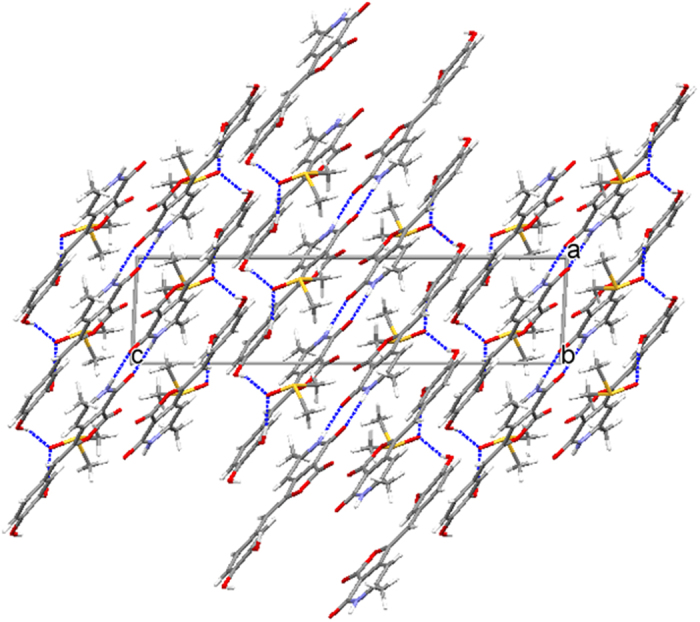
Molecular packing diagram of 4, viewed down the *b*-axis, showing the hydrogen bonding networks in the structure.

The molecular packing diagram in the crystal lattice of **4** as shown in Fig. 7 reveals the presence of dimer motifs forming *via* N-H…O hydrogen bonds (2.054 Å) between the furopyridine moieties. The dimers are noticed at the corner and middle of the unit cell. The O-H…O hydrogen bonds forms between the hydroxyl groups of **4** and the Oxygen atom of DMSO molecules are also evident from the packing diagram.

**Figure 8:**
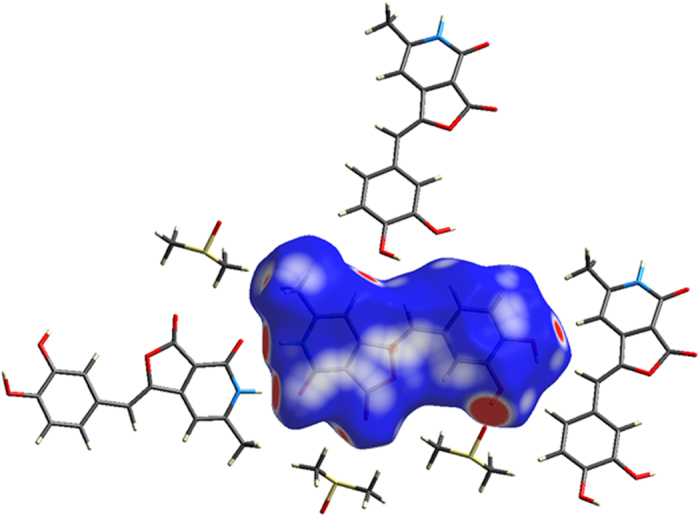
Hirshfeld surface generated on molecule 4 showing the intermolecular contacts with its neighbor molecules.

The possible interactions in **4** were further visualized by performing Hirshfeld surface analysis^24^,^25^,^26^. which highlights the contact points of a molecule with its neighboring molecules in a crystal. The dimer formed *via* strong N-H…O hydrogen bonds and the strong O-H…O bonds formed with DMSO molecule are highlighted as large dark red circular surfaces (Fig. 8). Other weaker interactions (C-H…O) are also highlighted as small red circular surfaces. The interactions such as C-H…*π*, H…H and the non-bonded interactions are highlighted as fainted red surfaces. The hydrogen bonding interactions and the non-bonding interactions as discovered from molecular docking are in correspondence with this qualitative analysis.

**Figure 9:**
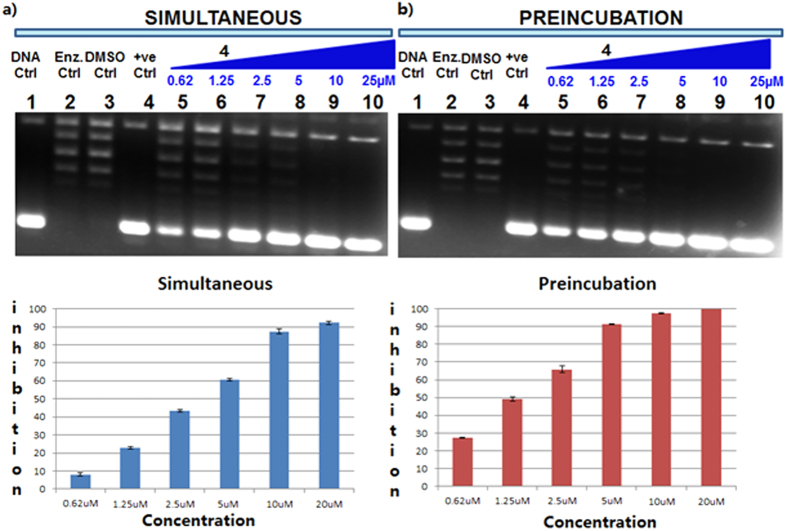
(**a**) Simultaneous assay: Lane 1, 90 fmol of pBS(SK^+^) DNA; Lanes 2 → 4, same as lane 1, but incubated simultaneously with 30 fmol of LdTop1 with reaction buffer, 2% DMSO and camptothecin respectively, for 30 min at 37 °C; lanes 5–10, same as lane 1, but incubated simultaneously with 30 fmol of LdTop1 with the variable concentrations of compound **4** (as indicated) with pBS (SKþ) DNA at 37 °C for 30 min. The corresponding quantitative representation of percentage inhibition of LdTop1 under simultaneous conditions in the presence of indicated compound **4** concentrations in relaxation experiments; (**b**) Preincubation assay: Lane 1, 90 fmol of pBS(SK^+^) DNA; Lanes 2 → 4, same as lane 1, but DNA was added after preincubation with 30 fmol of LdTop1 with reaction buffer, 2% DMSO and with camptothecin, respectively, for 30 min at 37 °C; lanes 5–10, same as lane 1, but preincubation of LdTop1 with the variable concentrations of compound **4** (as indicated) with pBS (SKþ) DNA at 37 °C for 30 min. The corresponding quantitative representation of percentage inhibition of LdTop1 under preincubation conditions in the presence of indicated compound **4** concentrations in relaxation experiments. All the experiments were conducted thrice and representative data from once set of them are expressed as mean ± SD.

To interrogate whether compound **4** interacts with the enzyme, it was preincubated with LdTop1 at different concentration for 5 minutes at 37 °C before the addition of substrate DNA (Fig. 9a). The inhibitory effect of **4** in preincubation condition was further compared with its inhibition when incubated simultaneously with LdTop1 and super coiled DNA in the relaxation experiment (Fig. 9b). Nearly 100% inhibition was observed at 20 μM of **4** under preincubation compared to ~92% inhibition under simultaneous condition. And the EC_50_ values for simultaneous and preincubation assays were 3.77 ± 0.27 and 1.72 ± 0.23 μM respectively. From this results we conclude that the inhibition profile was marginally improved by preincubation of enzyme with **4**.

Next, compound **4** was screened against extracellular promastigotes^27^. The results (depicted in Table 3), revealed that it inhibited the proliferation of *Leishmania donovani* with an IC_50_ of 4.21 ± 0.21 μM. Standard leishmanial drug, miltefosine was used as a positive control and exhibited IC_50_ of ~1 μM. It was subsequently tested for its cytotoxicity against healthy COS7 mammalian cell lines and exhibited toxicity at a concentration nearly 100 times its IC_50_ values (Table 3).

## Conclusion

In this manuscript we reported the design of a series of furopyridinedione derivatives as LdTop1 inhibitors *via* intuitive scaffold hopping and bioisosteric modification of known top1 inhibitors. The design was rationalized by molecular docking of the representative molecule **13** with LdTop1 and HTop1. The compounds were accessed by an efficient parallel synthetic strategy. *In vitro* evaluation of the compounds against LdTop1 and HTop1 unveiled selective LdTop1 inhibition where six compounds exhibited activity with EC_50_ in the range of 1–30 μM. The most active compound **4**, interacted with free LdTop1 as observed in the preincubation DNA relaxation inhibition experiment (Fig. 8b). The active compounds showed minimal toxicity when screened against mammalian COS7 cells. Pharmacophore modelling, X-ray crystallographic investigation of **4** and molecular docking studies of **4**/LdTop1-DNA ternary complex recommended the following structural attributes necessary to attend optimum LdTop1 inhibitory activity for our molecules: (i) functionalized pyridinedione as the central scaffold; (ii) polar protic functionality at the C_3_ and C_4_ and/or (iii) an electron donating substituent at C_3_ position of the benzene ring attached to the pyridinedione moiety.

## Methods

### Chemistry

All reactions were carried out in flame-dried sealed tubes with magnetic stirring. Unless otherwise noted, all experiments were performed under argon atmosphere. All reagents were purchased from Sigma Aldrich, Acros or Alfa Aesar. Solvents were treated with 4 Å molecular sieves or sodium and distilled prior to use. Purifications of reaction products were carried out by column chromatography using Chem Lab silica gel (230–400 mesh). Infrared spectra (IR) were recorded on a Thermoscientific Neoled IS5 FTIR spectrophotometer and are reported as wavelength numbers (cm^−1^). Infrared spectra were recorded by preparing a KBr pellet containing the title compound. ^1^H NMR and ^13^C NMR spectra were recorded with tetramethylsilane (TMS) as internal standard at ambient temperature unless otherwise indicated on a Varian 300/400 and JEOL JNM-ECX500 MHz at 500 MHz for ^1^H NMR and 100 MHz for ^13^C NMR. Chemical shifts are reported in parts per million (ppm) and coupling constants are reported as Hertz (Hz). Splitting patterns are designated as singlet (s), broad singlet (bs), doublet (d), triplet (t). Splitting patterns that could not be interpreted or easily visualized are designated as multiple (m). The Mass Spectrometry analysis was done on the 6540 UHD Accurate-Mass Q-TOF LC/MS system (Agilent Technologies) equipped with Agilent 1290 LC system obtained by the Dept. of Chemistry, School of Natural Sciences, Shiv Nadar University, Uttar Pradesh 203207, India.

### Plasmid relaxation assay

The type I DNA topoisomerase are assayed by decreased mobility of the relaxed isomers of supercoiled pBS (SK^+^) [pBlue-script (SK^+^)] DNA in agarose gel. The relaxation assay was carried out as described previously with LdTop1B and HTop I, diluted in the relaxation buffer (25 mM Tris–HCl, pH 7.5, 5% glycerol, 0.5 mM DTT, 10 mM MgCl_2_, 50 mM KCl, 25 mM EDTA and 150 mg/ml BSA) and supercoiled plasmid pBS (SK^+^) DNA (85–95% were negatively supercoiled, with remainder being nicked circles)^9^,^28^,^29^. The reconstituted enzyme LdTop1B was assayed at 50 mM KCl concentration as described before^30^. For all kinetic studies, the reaction mixtures containing the buffer and DNA were heated to 37 °C before addition of the enzymes. The reactions were rapidly quenched using stop solution and kept on ice. The gels were stained with ethidium bromide (EtBr) (0.5 mg/ml) and the amount of supercoiled monomer DNA band fluorescence were quantified by integration using Gel Doc 2000 under UV illumination (Bio-Rad Quantity One Software), as described previously^30^.

### Purification of recombinant human topoisomerase I (HTop1)

The wild-type HTop1 (91 kDa) was purified from Sf-9 insect cells infected with the recombinant baculovirus (a kind gift from Prof. J. J. Champoux). Approximately, 1 * 10^9^ Sf-9 cells were infected with the recombinant virus, and cells were harvested after 48-h of infection. The cells were lysed and enzyme was purified as described^31^. Briefly, at 48 h post-infection, approximately 3 * 10^9^ Sf-9 cells were harvested by centrifugation for 5 min at 400 * g. The cells were resuspended in 1 liter of ice-cold phosphate-buffered saline and centrifuged for 5 min at 400 * g. The nuclei were washed twice in 80 ml of lysis buffer minus Triton X-100 and resuspended in 40 ml of resuspension buffer (50 mM KCl, 10 mM Tris-Cl, pH 7.5, 2 mM MgCl_2_, 25 mM DTT, 0.4 mg/ml phenylmethylsulfonyl fluoride, 0.12 mg/ml aprotinin). The nuclear extract was stirred for 5 min at ~200 rpm. With continued stirring, 50 ml of polyethylene glycol (PEG) buffer (18% PEG 8000, 1 M NaCl, 10% glycerol) was added dropwise in order to precipitate the DNA. The PEG precipitate was pelleted by centrifugation and the resulting PEG supernatant was dialyzed against 4 liters of potassium phosphate buffer. The dialyzed PEG supernatant passed over a 10-ml bed volume of phosphocellulose (Whatman P11) equilibrated with PPB. The HTop1 flowed through the PS column and then passed over a Mono Q column followed by loaded onto a Mono S column. The Mono S column was eluted with a 25-ml 50–200 mM KPO_4_, pH 7.4, gradient. HTop1 eluted as a single major peak at 150 mM KPO_4_. The peak Mono S fractions were pooled, concentrated with an Amicon ultrafiltration cell, dialyzed into storage buffer (50% glycerol, 10 mM Tris-hydrochloride, pH 7.5, 1 mM DTT, 1 mM EDTA), and stored at −20 °C.

### Purification and reconstitution of two subunits of *Leishmania donovani* Topoisomerase I: The full-length large subunit gene (LdTOP1L) and the small subunit gene (LdTOP1S)

*Escherichia coli* BL21 (DE3) pLysS cells harbouring pET16bLdTOP1L and pET16bLdTOP1S, were separately induced at OD_600_ = 0.6 with 0.5 mM IPTG (isopropyl β-D-thiogalactoside) at 22 °C for 12 has described previously^9^. Cells harvested from 1 litre of culture were separately lysed by lysozyme/sonication, and the proteins were purified through Ni-NTA (Ni^2+^-nitriloacetate-agarose column (Qiagen, Hilden, Germany) followed by a phospho-cellulose column (P11 cellulose; Whatman, Maidstone Kent, UK) as described previously^9^. Finally, the purified proteins LdTOP1L and LdTOP1S were stored at −70 °C. The concentrations of each protein were quantified by using Bradford Reagent (PIERCE, Thermo Fisher Scientific Inc., Rockford, USA). Purified LdTOP1L was mixed with purified LdTOP1S at a molar ratio of 1:1 to a total protein concentration of 0.5 mg/ml in reconstitution buffer [50 mM potassium phosphate, pH 7.5, 0.5 mM DTT, 1 mM EDTA, 0.1 mM PMSF and 10% (v/v) glycerol]. The mixture was dialyzed overnight at 4 °C and dialyzed fractions were used for plasmid relaxation activity.

### MTT Assay

*L. donovani* AG83 promastigotes (3.0 × 10^6^ cells/ml) are incubated with different concentrations of drugs/compounds under study (1, 2, 5, 10 and 20 μM) for 12 h, following which the survival percentage is estimated by MTT assay. Yellow MTT (3-(4,5-dimethylthiazol- 2-yl)-2,5-diphenyltetrazolium bromide, a tetrazole) is reduced to purple formazan in the mitochondria of living cells. The formazan is then solubilized, and the concentration is determined by optical density at 570 nm. Metabolically active cells convert MTT to formazan, thereby generating a quantitative measure of viability and cytotoxicity. Percentage of viable promastigotes in each treatment groups are determined with respect to untreated control cells.

### Docking Studies with Human TOP1

#### Methodology

##### Molecular Modeling

Crystal structure of Human TOP1 (PDB ID: 1T8I) is downloaded from PDB. This crystal structure is having various missing residues and side chains so these were added using structure preparation module of MOE software. Further energy was minimized using MOE energy minimization algorithm using Force Field MMFF94x. 3D protonation of the protein were carried in order to prepare protein for docking. A grid of 60 × 60 × 60 with 0.375 Å were constructed around the protein DNA complex area using Autogrid v 4.2 software. The 2D structure of compounds was built using MOE-builder tool and minimized using MMFF94x force field. Docking analysis were done using Autodock v 4.2 software in which top 10 docked conformation were taken using Genetic Algorithm. Each docking calculation consisted of 25 × 10^6^ energy evaluations with 250 population size. A mutation rate of 0.02 and a crossover rate of 0.8 were used to generate new docking trials. The LdTOP1 structure was also minimized using AMBER force field and displayed 0.00 rmsd value when compared to the structure minimized using MMFF94x force field.

### Docking Studies with LdTOP1

#### Methodology

##### Molecular Modeling

We selected crystal structure of LdTop1 (PDB ID: 2B9S) which is available on protein data bank for docking studies. The crsytal structure is having protein large subunits (27-456 residues) and small subunits (221-262) covalent complex with a 22 bp DNA. The crystal structure had various missing residues and side chains which were added using structure preparation module of MOE software. 3D protonation of the proteins were executed in order to prepare them for docking. Water molecules were removed and hydrogen atoms were added and energy was minimized by MOE energy minimization module using MMFF94x force field.

Further we used the procedure described by Roy *et al.* in which the 22 DNA double strand is substituted with 22 DNA double strand present in the human Top1-DNA complex (PDB ID: 1K4T) by fitting the two molecules on the backbone atoms^11^. The substitution has done since the presence of a cleaved site in the 22 double strand present in the 1K4T X-ray structure that contains the topotecan molecule permits to investigate the ability of the compound 15 to interact with the LdTOP1LSDNA cleavable complex.

A grid of 60 × 60 × 60 with 0.375 Å were constructed around the protein DNA complex area using Autogrid v 4.2 software. The 2D structure of compounds was built using MOE-builder tool and minimized using MMFF94x force field. Docking analysis were done using Autodock v 4.2 software in which top 10 docked conformation were taken using Genetic Algorithm. Each docking calculation consist of 25 × 10^6^ energy evaluations with 250 population size. A mutation rate of 0.02 and a crossover rate of 0.8 were used to generate new docking trials.

### X-Ray crystal analysis

The compound **4** was crystallized by slow evaporation of its solution in dimethyl sulfoxide (DMSO) at room temperature, after seeding with its poor quality crystals grown in (N-Methyl-2-pyrrolidone (NMP). Crystal structure of **4** was determined by measuring X-ray diffraction data on a D8 Venture Bruker AXS single crystal X-ray diffractometer equipped with CMOS PHOTON 100 detector having monochromatised microfocus sources (Mo-Kα = 0.71073 Å) at room temperature. The structure was solved using *SHELX* program implemented in *APEX3*^32^,^33^. The non-H atoms were located in successive difference Fourier syntheses and refined with anisotropic thermal parameters. All the hydrogen atoms were placed at the calculated positions and refined using a riding model with appropriate HFIX commands. The program *Mercury* was used for molecular packing analysis^34^. Hirshfeld surface analysis was performed using *CrystalExplorer* package^35^. The structural details can be found from the deposited CIF with CCDC numbers 1451904.

### Pharmacophore Modelling

All the library compounds were sketched and energy minimized using MOE. A library database was constructed consisting of structures of compound **1–21** and EC_50_ values in MOE. This database was given as an input to pharmacophore elucidator and pharmacophore queries were searched. The best query on the basis of overlap score (Alignment score) was chosen.

## Additional Information

**How to cite this article**: Mamidala, R. *et al.* Identification of *Leishmania donovani* Topoisomerase 1 inhibitors *via* intuitive scaffold hopping and bioisosteric modification of known Top 1 inhibitors. *Sci. Rep.* **6**, 26603; doi: 10.1038/srep26603 (2016).

## Supplementary Material

Supplementary Information

Biology assays-1

## Figures and Tables

**Table 1:** Predicted physicochemical properties of **13**, CPT, EDT, RST and DFT.

Cpds	LogP	MW	HBD	HBA	TPSA	No. of Rotatable Bonds	Solubility (LogS_w_) [mmol/l]	Oral Bioavailability
13	1.43 ± 0.44	253.25	1	4	55.4	1	−3.64	% F(Oral) > 30%: 0.913% F(Oral) > 70%: 0.839
CPT	1.6 ± 0.65	348.35	1	6	79.73	1	−3.4	% F(Oral) > 30%: 0.698% F(Oral) > 70%: 0.187
DFT	2.04	398.36	1	6	79.73	1	−5.29	% F(Oral) > 30%: 0.637% F(Oral) > 70%: 0.187
EDT	1.92 ± 1.43	590.58	8	13	200.74	7	−4.15	% F(Oral) > 30%: 0.033% F(Oral) > 70%: 0.008
RST	3.11	284.31	0	3	33.2	0	−5.3	% F(Oral) > 30%: 0.811% F(Oral) > 70%: 0.379

**Table 2:**
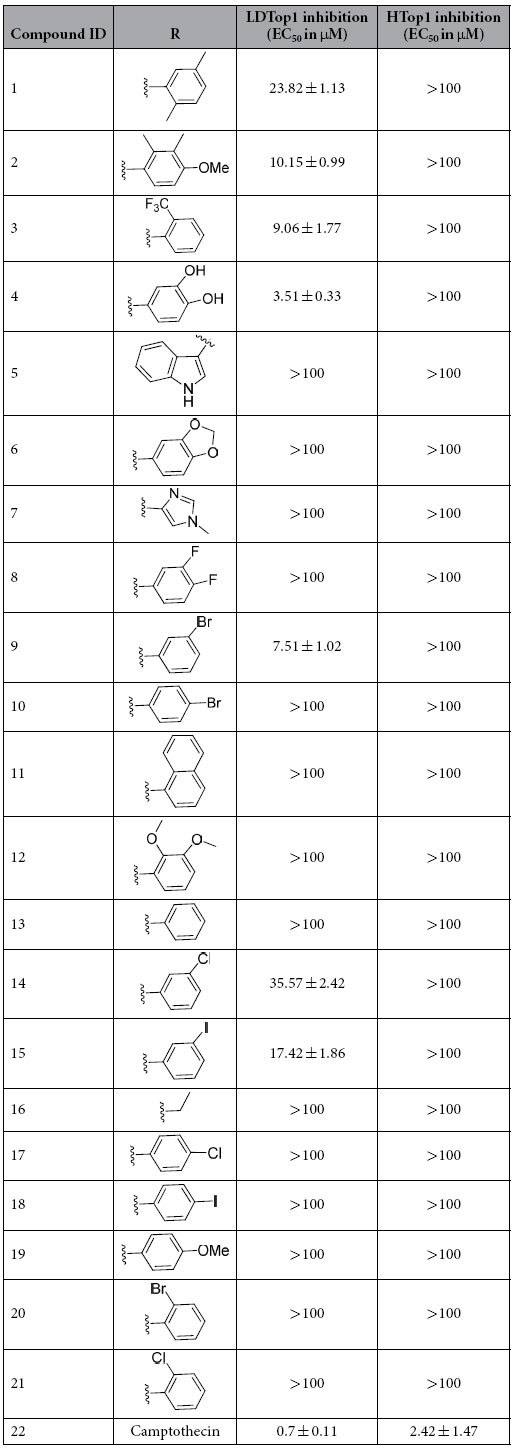
*In vitro* screening of furopyridinedione compounds against Human Top1 (HTop1) and Leishmania Top1 (LdTop1) in plasmid DNA relaxation experiment^a^.

^a^Data are averages of three individual experiments.

**Table 3:** *In vitro* antileishmanial activity of our compounds against extracellular promastigotes.

Compound #	Anti-promastigote activity IC_50_ (μM)^a^	Cytotoxicity CC_50_ on mammalian COS7 cell lines^b^	Selectivity Index (SI)^c^
**4**	4.21 ± 0.21	390.58 ± 3.15	~100

^a^IC_50_ values are average of two independent screenings expressed as an average ± standard error.

^b^CC_50_ on COS7 cells.

^c^Selectivity index is: CC_50_ against COS7/IC_50_ against promastigote.

^d^ND: Not determined.
