# Corrigendum: Identification of *Leishmania donovani* Topoisomerase 1 inhibitors *via* intuitive scaffold hopping and bioisosteric modification of known Top 1 inhibitors

**DOI:** 10.1038/srep28120

**Published:** 2016-06-20

**Authors:** Rajinikanth Mamidala, Papiya Majumdar, Kunal Kumar Jha, Chandramohan Bathula, Rahul Agarwal, M. Thirumala Chary, Hemanta K. Majumder, Parthapratim Munshi, Subhabrata Sen

Scientific Reports 6: Article number: 2660310.1038/srep26603; published online: 05252016; updated: 06202016

The original version of this Article contained errors in the spelling of the author Hemanta K. Majumder which was incorrectly given as H. K. Mazumdar. This has now been corrected in the PDF and HTML versions of the Article.

