# Supplementary material for: Identification of Leishmania donovani Topoisomerase 1 inhibitors via intuitive scaffold hopping and bioisosteric modification of known Top 1 inhibitors
Source: Sci Rep. 2016 May 25;6:26603. doi: 10.1038/srep26603 (PMC4879574; doi:10.1038/srep26603)
Supplement: Supplementary Information [file srep26603-s1.pdf]

Identification of *Leishmania donovani* Topoisomerase 1 inhibitors *via* intuitive scaffold hopping and bioisosteric modification of known Top 1 inhibitors

Rajinikanth Mamidala,[2, 5] Papiya Majumdar,[3] Kunal Kumar Jha,[1] Chandramohan Bathula,[1] Rahul Agarwal,[4] M. Thirumala Chary ,[2] H. K. Mazumdar[3], Parthapratim Munshi[1] and Subhabrata Sen*[, 1]

[1] Department of Chemistry, School of Natural Sciences, Shiv Nadar University, Chithera, Dadri, Gautam Buddha Nagar 201314, Uttar Pradesh, India

[2] Department of Chemistry, Jawaharlal Nehru Technological University, Kukatpally, Hyderabad 500085, Telangana, India

[3] Institute of Chemical Biology, 4 Raja S.C. Mullick Road, Kolkata 700032, West Bengal, India

[4] Department of Life Sciences, School of Natural Sciences, Shiv Nadar University, Chithera, Dadri, Gautam Buddha Nagar 201314, Uttar Pradesh, India

[5] GVK Bioscience, 28 A, Nacharam, Hyderabad 500076, Telangana

Contents

1. Materials and methods
    a. Chemistry
        i. General procedure for the synthesis of **1-21**
        ii. Individual synthesis and analytical data
    b. Molecular docking
        i. Docking studies of camptothecin, edotecarin, compound **4** and **13** with HTop 1
        ii. Docking studies of camptothecin, edotecarin, compound **4** and **13** with LdTop 1
2. ^{1}H and ^{13}C-NMR spectra

Materials and methods

Chemistry

All reactions were carried out in flame-dried sealed tubes with magnetic stirring. Unless otherwise noted, all experiments were performed under argon atmosphere. All reagents were purchased from Sigma Aldrich, Acros or Alfa Aesar. Solvents were treated with 4 Å molecular sieves or sodium and distilled prior to use. Purifications of reaction products were carried out by column chromatography using Chem Lab silica gel (230-400 mesh). Infrared spectra (IR) were recorded on a Thermoscientific Neoled IS5 FTIR spectrophotometer and are reported as wavelength numbers (cm^{-1}). Infrared spectra were recorded by preparing a KBr pellet containing the title compound. ^{1}H NMR and ^{13}C NMR spectra were recorded with tetramethylsilane (TMS) as internal standard at ambient temperature unless otherwise indicated on a Varian 300/400 and JEOL JNM-ECX500 MHz at 500 MHz for ^{1}H NMR and 100 MHz for ^{13}C NMR. Chemical shifts are reported in parts per million (ppm) and coupling constants are reported as Hertz (Hz). Splitting patterns are designated as singlet (s), broad singlet (bs), doublet (d), triplet (t). Splitting patterns that could not be interpreted or easily visualized are designated as multiple (m). The Mass Spectrometry analysis was done on the 6540 UHD Accurate-Mass Q-TOF LC/MS system (Agilent Technologies) equipped with Agilent 1290 LC system obtained by the Dept. of Chemistry, School of Natural Sciences, Shiv Nadar University, Uttar Pradesh 203207, India.

General procedure for the knoevenagel condensation between 6-methylfuro[3,4-c]pyridine-3,4(1H,5H)-dione and appropriate aldehydes

The reactions were executed in a 24 carousal (15 mL volume) custom made reaction block. To a stirred solution of 6-methylfuro[3,4-c]pyridine-3,4(1H,5H)-dione (150 mg, 0.9 mmoles) in ethanol (9 mL) added piperidine (7.5 mg, 0.09 mmol), followed by corresponding aldehyde (0.99 mmol) under N_2 atmosphere at room temperature, then the reaction mixture stirred at 80° C for 16 h, allowed to cool to room temperature. The supernatant was removed and the resulting precipitate was washed twice with ethanol (10 mL X 2) and once with diethyl ether (10 mL). The solvent removal and washing was conducted in parallel in Tecan Evo Freedom machine. They were then dried in genevac to provide the desired derivatives of 6-methylfuro[3,4-c]pyridine-3,4(1H,5H)- dione (**1**-**21**).

(Z)-1-(2,4-dimethylbenzylidene)-6-methylfuro[3,4-c]pyridine-3,4(1H,5H)-dione (**1**)

Following the general protocol 2, 4-dimethylbenzaldehyde (134 mg, 0.99 mmol) afforded **1** in 127 mg (yield 48%). ^{1}H NMR (300 MHz; DMSO-d$_6$): δ 12.21 (br. s, 1H), 7.81 (m, 1H), 7.23-7.19 (m, 1H); 7.17-7.15 (m, 1H); 6.98-6.85 (m, 2H), 2.36 (s, 3H), 2.35-2.34 (m, 6H). ^{13}C NMR (125 MHz; DMSO-d$_6$): δ 164.84, 157.98, 156.65, 155.67, 143.16, 135.61, 135.39, 131.38, 130.95, 130.52, 109.28, 106.01, 97.79, 21.15, 19.92, 19.72. LCMS (ESI) m/z: $[M + H]^+$ calculated for ($C_{17}H_{16}NO_3$) 282.11, found 282.27.

(Z)-1-(4-methoxy-2,3-dimethylbenzylidene)-6-methylfuro[3,4-c]pyridine-3,4(1H,5H)-dione (**2**)

Following the general protocol 4-methoxy-2, 3-dimethylbenzaldehyde (162 mg, 0.99 mmol) afforded **2** in 200 mg (yield 72%). ^{1}H NMR (300 MHz; DMSO-d$_6$): δ 12.15 (br. s, 1H), 7.85-7.83 (d, *J* = 6 Hz, 1H), 7.01-6.95 (m, 3H); 3.81 (s, 3H), 2.27-2.26 (d, J = 3Hz, 6H), 2.18 (s, 3H). ^{13}C NMR (125 MHz; DMSO-d$_6$): δ 164.90, 158.29, 156.71, 155.11, 141.76, 138.47, 129.06, 124.98, 123.96, 110.47, 108.88, 105.45, 97.42, 56.04, 20.38, 16.39, 12.32. LCMS (ESI) m/z: $[M + H]^+$ calculated for ($C_{18}H_{19}NO_4$) 312.58, found 312.25.

(Z)-1-(2-trifluoromethylbenzylidene)-6-methylfuro[3,4-c]pyridine-3,4(1H,5H)-dione (**3**)

Following the general protocol 2-trifluoromethylbenzaldehyde (168 mg, 0.99 mmol) afforded **3** in 200 mg (yield 71%). ^{1}H NMR (300 MHz; DMSO-d$_6$): δ 11.99-11.80 (br. s, 1H), 8.20-8.18 (d, *J* = 6 Hz, 1H), 7.86-7.78 (m, 2H), 7.61-6.58 (m, 1H), 6.85 (s, 1H), 6.80 (s, 1H), 2.34 (s, 3H). ^{13}C NMR (125 MHz; DMSO-d$_6$): δ 164.19, 157.98, 157.02, 156.09, 145.45, 133.29, 132.57, 130.64, 129.69, 127.56, 127.31, 126.64, 106.59, 105.27, 97.52, 19.96. LCMS (ESI) m/z: $[M + H]^+$ calculated for ($C_{16}H_{11}F_3NO_3$) 322.06, found 322.00.

(Z)-1-(3,4-dihydroxybenzylidene)-6-methylfuro[3,4-c]pyridine-3,4(1H,5H)-dione (**4**)

Following the general protocol 3,4-dihydroxybenzaldehyde (136 mg, 0.99 mmol) afforded **4** in 132 mg (yield 53%). ^{1}H NMR (300 MHz; DMSO-d$_6$): δ 7.39 (s, 1H), 7.16-7.05 (m, 1H), 6.80-6.75 (m, 2H), 6.72 (s, 1H), 2.28 (s, 3H). ^{13}C NMR (125 MHz; DMSO-d$_6$): δ 168.75, 167.43, 164.90, 158.35, 156.71, 155.05, 148.29, 146.05, 140.58, 124.67, 124.40, 117.66, 116.45, 113.39,

109.28, 105.14, 99.84, 97.03, 68.28, 20.00, 19.65. LCMS (ESI) m/z: $[M + H]^+$ calculated for ($C_{15}H_{12}NO_5$) 286.06, found 386.00.

(Z)-1-((1H-indol-3-yl)methylene)-6-methylfuro[3,4-c]pyridine-3,4(1H,5H)-dione (**5**)

Following the general protocol 1H-indole-3-carbaldehyde (145 mg, 0.99 mmol) afforded **5** in 192 mg (yield 71%). ^{1}H NMR (500 MHz; DMSO-d_6): δ 11.99 (br. s, 1H), 8.01 (m, 2H), 7.31-7.25 (m, 1H); 7.39 (s, 1H); 7.21-7.18 (m, 3H), 6.82 (s, 1H), 2.39 (s, 3H). ^{13}C NMR (125 MHz; DMSO-d_6): δ 168.77, 167.41, 164.88, 158.56, 158.38, 155.79, 155.05, 154.35, 139.38, 136.44, 130.69, 126.96, 123.02, 121.01, 119.26, 112.72, 109.59, 109.29, 106.85, 104.80, 99.84, 96.94, 68.29, 19.99, 19.65. HRMS (ESI-TOF) m/z: $[M + H]^+$ calculated for ($C_{17}H_{13}N_2O_3$) 293.08, found 293.00.

(Z)-1-(benzo[d][1,3]dioxol-5-ylmethylene)-6-methylfuro[3,4-c]pyridine-3,4(1H,5H)-dione (**6**)

Following the general protocol benzo[d][1,3]dioxole-5-carbaldehyde (150 mg, 0.99 mmol) afforded **6** in 200 mg (yield 75%). ^{1}H NMR (300 MHz; DMSO-d_6): δ 12.18 (bs, 1H), 7.40 (s, 1H), 7.35-7.33 (d, J = 6 Hz, 1H), 7.10-7.05 (m, 1H), 6.89 (s, 1H), 6.64-6.63 (d, J = 3 Hz, 1H), 6..05 (s, 2H), 2.34 (s, 3H). ^{13}C NMR (125 MHz; DMSO-d_6): δ 164.55, 158.19, 156.64, 155.50, 148.90, 148.33, 141.64, 127.38, 126.64, 112.13, 109.73, 109.39, 105.50, 102.18, 97.05, 20.02. LCMS (ESI) m/z: $[M + H]^+$ calculated for ($C_{16}H_{12}NO_5$) 298.06, found 298.18.

(Z)-6-methyl-1-((1-methyl-1H-imidazol-4-yl)methylene)furo[3,4-c]pyridine-3,4(1H,5H)-dione (**7**)

Following the general protocol 1-methyl-1H-imidazole-4-carbaldehyde (109 mg, 0.99 mmol) afforded **7** in 182 mg (yield 79%). ^{1}H NMR (500 MHz; DMSO-d_6): δ 11.98 (br. s, 1H), 8.03 (m, 1H), 7.61-7.55 (m, 1H); 7.49 (s, 1H); 6.86 (s, 1H), 3.92 (s, 3H), 2.31 (s, 3H). ^{13}C NMR (125 MHz; DMSO-d_6): δ 164.36, 158.08, 155.29, 153.91, 138.86, 136.50, 133.84, 127.01, 122.71, 120.83, 118.90, 110.64, 108.20, 105.93, 96.48. HRMS (ESI-TOF) m/z: $[M + H]^+$ calculated for ($C_{13}H_{12}N_3O_3$) 258.08, found 258.19.

(Z)-1-(3, 4-difluorobenzylidene)-6-methylfuro[3,4-c]pyridine-3,4(1H,5H)-dione (**8**)

Following the general protocol 3,4-difluorobenzaldehyde (140 mg, 0.99 mmol) afforded **8** in 220 mg (yield 85%). ^{1}H NMR (300 MHz; DMSO-d$_6$): δ 12.25 (bs, 1H), 7.85-7.79 (m, 1H), 7.62-7.55 (m, 2H), 6.99 (s, 1H), 6.62 (s, 1H), 2.34 (s, 3H). ^{13}C NMR (125 MHz; DMSO-d$_6$): δ 164.21, 158.00, 156.47, 156.26, 143.69, 130.88, 128.11, 118.99, 118.85, 118.76, 118.62, 109.32, 106.00, 97.23, 20.07. LCMS (ESI) m/z: $[M + H]^+$ calculated for ($C_{15}H_{10}F_2NO_3$) 290.05, found 290.17.

(Z)-1-(3-bromobenzylidene)-6-methylfuro[3,4-c]pyridine-3,4(1H,5H)-dione (**9**)

Following the general protocol 3-bromobenzaldehyde (182 mg, 0.99 mmol) afforded **9** in 178 mg (yield 60%). ^{1}H NMR (300 MHz; DMSO-d$_6$): δ 12.41-12.21 (bs, 1H), 8.00 (s, 1H), 7.80-7.75 (d, J = 15 Hz, 1H), 7.61-7.56 (m, 1H), 7.48-7.40 (m, 1H), 6.99 (s, 1H), 6.68 (s, 1H), 2.31 (s, 3H). ^{13}C NMR (125 MHz; DMSO-d$_6$): δ 168.30, 158.04, 156.54, 156.27, 144.10, 135.53, 132.74, 132.23, 131.52, 129.71, 122.61, 109.86, 106.10, 97.35, 56.50, 20.10, 19.01. LCMS (ESI) m/z: $[M + H]^+$ calculated for ($C_{15}H_{11}BrNO_3$) 332.1488, found 332.0950.

(Z)-1-(4-bromobenzylidene)-6-methylfuro[3,4-c]pyridine-3,4(1H,5H)-dione (**10**)

Following the general protocol 4-bromobenzaldehyde (182 mg, 0.99 mmol) afforded **10** in 192 mg (yield 64%). ^{1}H NMR (300 MHz; DMSO-d$_6$): δ 12.35-12.25 (bs, 1H), 7.79-7.81 (m, 4H), 6.98 (s, 1H), 6.67 (s, 1H), 2.36 (s, 3H). ^{13}C NMR (125 MHz; DMSO-d$_6$): δ 164.37, 158.10, 156.61, 156.11, 143.67, 132.58, 132.49, 132.44, 123.18, 110.45, 106.02, 97.34, 20.09. LCMS (ESI) m/z: $[M + H]^+$ calculated for ($C_{15}H_{11}BrNO_3$) 332.1488, found 332.1330.

(Z)-6-methyl-1-(naphthalen-2-ylmethylene)furo[3,4-c]pyridine-3,4(1H,5H)-dione (**11**)

Following the general protocol 3-naphthaldehyde (152 mg, 0.99 mmol) afforded **11** in 231 mg (yield 84%). ^{1}H NMR (300 MHz; DMSO-d$_6$): δ 12.23 (bs, 1H), 8.59-8.42 (m, 1H), 8.25-8.19 (m, 1H), 8.01-7.95 (m, 2H), 7.65-7.52 (m, 4H), 7.19 (s, 1H), 2.36 (s, 3H). ^{13}C NMR (125 MHz; DMSO-d$_6$): δ 164.75, 158.31, 156.75, 155.92, 144.54, 133.84, 131.77, 130.29, 130.24, 129.77, 129.11, 127.42, 126.87, 126.17, 124.32, 107.50, 105.94, 97.93, 20.17. LCMS (ESI) m/z: $[M + H]^+$ calculated for ($C_{19}H_{14}NO_3$) 304.09, found 304.23.

(Z)-1-(2,3-dimethoxybenzylidene)-6-methylfuro[3,4-c]pyridine-3,4(1H,5H)-dione (**12**)

Following the general protocol 2,3-dimethoxybenzaldehyde (164 mg, 0.99 mmol) afforded **12** in 220 mg (yield 78%). ^{1}H NMR (300 MHz; DMSO-d$_6$): δ 12.20 (bs, 1H), 7.40 (s, 1H), 7.64-7.62 (m, 1H), 7.21-7.15 (m, 2H), 6.95 (s, 1H), 6.88 (s, 1H) 3.80 (s, 6H), 2.31)s, 1H). LCMS (ESI) m/z: $[M + H]^+$ calculated for ($C_{15}H_{12}NO_5$) 314.09, found 314.21.

(Z)-1-benzylidene-6-methylfuro[3,4-c]pyridine-3,4(1H,5H)-dione (**13**)

Following the general protocol benzaldehyde (105 mg, 0.99 mmol) afforded **13** in 163 mg (yield 74%). ^{1}H NMR (500 MHz; DMSO-d$_6$): δ 12.00 (br. s, 1H), 7.82-7.78 (m, 2H), 7.59-7.39 (m, 3H), 6.99 (s, 1H); 6.79 (s, 1H), 2.31 (s, 3H). ^{13}C NMR (125 MHz; DMSO-d$_6$): δ 164.59, 158.17, 156.78, 155.94, 143.19, 133.21, 130.86, 129.83, 129.49, 111.82, 105.95, 97.34, 20.20. HRMS (ESI-TOF) m/z: $[M + H]^+$ calculated for ($C_{15}H_{12}NO_3$) 254.07, found 254.14.

(Z)-1-(3-chlorobenzylidene)-6-methylfuro[3,4-c]pyridine-3,4(1H,5H)-dione (**14**)

Following the general protocol 3-chlorobenzaldehyde (140 mg, 0.99 mmol) afforded **14** in 229 mg (yield 80%). ^{1}H NMR (400 MHz; DMSO-d$_6$): δ 12.25 (br. s, 1H), 7.84-7.82 (m, 2H), 7.78-7.71 (m, 1H), 7.58-7.42 (m, 2H), 6.99 (s, 1H), 6.74 (s, 1H) 2.36 (s, 3H). ^{13}C NMR (100 MHz; DMSO-d$_6$): δ 163.84, 157.58, 156.08, 155.81, 143.68, 134.80, 133.57, 130.81, 129.37, 128.91, 109.46, 105.66, 96.89, 19.64. UPLC m/z: $[M + H]^+$ calculated for ($C_{16}H_{10}ClNO_3$) 288.03, found 288.01.

(Z)-1-(3-iodobenzylidene)-6-methylfuro[3,4-c]pyridine-3,4(1H,5H)-dione (**15**)

Following the general protocol 3-iodobenzaldehyde (231 mg, 0.99 mmol) afforded **19** in 347 mg (yield 92%). ^{1}H NMR (400 MHz; DMSO-d$_6$): δ 12.21 (br. s, 1H), 8.19-8.18 (d, J = 4 Hz, 1H), 7.81-7.75 (m, 2H), 7.35-7.25 (m, 1H), 6.96 (s, 1H), 6.71 (s, 1H) 2.37 (s, 3H). ^{13}C NMR (100 MHz; DMSO-d$_6$): δ 164.39, 158.09, 156.62, 156.24, 143.95, 138.77, 138.11, 135.52, 131.52, 130.06, 109.97, 106.12, 97.38, 95.88, 20.16. UPLC m/z: $[M + H]^+$ calculated for ($C_{15}H_{11}INO_3$) 379.97, found 380.00.

(Z)-6-methyl-1-propylidenefuro[3,4-c]pyridine-3,4(1H,5H)-dione (**16**)

Following the general protocol propionaldehyde (60 mg, 0.99 mmol) afforded **16** in 347185 mg (yield 90%). ^{1}H NMR (400 MHz; DMSO-d$_6$): δ 11.99 (br. s, 1H), 6.61 (s, 1H), 6.08-6.00 (m,

1H), 2.39-2.35 (m, 2H), 2.11 (s, 3H), 1.18-1.08 (m, 3H). UPLC m/z: $[M + H]^+$ calculated for ($C_{11}H_{12}NO_3$) 206.07, found 206.

(Z)-1-(4-chlorobenzylidene)-6-methylfuro[3,4-c]pyridine-3,4(1H,5H)-dione (**17**)

Following the general protocol 4-chlorobenzaldehyde (138 mg, 0.99 mmol) afforded **17** in 215 mg (yield 75%). ^{1}H NMR (400 MHz; DMSO-d_6): δ 12.22 (br. s, 1H), 7.83-7.80 (d, *J* = 12 Hz, 2H), 7.60-7.57 (d, *J* = 12 Hz, 2H), 6.96 (s, 1H), 6.72 (s, 1H), 2.35 (s, 3H). ^{13}C NMR (125 MHz; DMSO-d_6): δ 164.44, 158.15, 156.69, 156.17, 143.65, 134.36, 132.45, 132.18, 129.65, 110.43, 106.07, 97.39, 20.14. UPLC m/z: $[M + H]^+$ calculated for ($C_{15}H_{11}ClNO_3$) 288.03, found 288.04

(Z)-1-(4-iodobenzylidene)-6-methylfuro[3,4-c]pyridine-3,4(1H,5H)-dione (**18**)

Following the general protocol 4-iodobenzaldehyde (228 mg, 0.99 mmol) afforded **18** in 265 mg (yield 70%). ^{1}H NMR (400 MHz; DMSO-d_6): δ 12.18 (br. s, 1H), 7.85-7.81 (d, *J* = 16 Hz, 2H), 7.60-7.56 (d, *J* = 12 Hz, 2H), 6.91 (s, 1H), 6.74 (s, 1H), 2.31 (s, 3H). ^{13}C NMR (100 MHz; DMSO-d_6): δ 163.94, 157.66, 155.65, 143.30, 137.92, 132.23, 132.06, 110.29, 105.57, 96.9, 19.65. UPLC m/z: $[M + H]^+$ calculated for ($C_{15}H_{11}INO_3$) 379.97, found 380.01

(Z)-1-(4-methoxybenzylidene)-6-methylfuro[3,4-c]pyridine-3,4(1H,5H)-dione (**19**)

Following the general protocol 4-methoxybenzaldehyde (135 mg, 0.99 mmol) afforded **19** in 237 mg (yield 84%). ^{1}H NMR (400 MHz; DMSO-d_6): δ 12.15 (br. s, 1H), 7.82-7.76 (d, *J* = 18 Hz, 2H), 7.18-7.12 (d, *J* = 17.8 Hz, 2H), 6.95 (s, 1H), 6.75 (s, 1H), 3.81 (s, 3H), 2.34 (s, 3H). ^{13}C NMR (125 MHz; DMSO-d_6): δ 164.27, 160.24, 157.80, 156.26, 154.91, 140.98, 132.28, 125.38, 114.62, 111.69, 105.01, 96.61, 55.34, 19.57. UPLC m/z: $[M + H]^+$ calculated for ($C_{16}H_{13}NO_4$) 284.08, found 284.11.

(Z)-1-(2-bromobenzylidene)-6-methylfuro[3,4-c]pyridine-3,4(1H,5H)-dione (**20**)

Following the general protocol 2-bromobenzaldehyde (180 mg, 0.99 mmol) afforded **20** in 310 mg (yield 94%). ^{1}H NMR (400 MHz; DMSO-d_6): δ 12.31 (br. s, 1H), 8.15-8.12 (m, 1H), 7.80-7.74 (m, 1H), 7.58-7.55 (m, 1H), 7.39-7.32 (m, 1H), 6.95 (s, 1H), 6.94 (s, 1H), 2.32 (s, 3H). ^{13}C NMR (125 MHz; DMSO-d_6): δ 167.04, 157.87, 156.23, 144.34, 133.47, 132.19, 130.87, 128.24,

124.58, 108.86, 105.88, 97.30, 20.03. UPLC m/z: $[M + H]^+$ calculated for ($C_{15}H_{11}BrNO_3$) 331.98, found 332.01.

(Z)-1-(2-chlorobenzylidene)-6-methylfuro[3,4-c]pyridine-3,4(1H,5H)-dione (**21**)

Following the general protocol 2-chlorobenzaldehyde (137 mg, 0.99 mmol) afforded **21** in 210 mg (yield 73%). ^{1}H NMR (400 MHz; DMSO-d_6): δ 12.31 (br. s, 1H), 8.15-8.12 (m, 1H), 7.60-7.40 (m, 3H), 6.96 (s, 1H), 6.91 (s, 1H), 2.31 (s, 3H). ^{13}C NMR (125 MHz; DMSO-d_6): δ 164.30, 158.05, 156.64, 156.37, 144.98, 133.80, 132.09, 131.18, 130.78, 130.32, 128.19, 106.32, 106.09, 97.64, 20.00. UPLC m/z: $[M + H]^+$ calculated for ($C_{15}H_{11}ClNO_3$) 288.03, found 288.04.

Docking Studies with HumanTOP1

Results & Discussion

According to the docking results, the binding energy (kcal/mol) of the best pose of compounds with receptor HumanTOP1 are:

Compound Name	HumanTOP1
Edotecarin	-9.31
**13**	-7.70

All the compounds intercalates at the DNA cleavage site (Figure 2) and forms various bonded and non-bonded interactions as shown in Table and Figure 2 & 3.

Compound Name	Hydrogen Bonds Interactions	Non-bonded Interactions
Camptothecin (PDB ID : 1T8I)	ARG364	THR718, ASN722, DG12, DA113, DC112, TGP11
Edotecarin	ARG364, ASN352, TGP11, DG12, DA113, DC112	ALA351, TYR426, ASP533, ASN722, DT10
**13**	ARG364, THR718, ASN722	DT10, DC112, DA113, TGP11

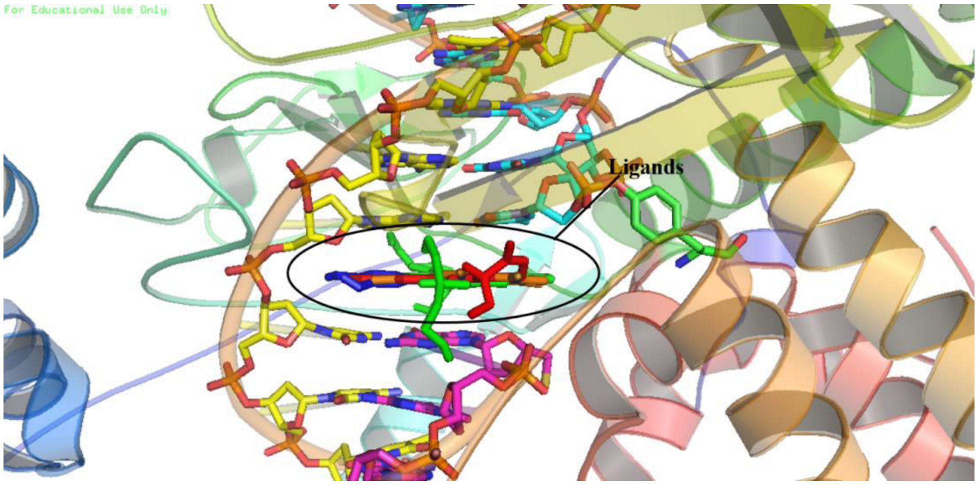

Figure 1: This figure shows the binding pose of docked compounds with HumanTOP1. Campthothecin in Red, Edotercarin in Green, **4** in Blue and **RM27** in Orange.

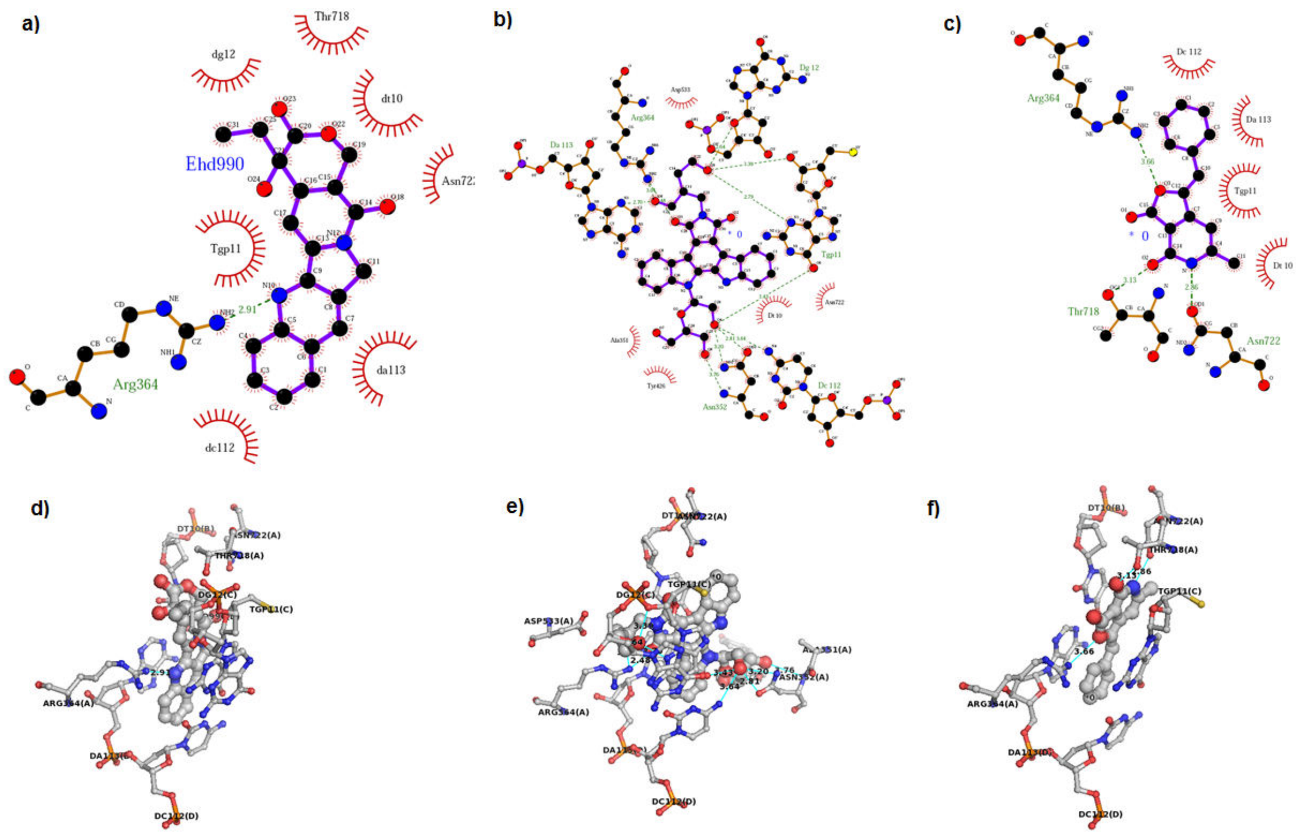

Figure 2. (a) Binding pose of Campthothecin in 2D; (b) Binding pose of Edotercarin in 2D; (c) Binding pose of **13** in 2D; (d) Binding pose of Camptothecin in 3D; (e) Binding pose of Edotercarin in 3D; (f) Binding pose of **13** in 3D.

Docking Studies with Ld TOP1

Results & Discussion

According to the docking results, the binding energy (kcal/mol) of the best pose of compounds with receptor LdTOP1 complex human Top1-DNA substituted are:

**Compound Name**	**LdTOP1**
Camptothecin	-10.01
Edotecarin	-11.15
**4**	-8.66
**13**	-8.07

All the compounds intercalate at the DNA cleavage site (Figure 3) and form various bonded and non-bonded interactions as shown in Table and Figure 4.

**Compound Name**	**Hydrogen Bonds Interactions**	**Non-bonded Interactions**
Camptothecin	ARG190, THR217, HIS453, GLN454, DG12	ASN221, ARG314, ASP353, ILE355, DT10, DA113, TGP11
Edotecarin	ARG190, GLN454, DT10, TGP11, DG12	ASN178, ILE220, ASN221, ASP353, DC112, DA113

**4**	ARG190, ASN221, DA113	THR217, DT10, TGP11, DC112
**13**	ARG190, GLN454, TGP11	ASN221, DT10, DG12

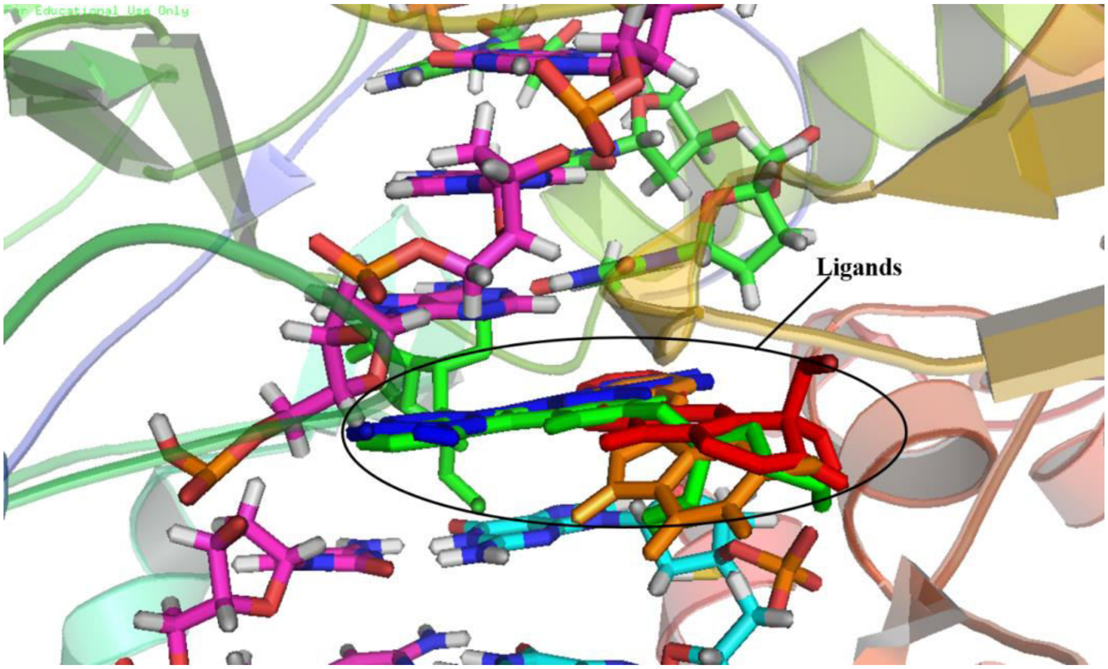

Figure 3: This figure shows the binding pose of docked compounds with LdTOP1. Campthothecin in Red, Edotercarin in Green, **4** in Blue and **13** in Orange.

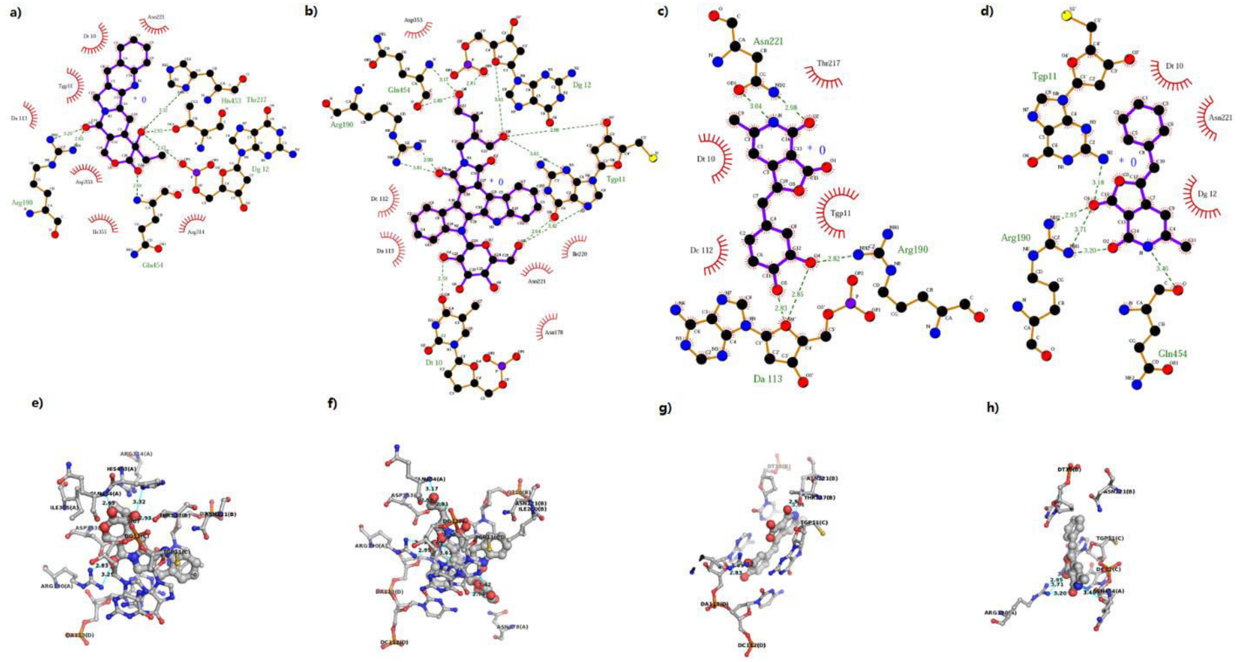

Figure 4. (a) Binding pose of Campthothecin in 2D; (b) Binding pose of Edotercarin in 2D; (c) Binding pose of **4** in 2D; (d) Binding pose of **13** in 2D; (e) Binding pose of Camptothecin in 3D; (f) Binding pose of Edotercarin in 3D; (g) Binding pose of **4** in 3D; (h) Binding pose of **13** in 3D.

Compound 1

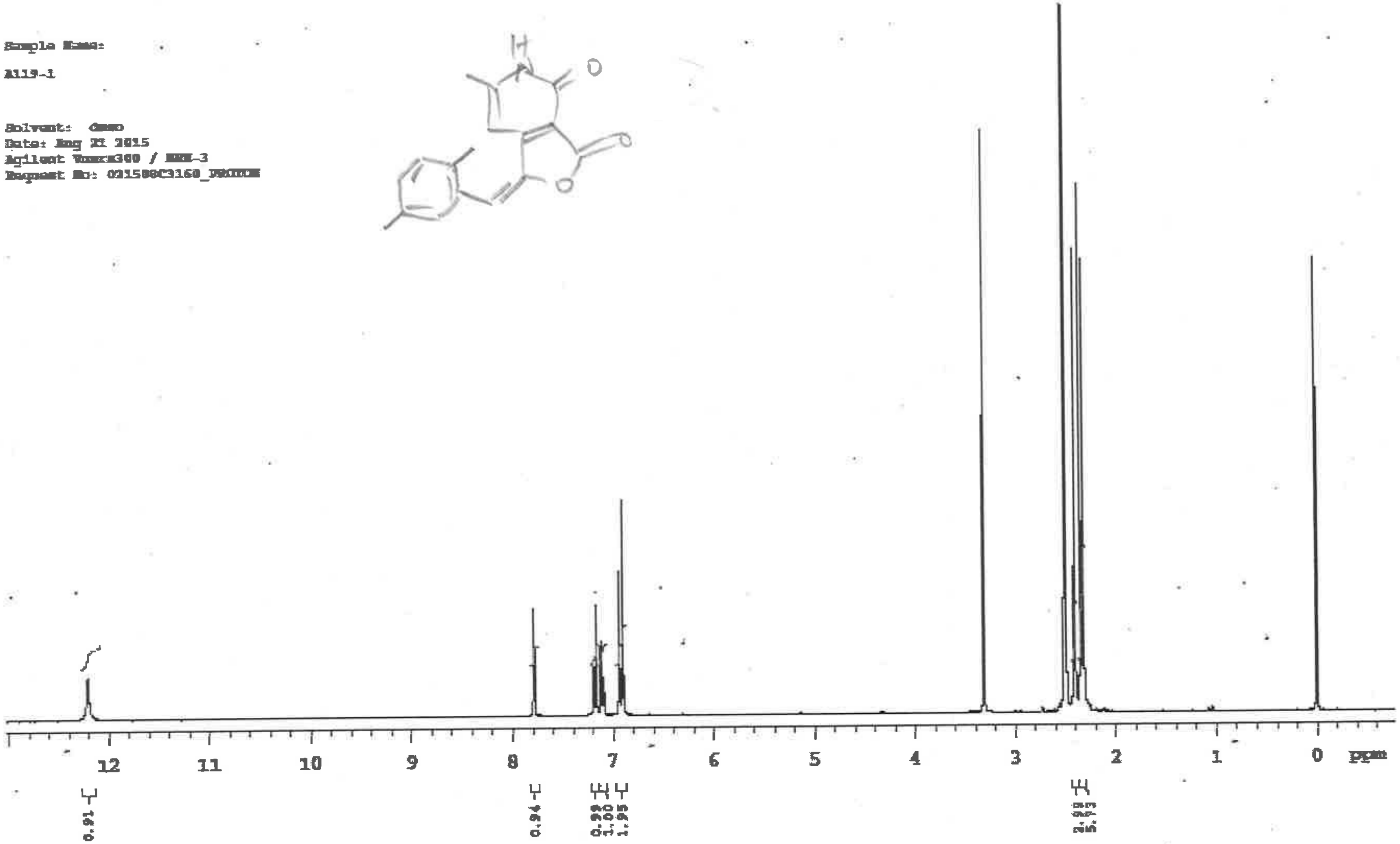

Plotname: 021509C3160_PROTON_01_plot01

Compound 2

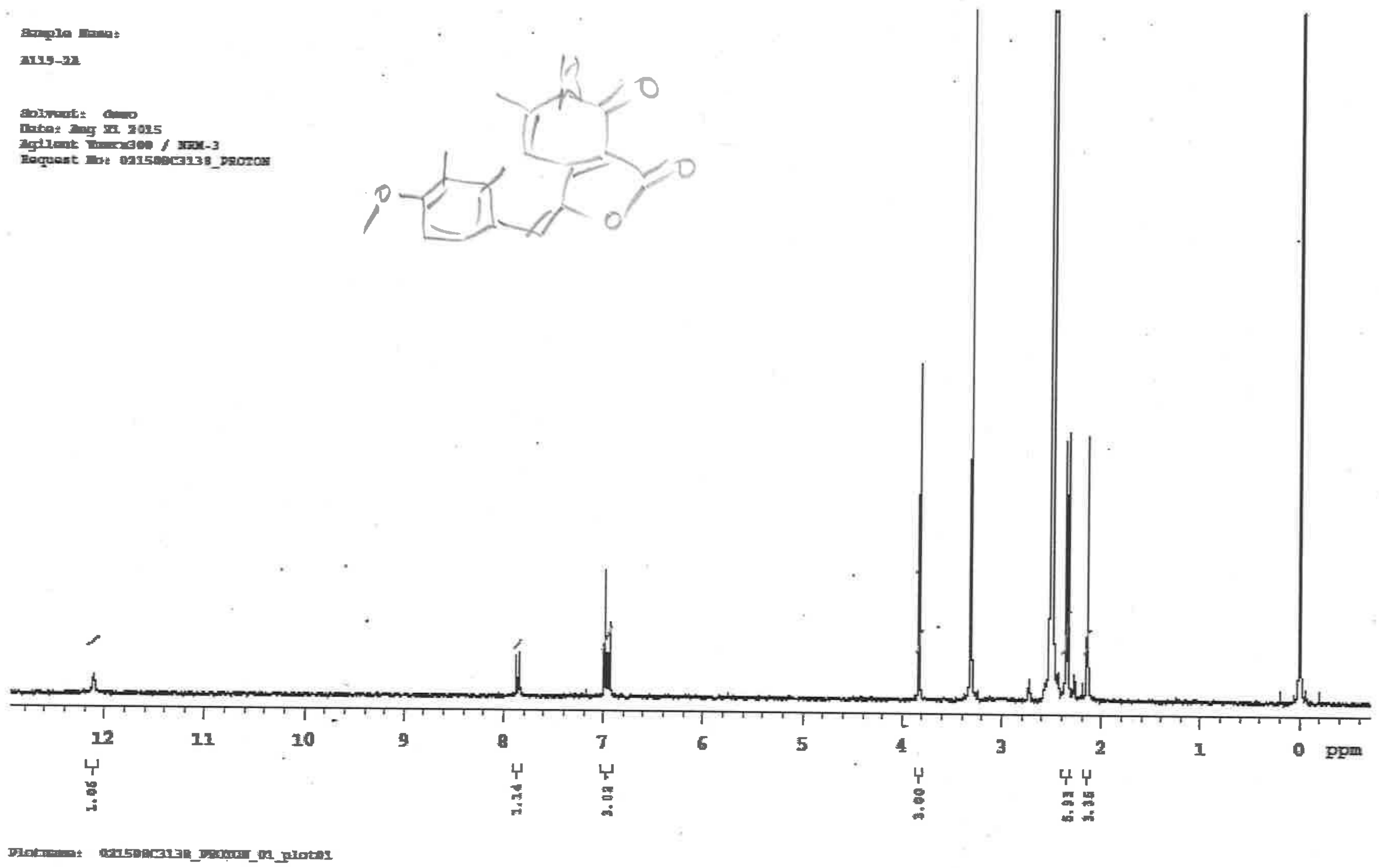
Sample Name:
A119-3A
Solvent: dmso
Date: Aug 31 2015
Agilent Vnmrs300 / NRM-3
Request No: 021508C3138_PROTON
12
11
10
9
8
7
6
5
4
3
2
1
0
ppm
1.06
1.14
3.02
3.00
Plotname: 021508C3138_PROTON_01_plot01

Compound 3

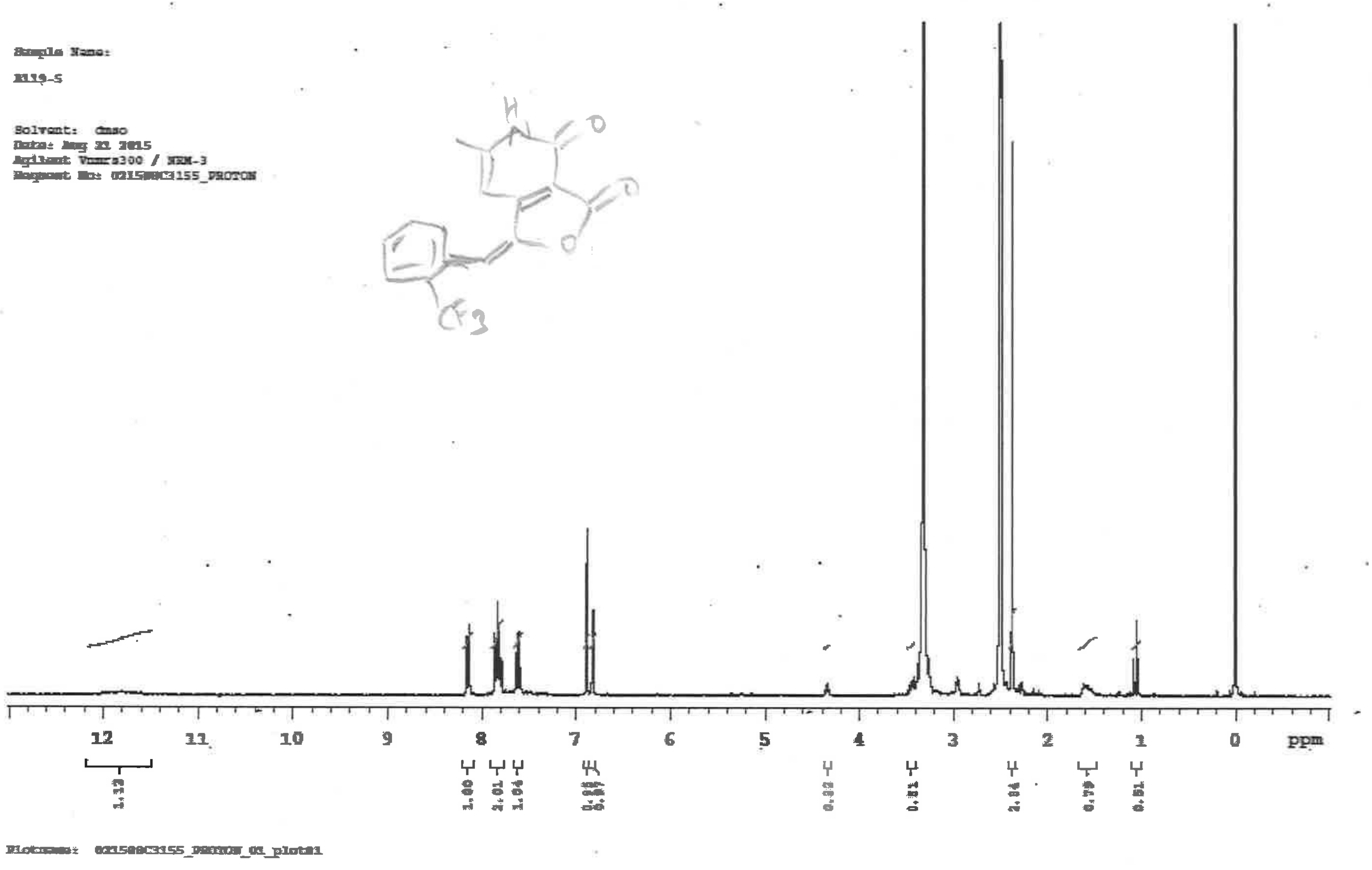

Compound 4

Sample Name:

A120-6

Solvent: dmso
Date: Aug 21 2015
Agilent Vnmrs300 / NMR-3
Request No: 021508C3147_PROTON

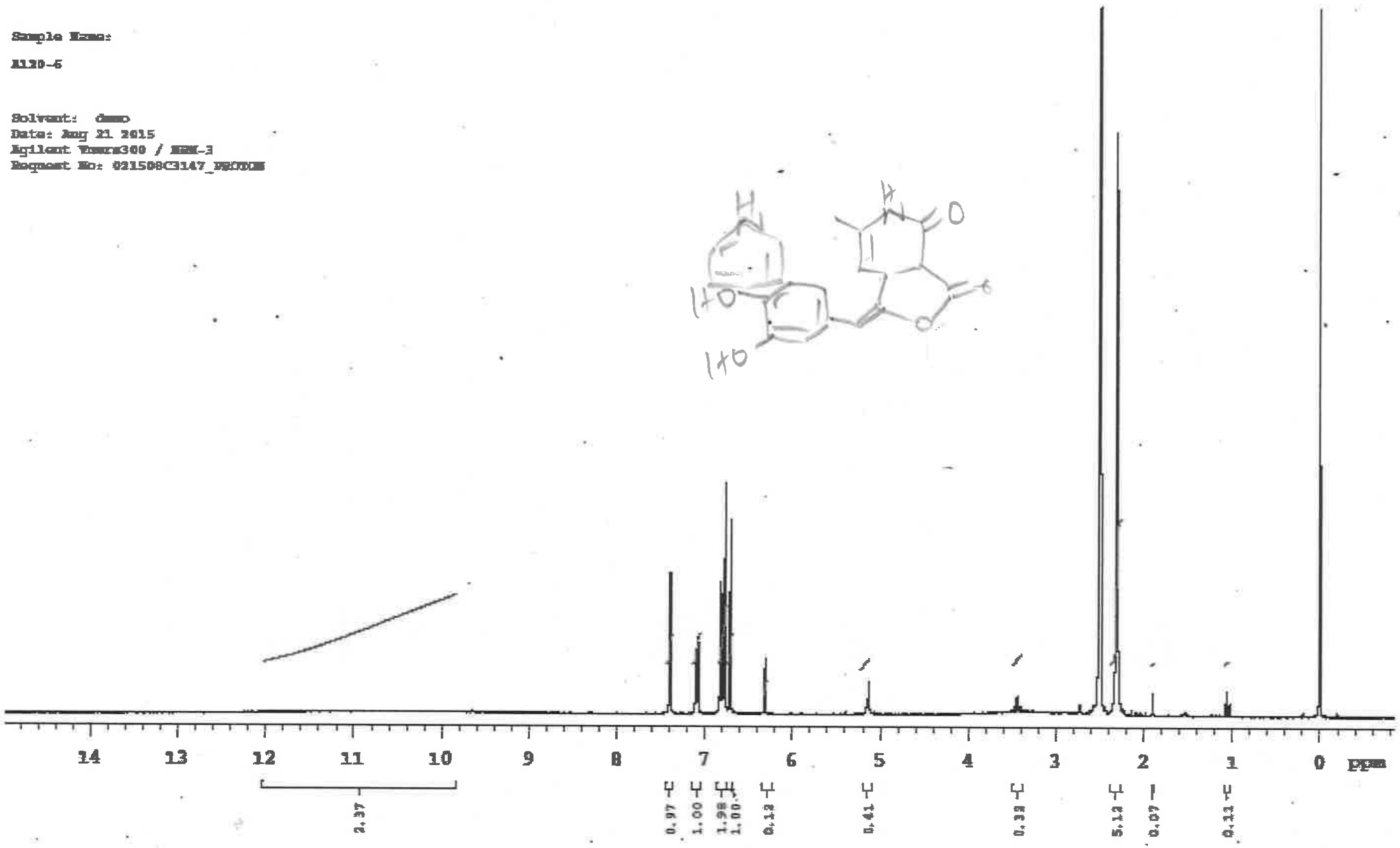

Plotname: 021508C3147_PROTON_01_plot01

Compound 5

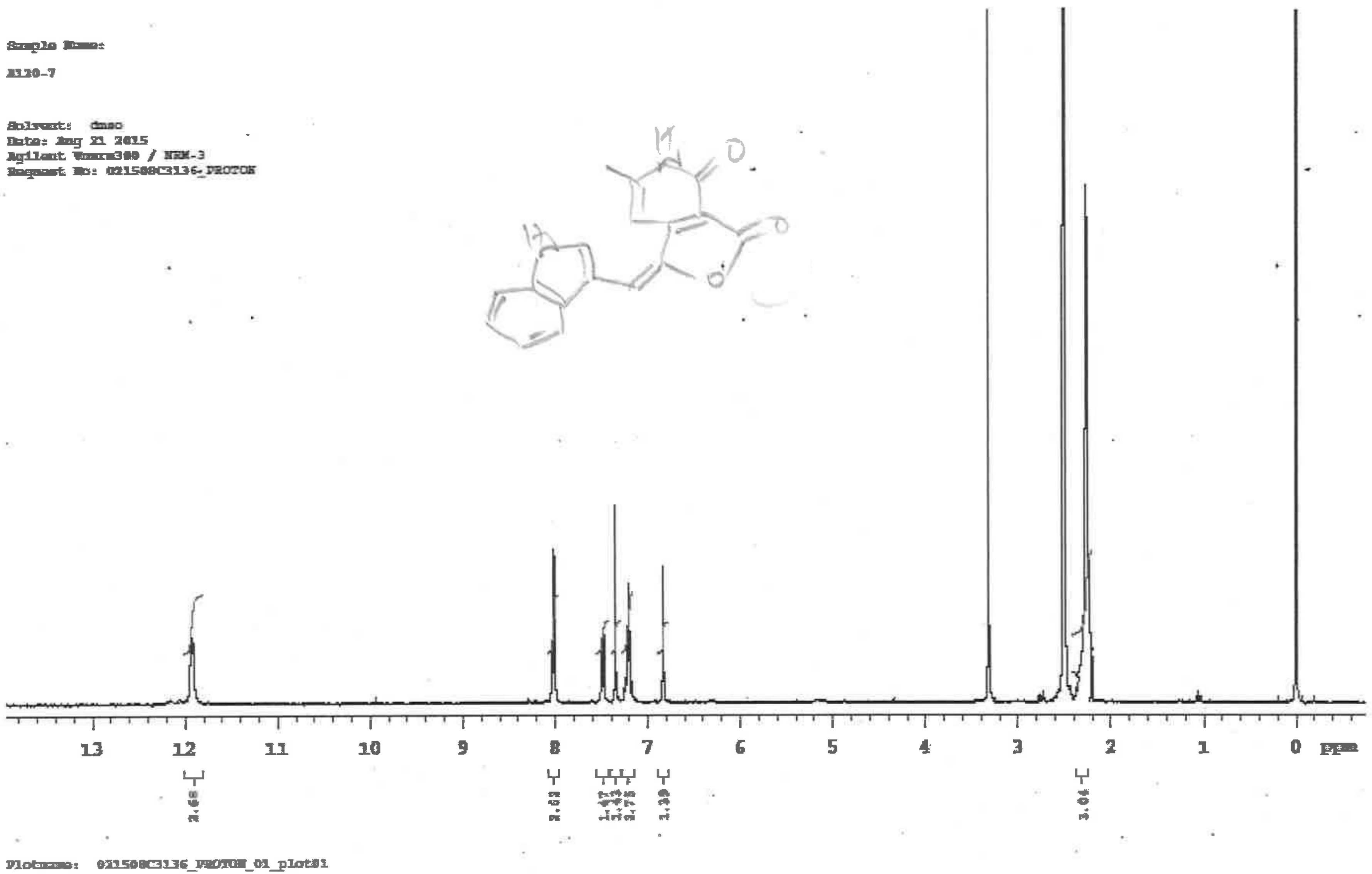
Sample Name:
A130-7
Solvent: dmso
Date: Aug 21 2015
Agilent Vnmrs300 / NRM-3
Request No: 021508C3136_PROTON
13
12
11
10
9
8
7
6
5
4
3
2
1
0 ppm
0.68
2.02
1.47
1.43
2.75
1.30
3.04
Plotname: 021508C3136_PROTON_01_plot01

Compound 6

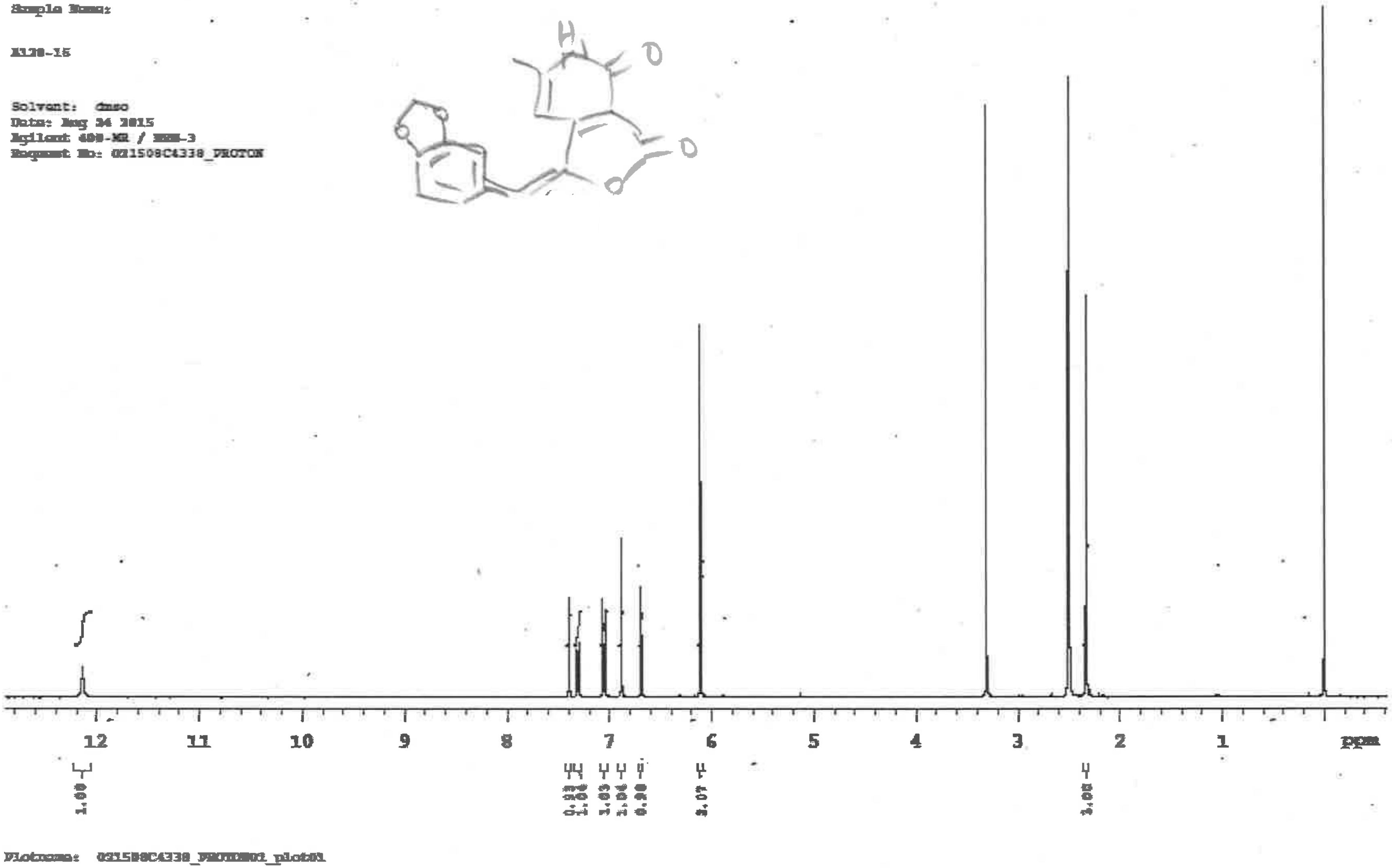
Sample Name:
Solvent: dmso
Date: Aug 24 2015
Request No: 021508C4338_PROTON
H
O
O
O
12
11
10
9
8
7
6
5
4
3
2
1
ppm

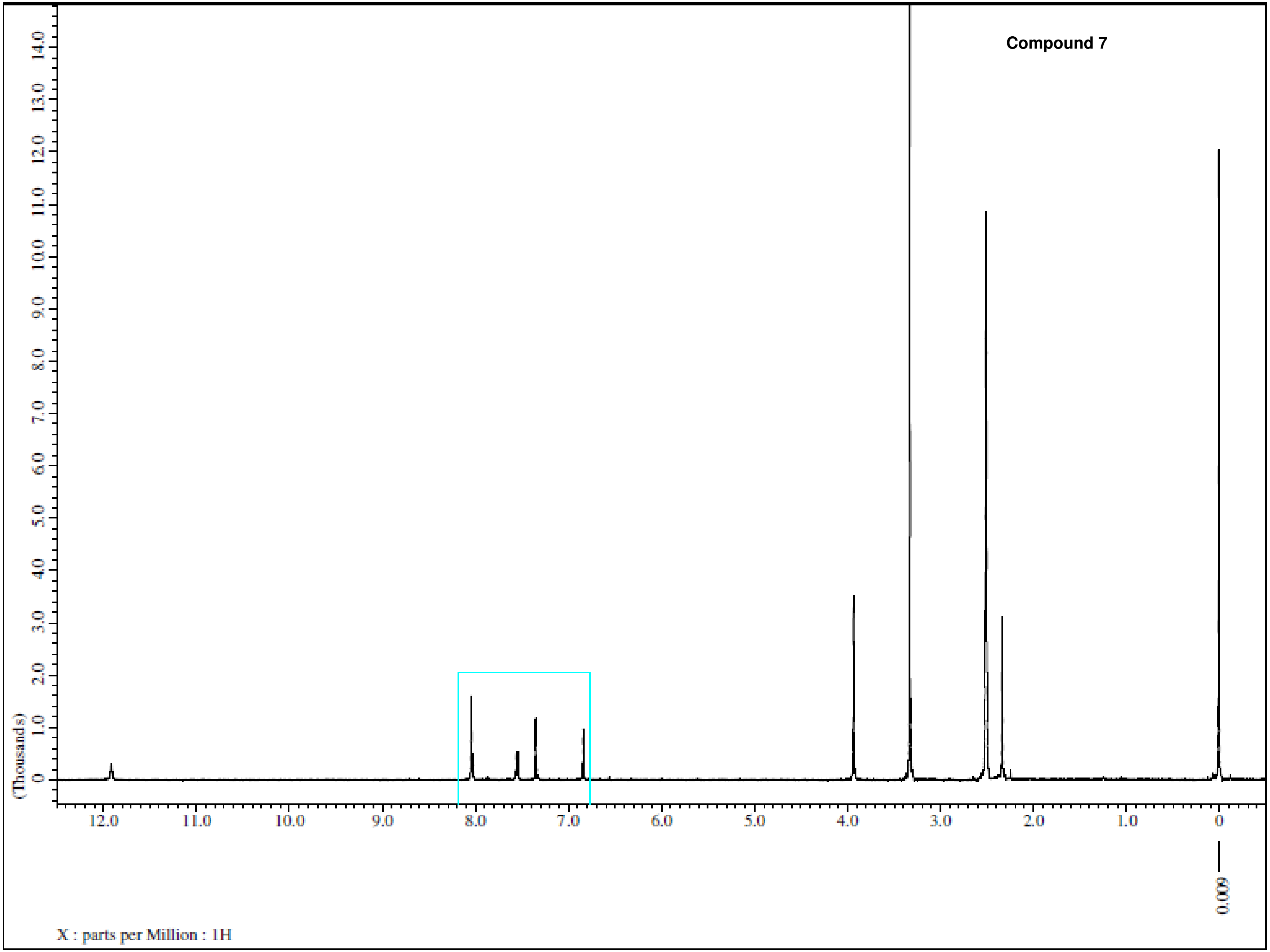

Compound 7
(Thousands)
0
1.0
2.0
3.0
4.0
5.0
6.0
7.0
8.0
9.0
10.0
11.0
12.0
13.0
14.0
12.0
11.0
10.0
9.0
8.0
7.0
6.0
5.0
4.0
3.0
2.0
1.0
0
0.009
X : parts per Million : 1H

Compound 8

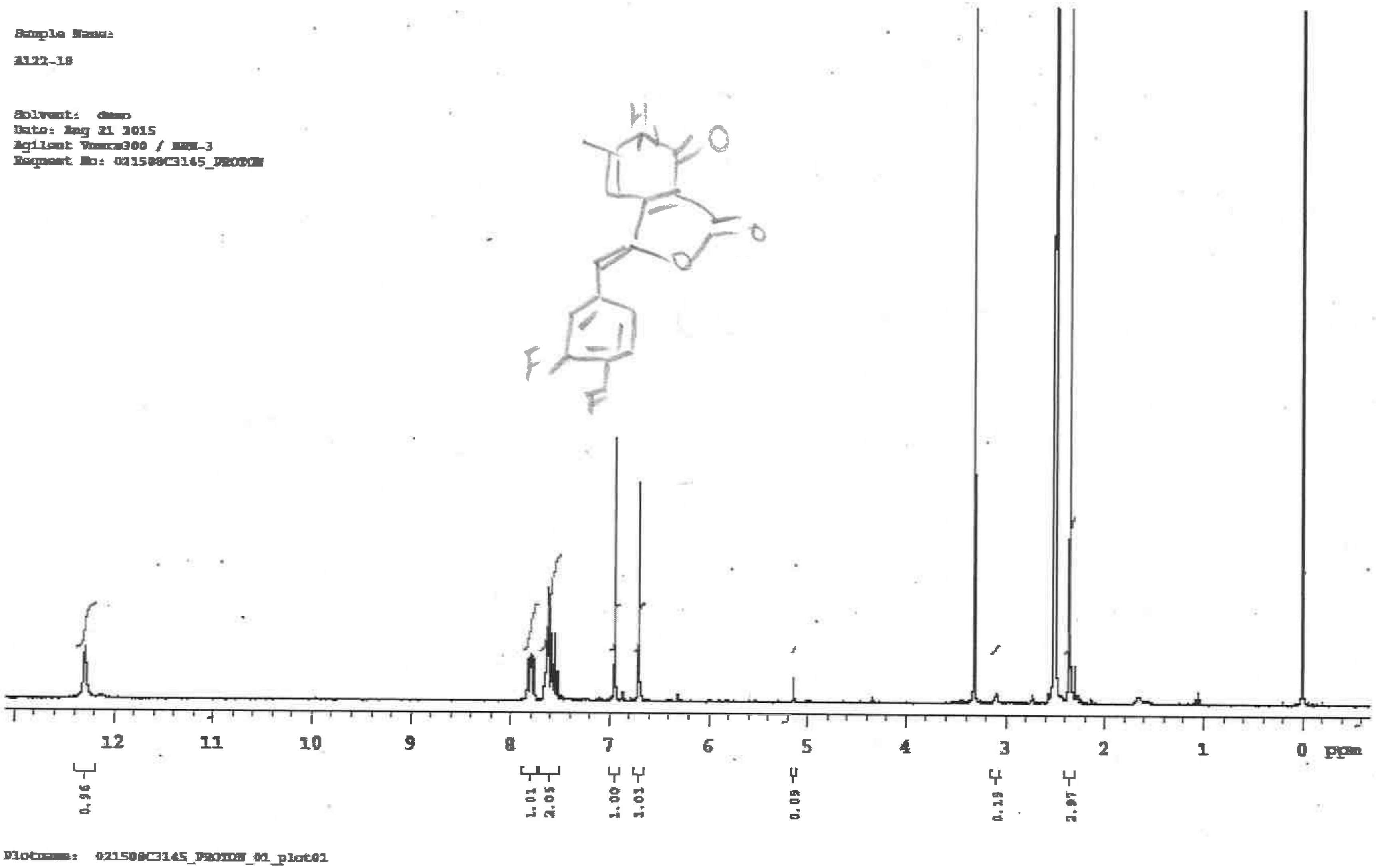
Sample Name:
A122-18
Solvent: dmso
Date: Aug 21 2015
Agilent [illegible]300 / [illegible]-3
Request No: 021508C3145_PROTON
12 11 10 9 8 7 6 5 4 3 2 1 0 ppm
0.96
1.01
2.05
1.00
1.01
0.09
0.19
2.97
Plotname: 021508C3145_PROTON_01_plot01

Compound 9

Sample Name:

[illegible]-30

Solvent: dmso
Date: Aug 21 2015
Agilent Vnmrs300 / NRM-3
Request No: 021508C3140_PROTON

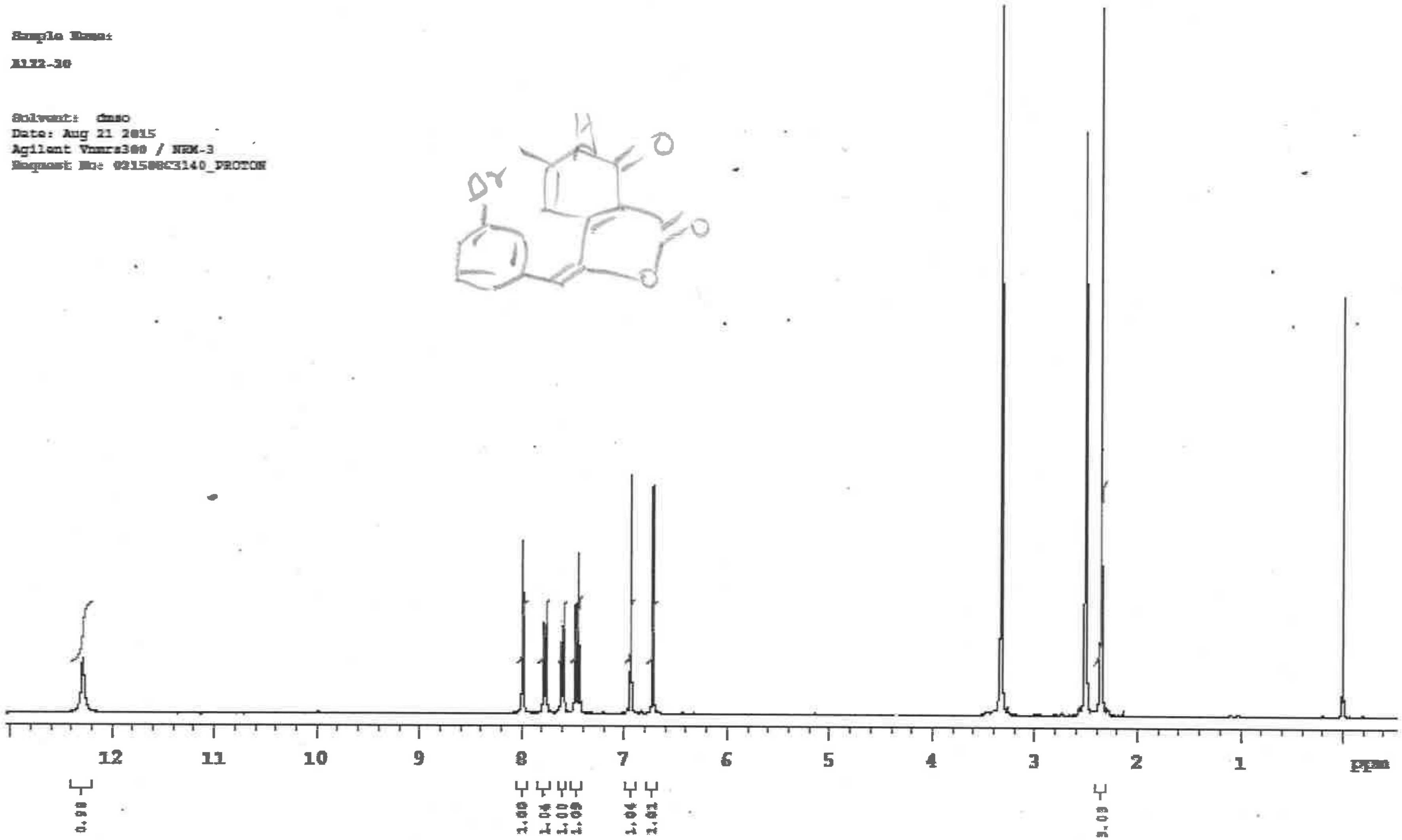

Plotname: 021508C3140_PROTON_01_plot01

Compound 10

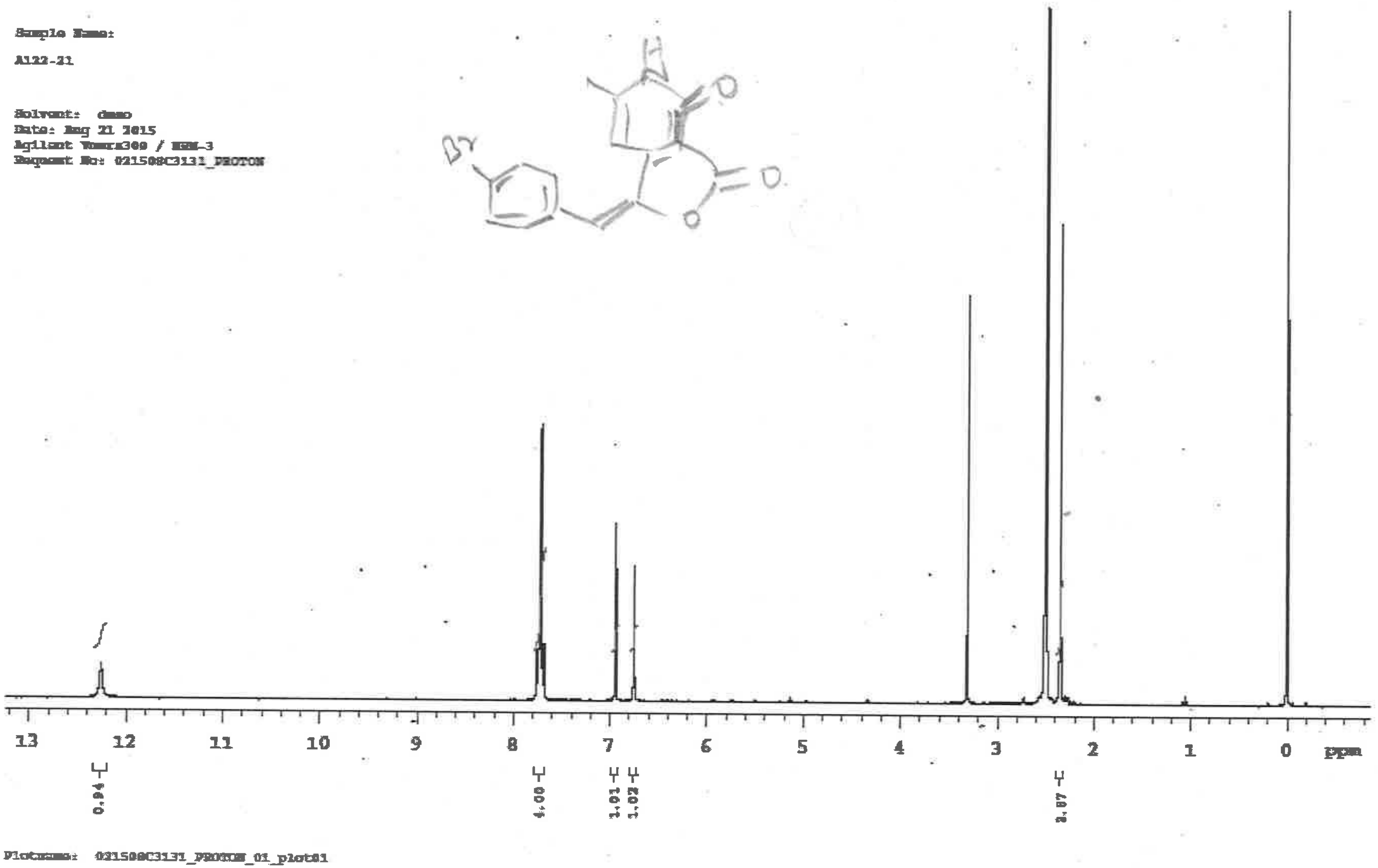
Sample Name:
A122-21
Solvent: dmso
Date: Aug 21 2015
Agilent Vnmrs300 / NMR-3
Request No: 021508C3131_PROTON
Br
O
O
O
0.94
4.00
1.01
1.02
2.87
13 12 11 10 9 8 7 6 5 4 3 2 1 0 ppm
Plotname: 021508C3131_PROTON_01_plot01

Compound 11

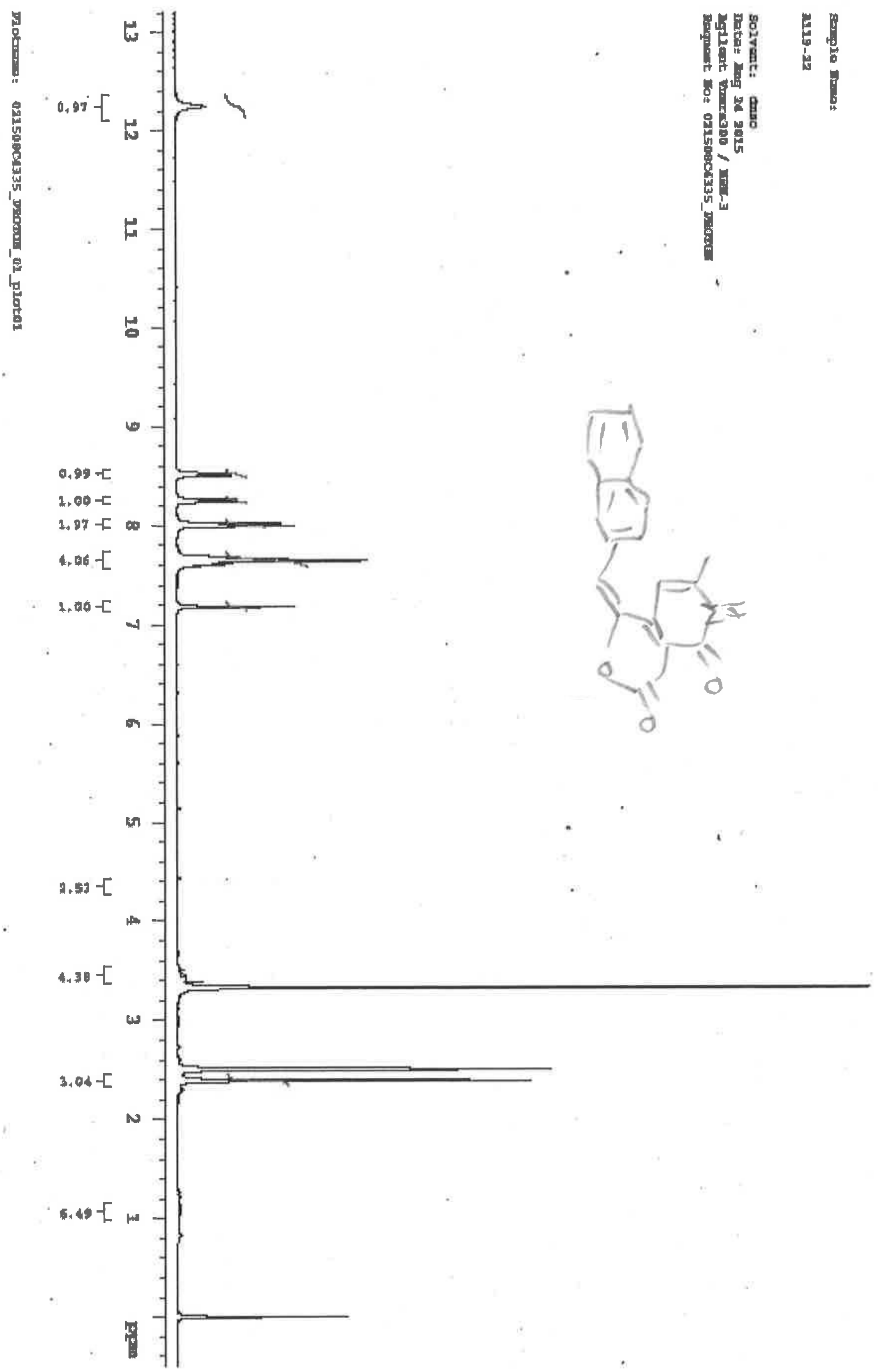

Sample Name:
Solvent:
Aug 24 2015
13
12
11
10
9
8
7
6
5
4
3
2
1
ppm
0.97
0.99
1.00
1.97
4.06
1.00
2.53
4.38
3.04
6.49

Compound 12

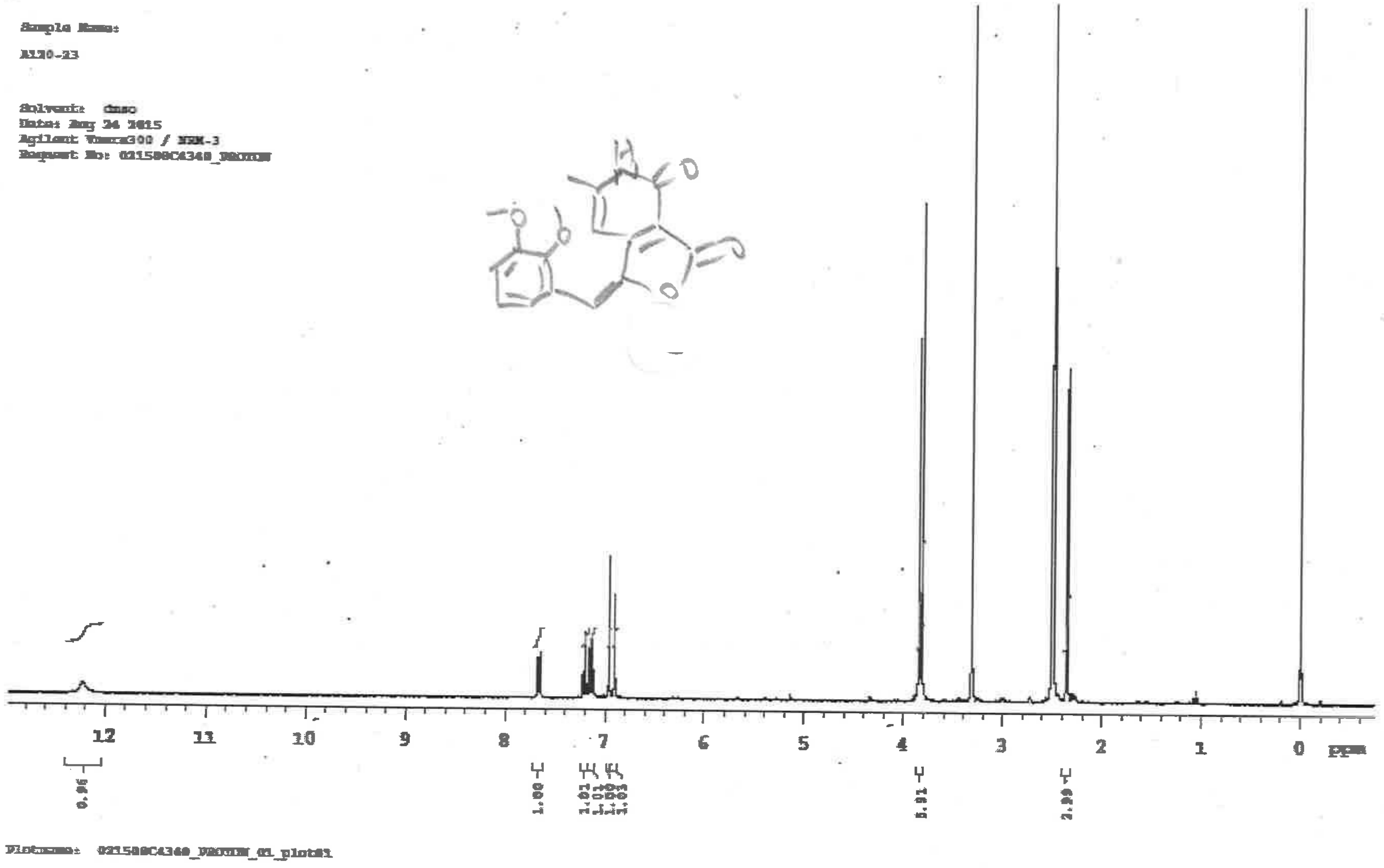
Sample Name:
A120-23
Solvent: dmso
Date: Aug 24 2015
12
11
10
9
8
7
6
5
4
3
2
1
0
ppm
1.00
5.91

Compound 13

Sample Name:

A119-27

Solvent: dmso
Date: Aug 28 2015
Agilent Vnmrs300 / NMR-3
Request No: 021508D0173_PROTON

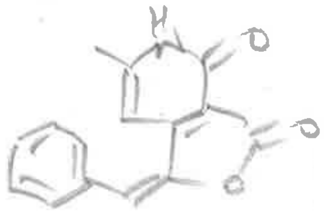

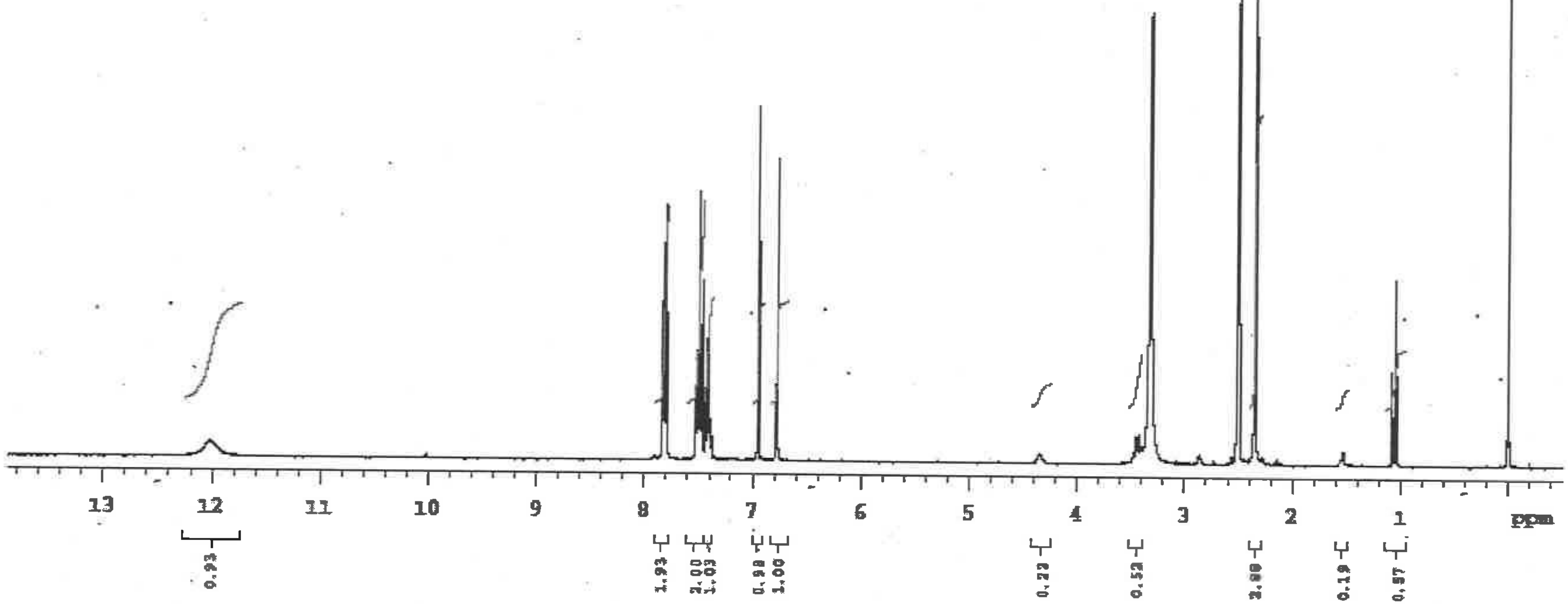

Plotname: 021508D0173_PROTON_01_plot01

Compound 14

Sample Name:

Solvent: dmso
Date: Nov 4 2015
Agilent 400-MR / NMR-3
Request No: 021511A4422_PROTON

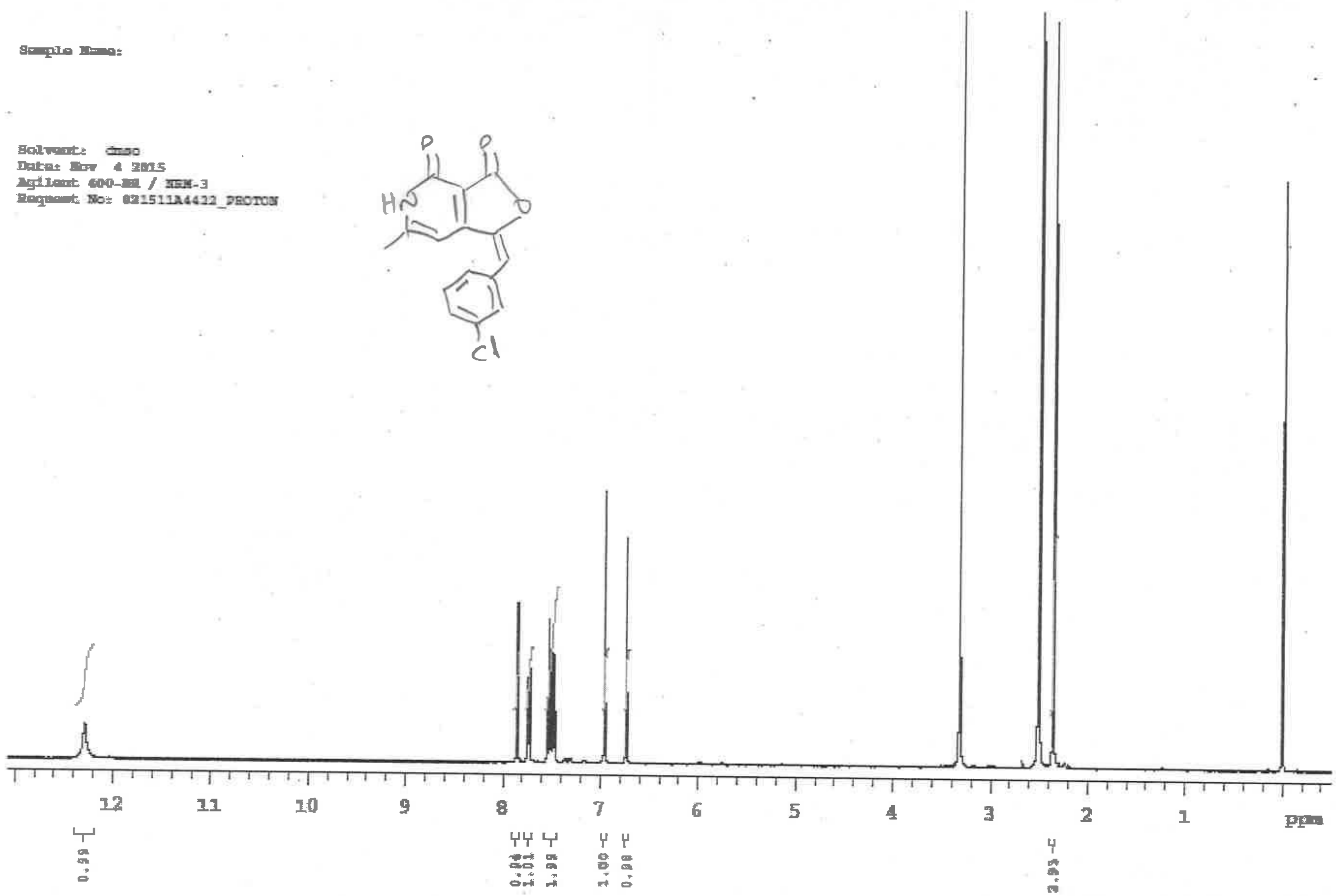

Plotname: 021511A4422_PROTON_01_plot01

Compound 15

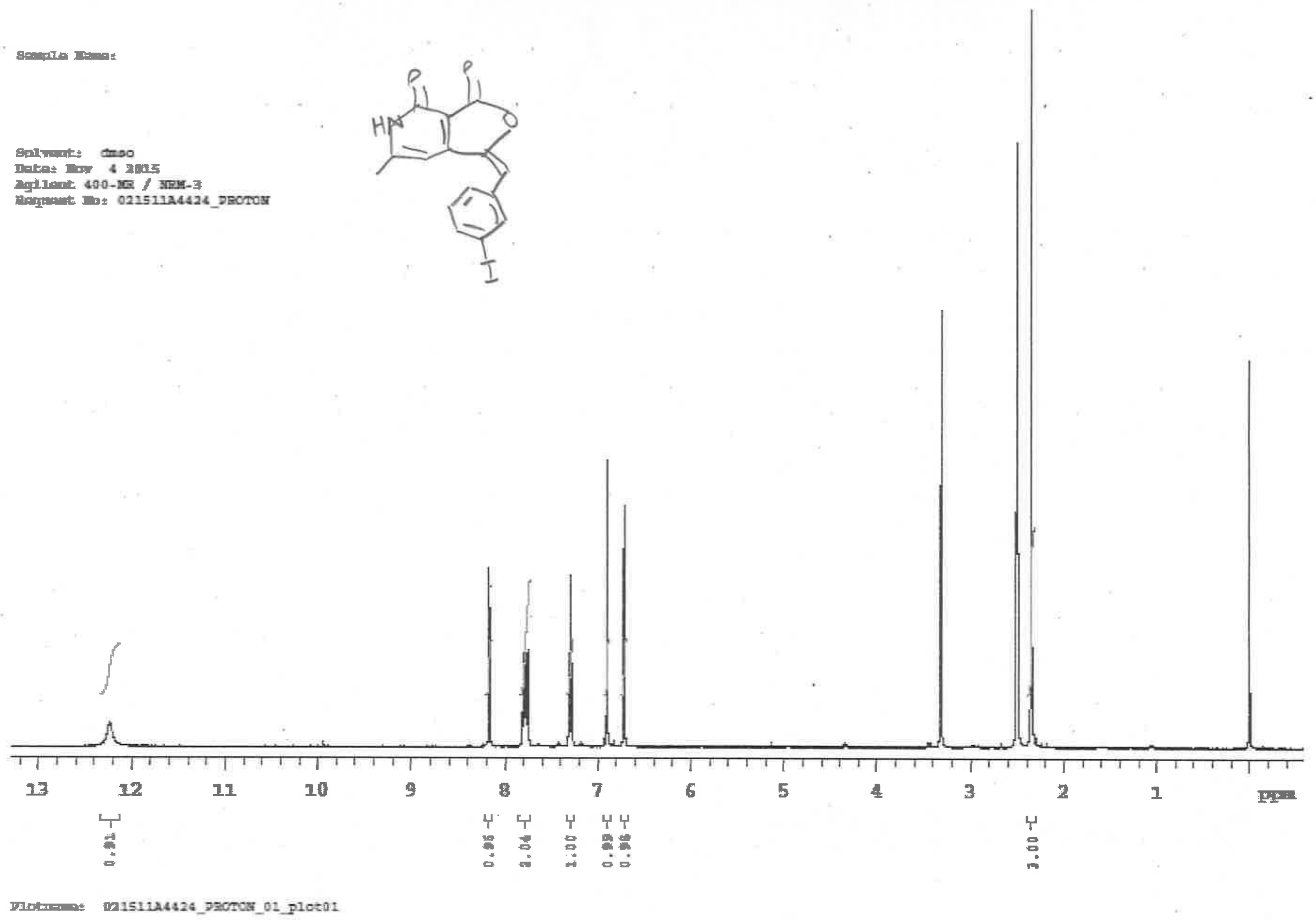
Sample Name:
Solvent: dmso
Date: Nov 4 2015
Agilent 400-MR / NRM-3
Request No: 021511A4424_PROTON
HN
O
O
O
I
13
12
11
10
9
8
7
6
5
4
3
2
1
ppm
0.91
0.96
2.04
1.00
0.98
3.00
Plotname: 021511A4424_PROTON_01_plot01

Compound 16

Sample Name:
A122-15-3

Solvent: dmso
Date: Nov 4 2015
Agilent 400-MR / NMR-3
Request No: 021511A4428_PROTON

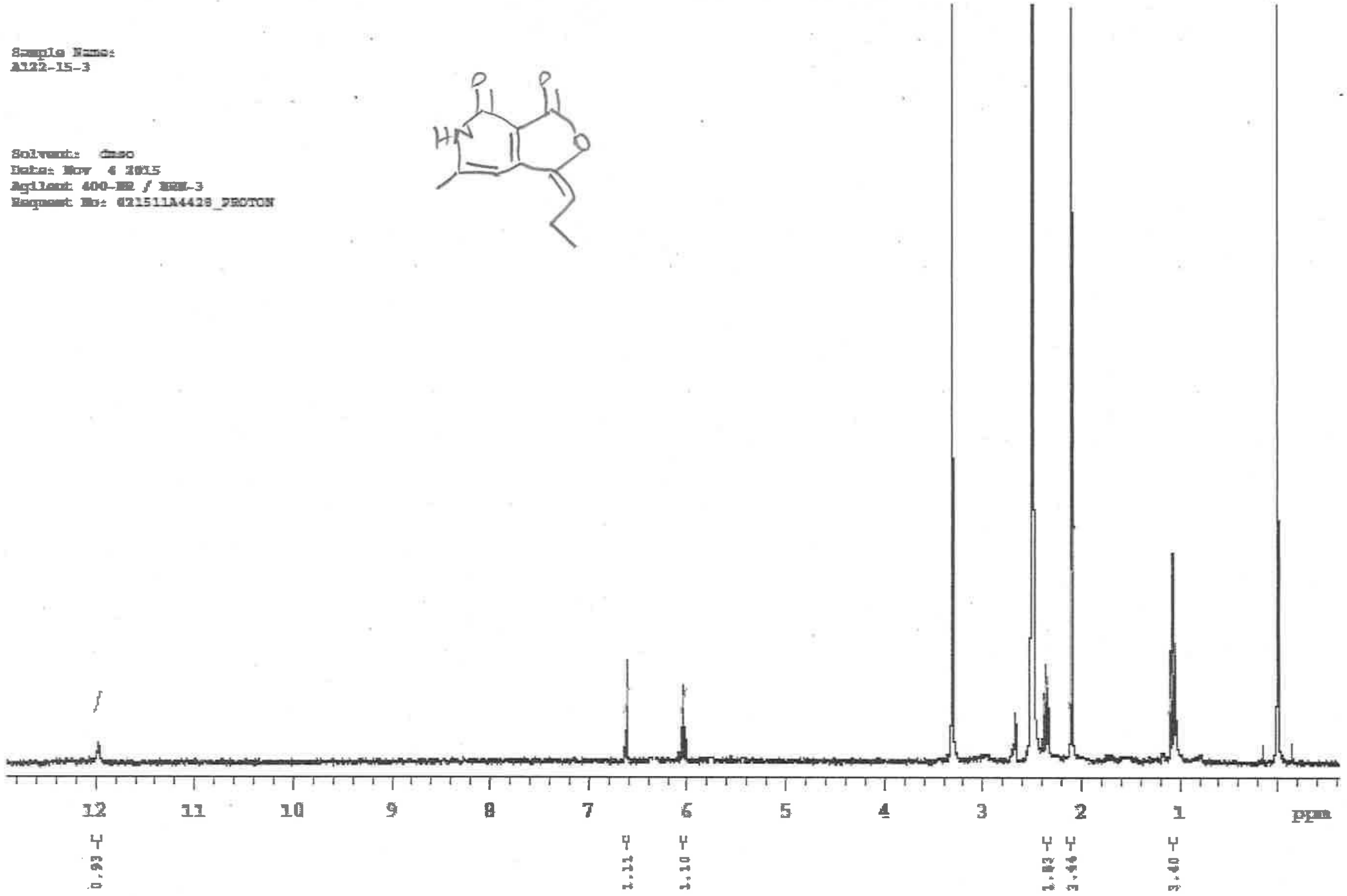

Plotname: 021511A4428_PROTON_01_plot01

Compound 17

Sample Name:

Solvent: dmso
Date: Nov 6 2015
Agilent 400-MR / NMR-3
Request No: 021511A7336_PROTON

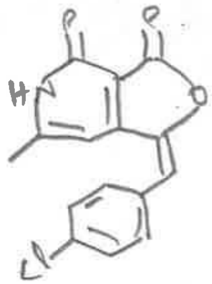

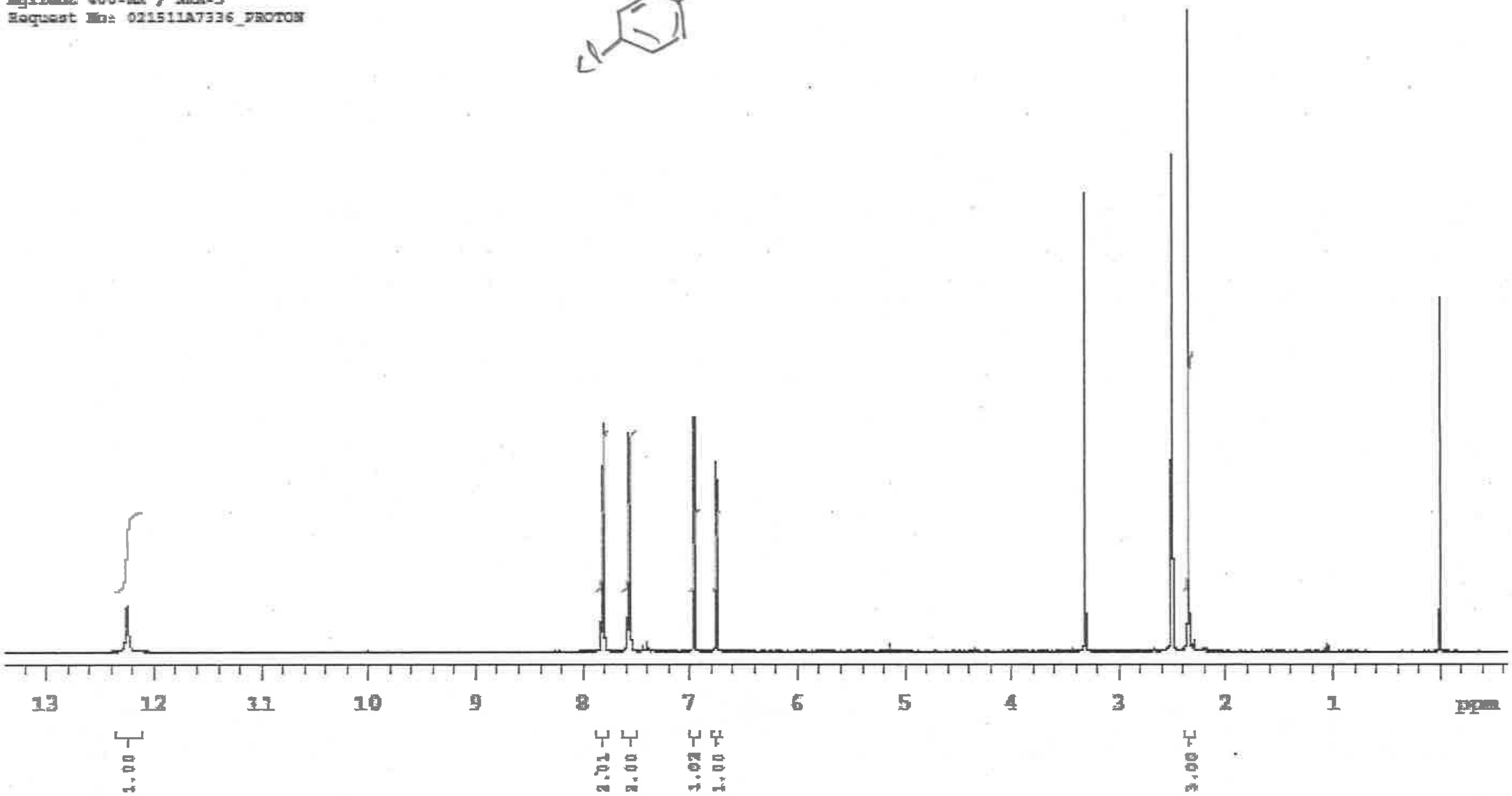

Plotname: 021511A7336_PROTON_01_plot01

Compound 18

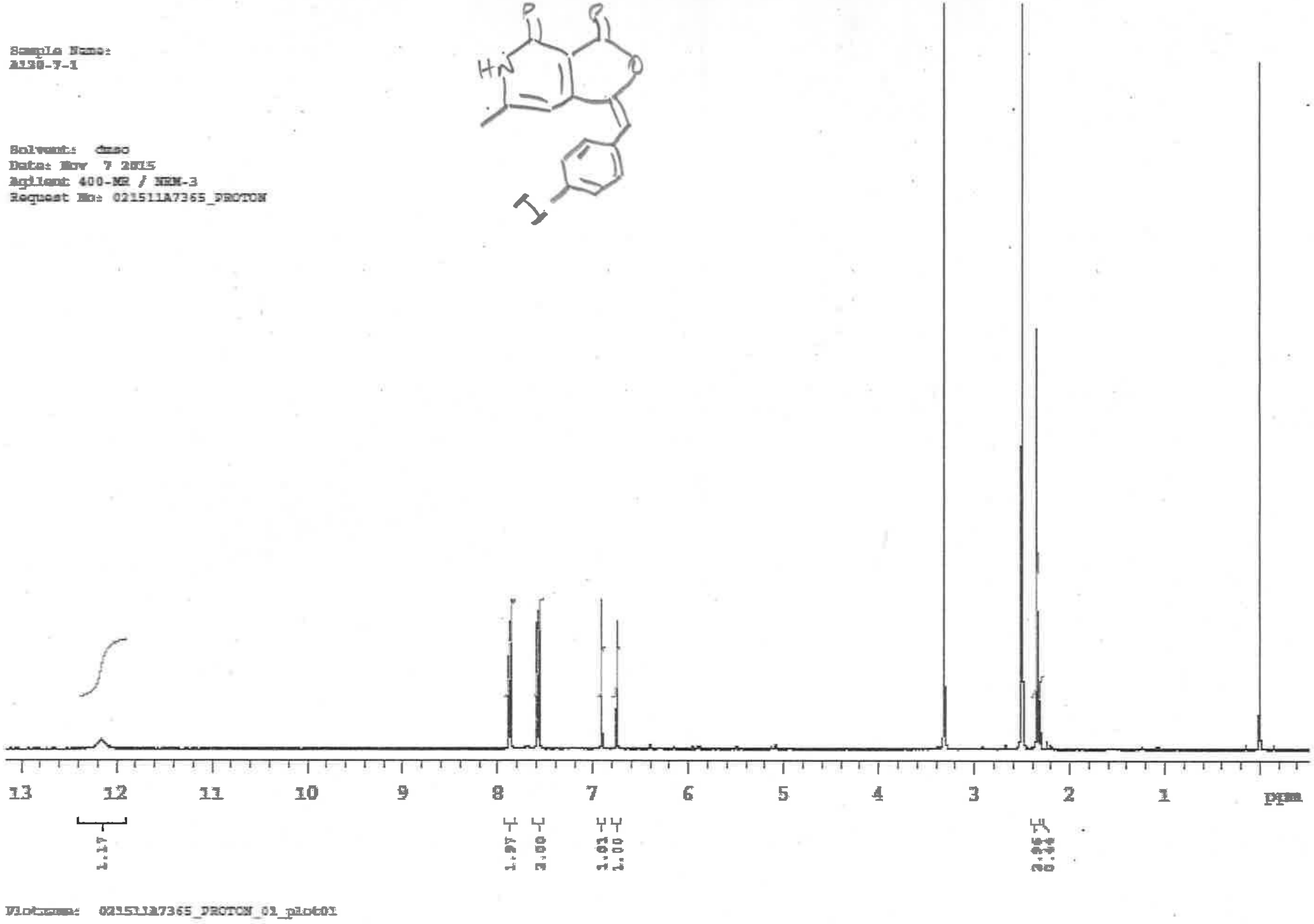

Sample Name:
Solvent: dmso
Date: Nov 7 2015
Agilent 400-MR / NRM-3
Request No: 021511A7365_PROTON
HN
I
13
12
11
10
9
8
7
6
5
4
3
2
1
ppm
1.17
1.97
2.00
1.01
1.00
Plotname: 021511A7365_PROTON_01_plot01

Compound 19

Sample Name:

Solvent: dmso
Date: Nov 6 2015
Agilent Vnmrs300 / NMR-3
Request No: 0215111a7353_PROTON

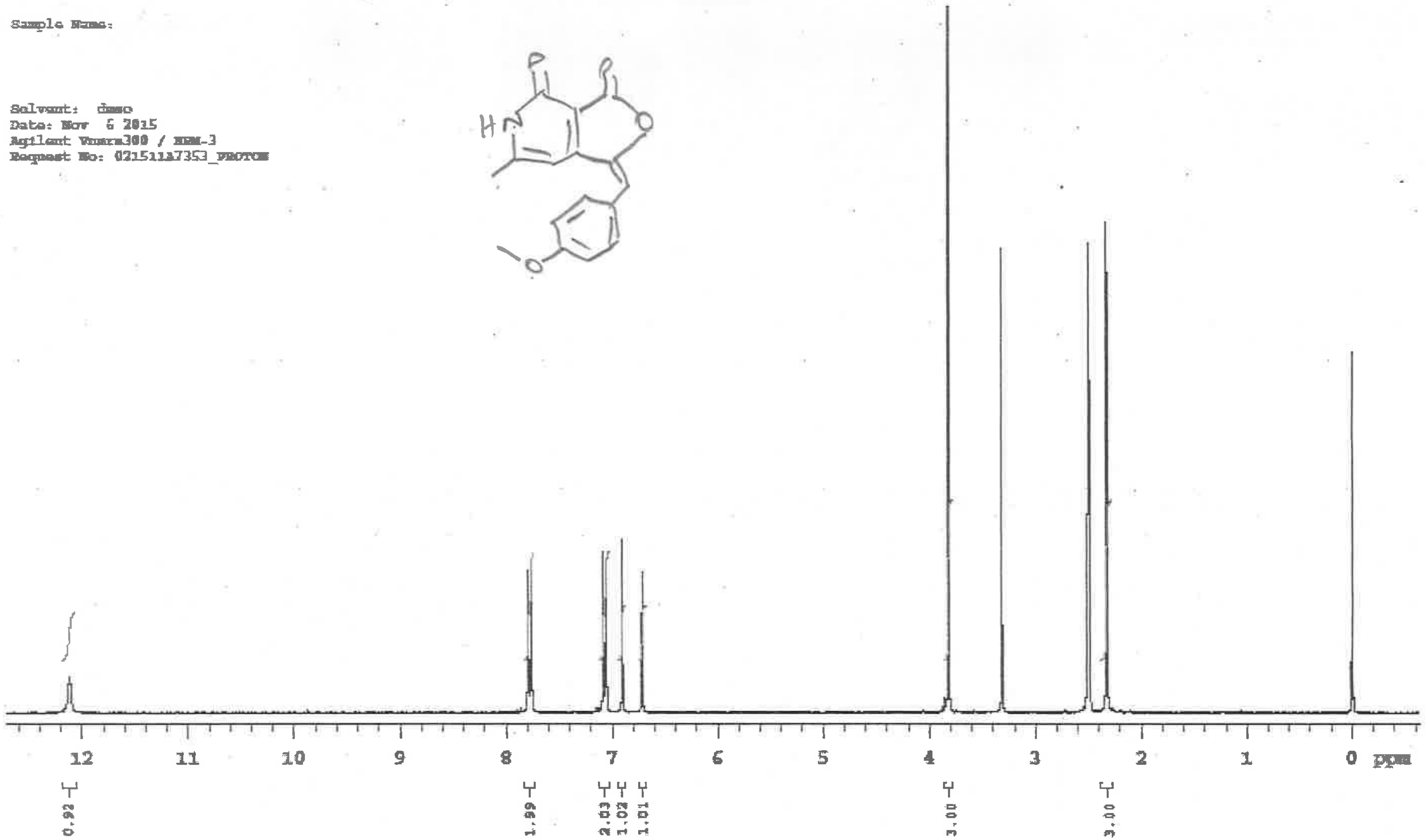

Plotname: 0215111a7353_PROTON_01_plot01

Compound 20

Sample Name:

Solvent: dmso
Date: Nov 6 2015
Agilent 400-MR / NMR-3
Request No: 021511A7352_PROTON

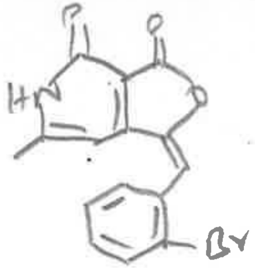

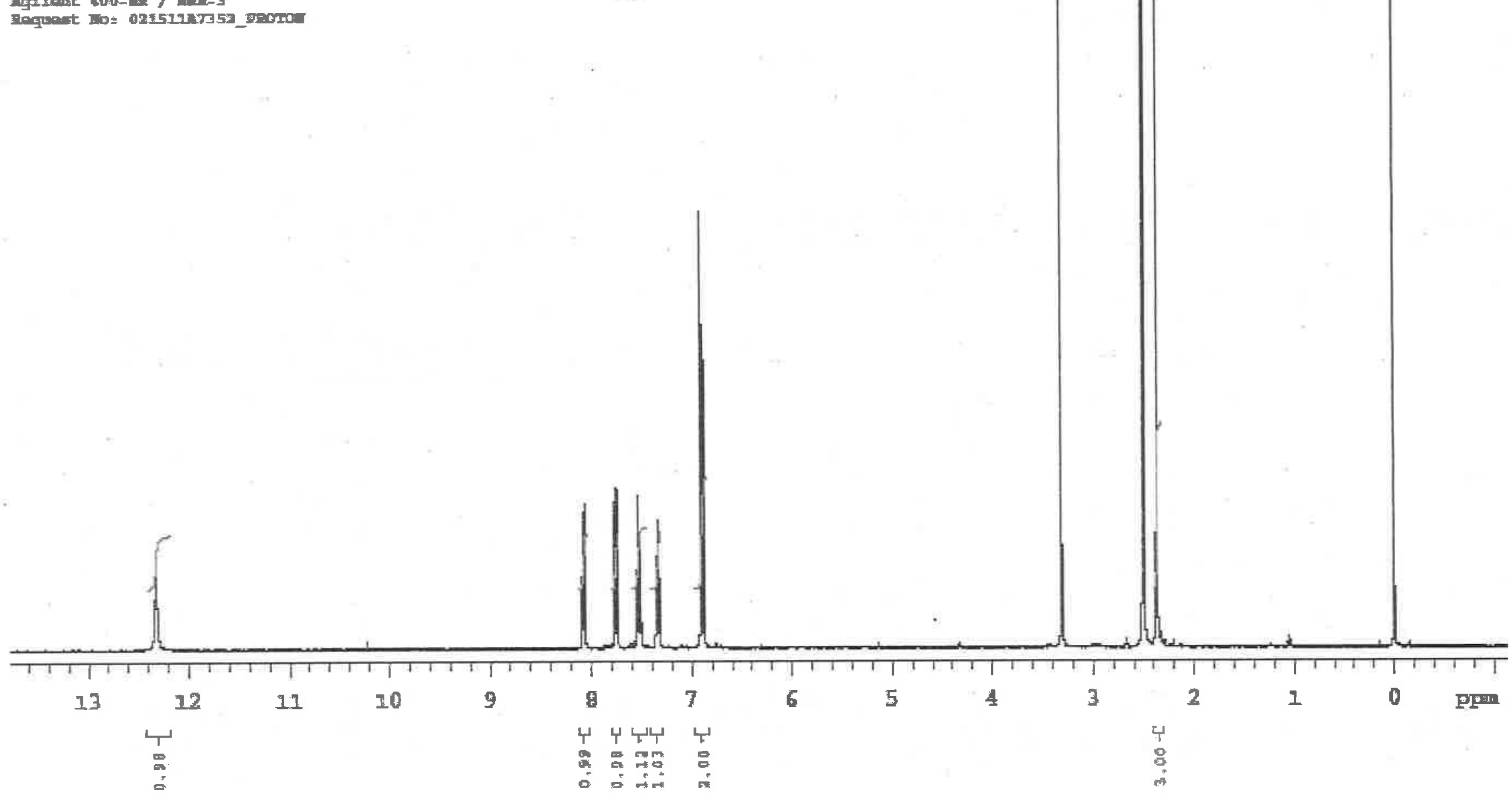

Plotname: 021511A7352_PROTON_01_plot01

Compound 21

Sample Name:
A119-16-1

Solvent: dmso
Date: Nov 6 2015
Agilent 400-MR / NRM-3
Request No: 021511A7343_PROTON

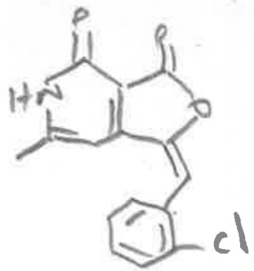

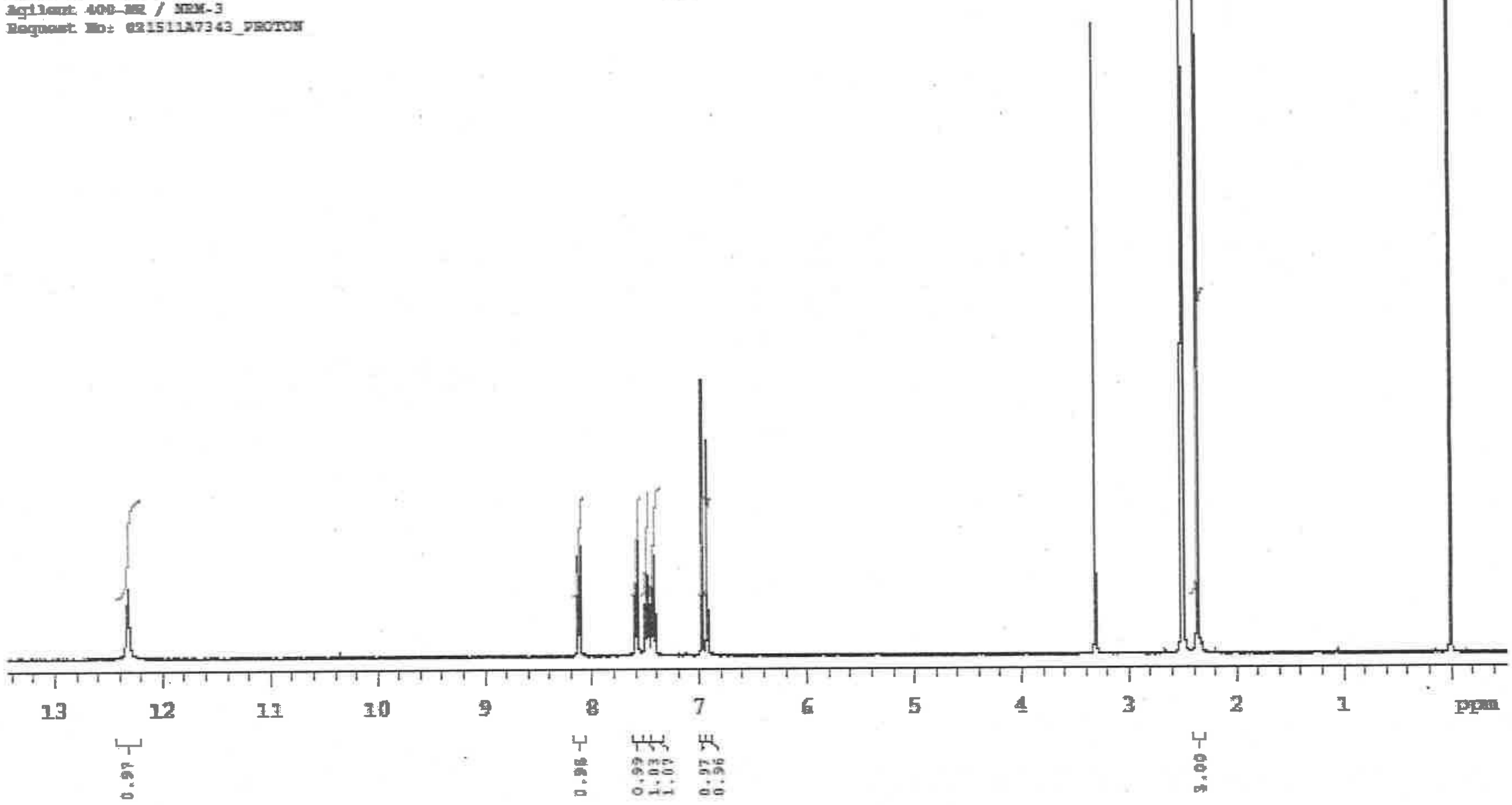

Plotname: 021511A7343_PROTON_01_plot01

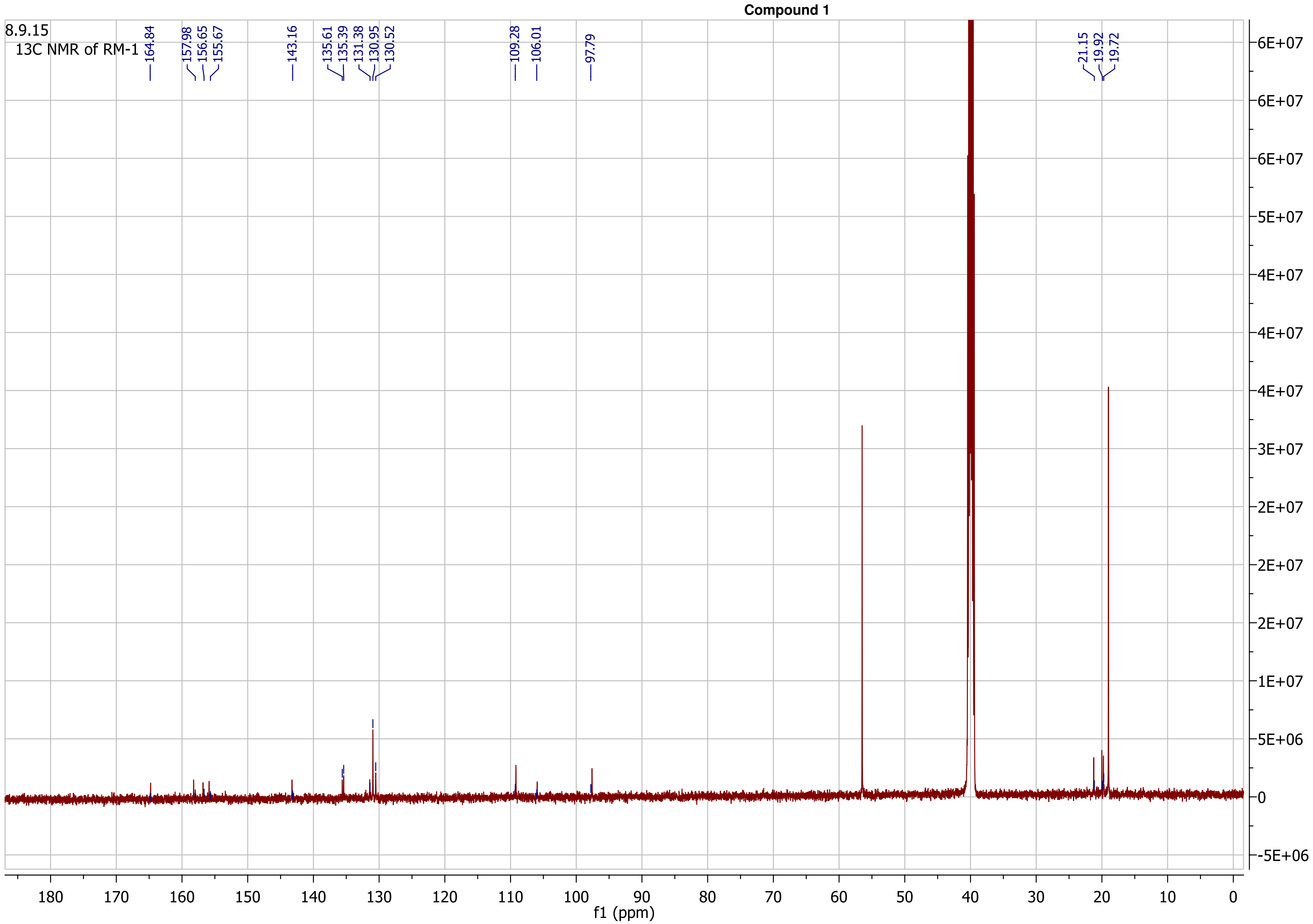
Compound 1
8.9.15
13C NMR of RM-1
164.84
157.98
156.65
155.67
143.16
135.61
135.39
131.38
130.95
130.52
109.28
106.01
97.79
21.15
19.92
19.72
f1 (ppm)

Comopund 2

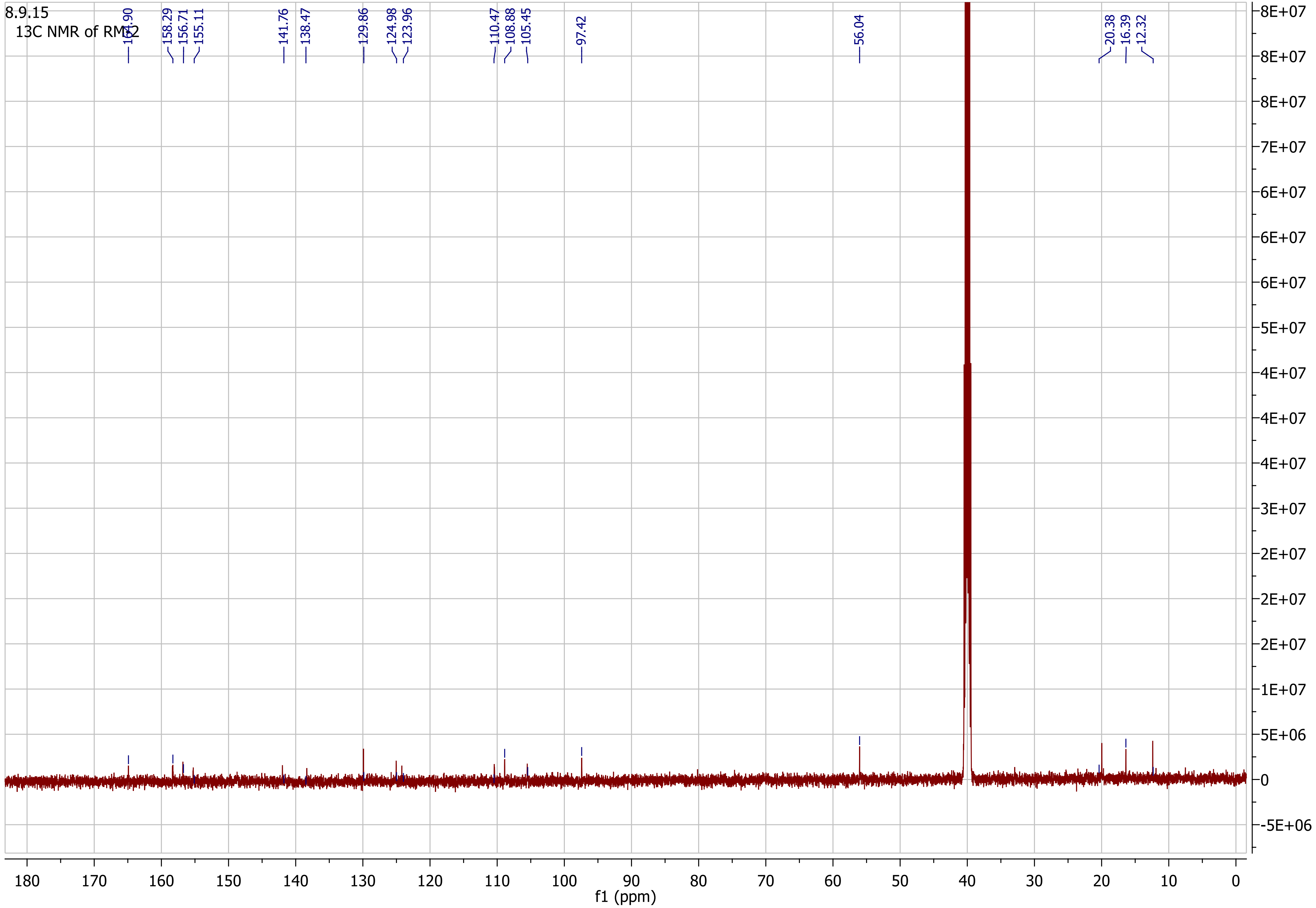
8.9.15
13C NMR of RM12
158.29
156.71
155.11
141.76
138.47
129.86
124.98
123.96
110.47
108.88
105.45
97.42
56.04
20.38
16.39
12.32
f1 (ppm)

Compound 3

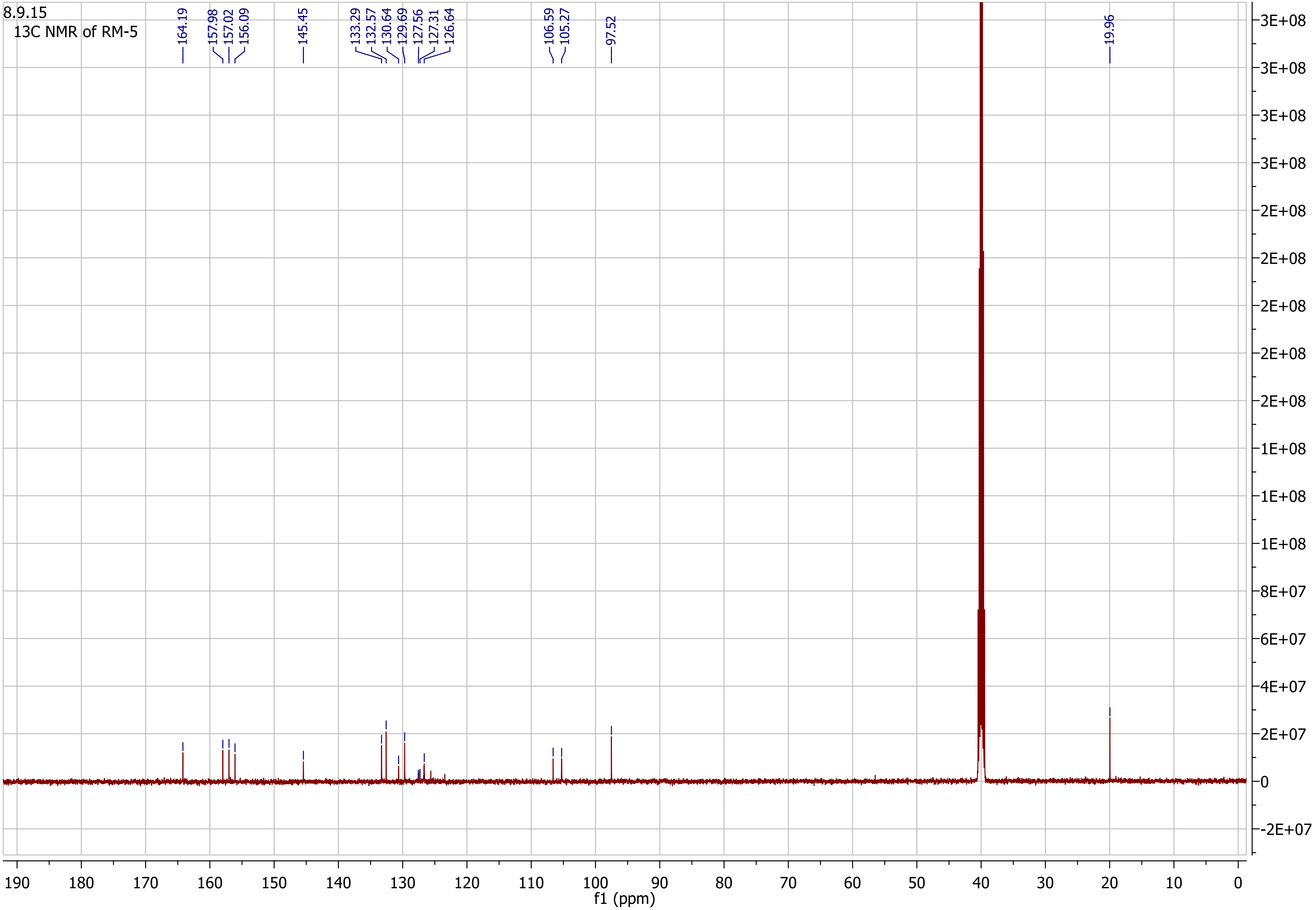
8.9.15
13C NMR of RM-5
164.19
157.98
157.02
156.09
145.45
133.29
132.57
130.64
129.69
127.56
127.31
126.64
106.59
105.27
97.52
19.96
f1 (ppm)

Compound 4

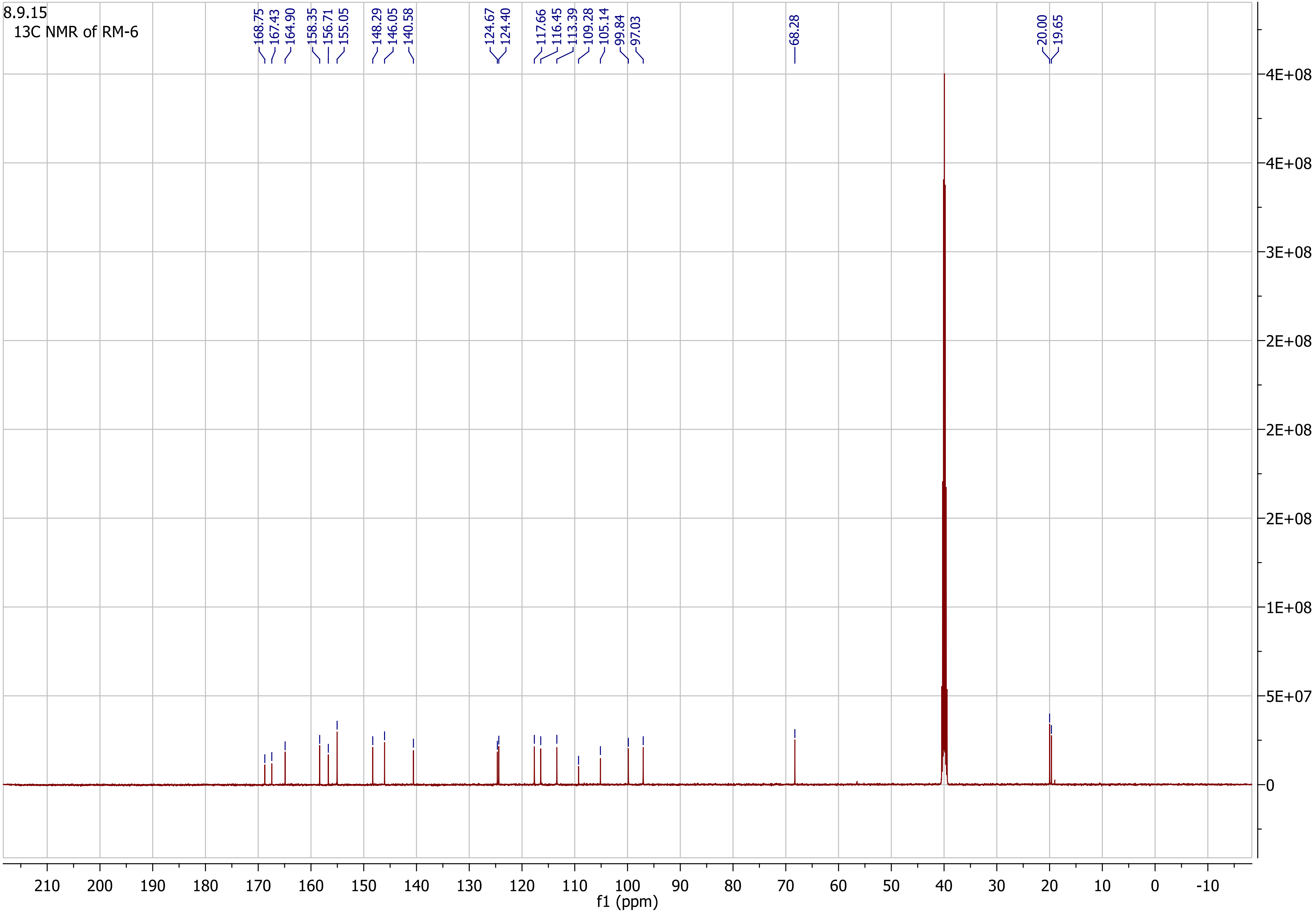

8.9.15
13C NMR of RM-6
168.75
167.43
164.90
158.35
156.71
155.05
148.29
146.05
140.58
124.67
124.40
117.66
116.45
113.39
109.28
105.14
99.84
97.03
68.28
20.00
19.65
f1 (ppm)

Compound 5

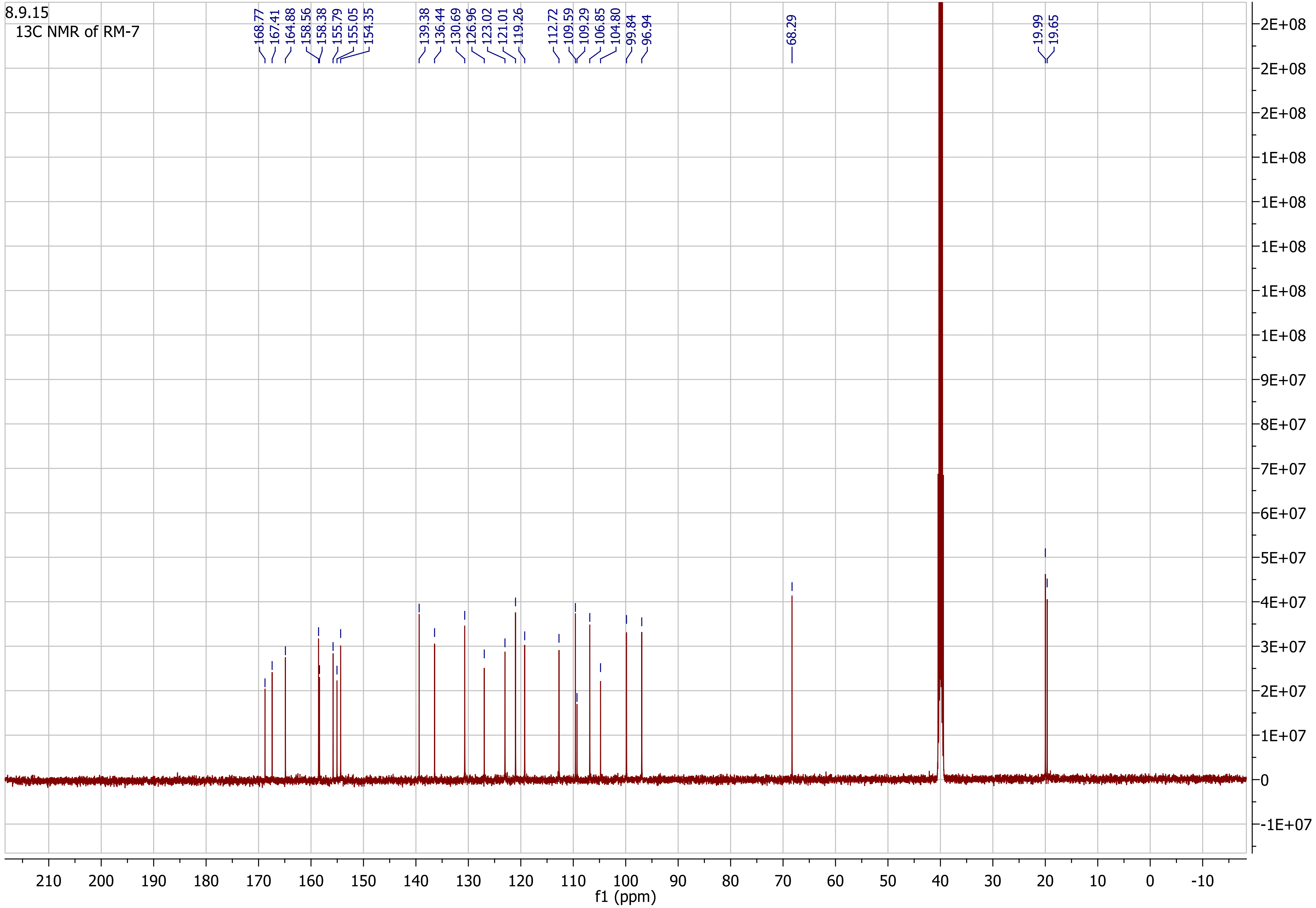

8.9.15
13C NMR of RM-7
168.77
167.41
164.88
158.56
158.38
155.79
155.05
154.35
139.38
136.44
130.69
126.96
123.02
121.01
119.26
112.72
109.59
109.29
106.85
104.80
99.84
96.94
68.29
19.99
19.65
f1 (ppm)

Compound 6

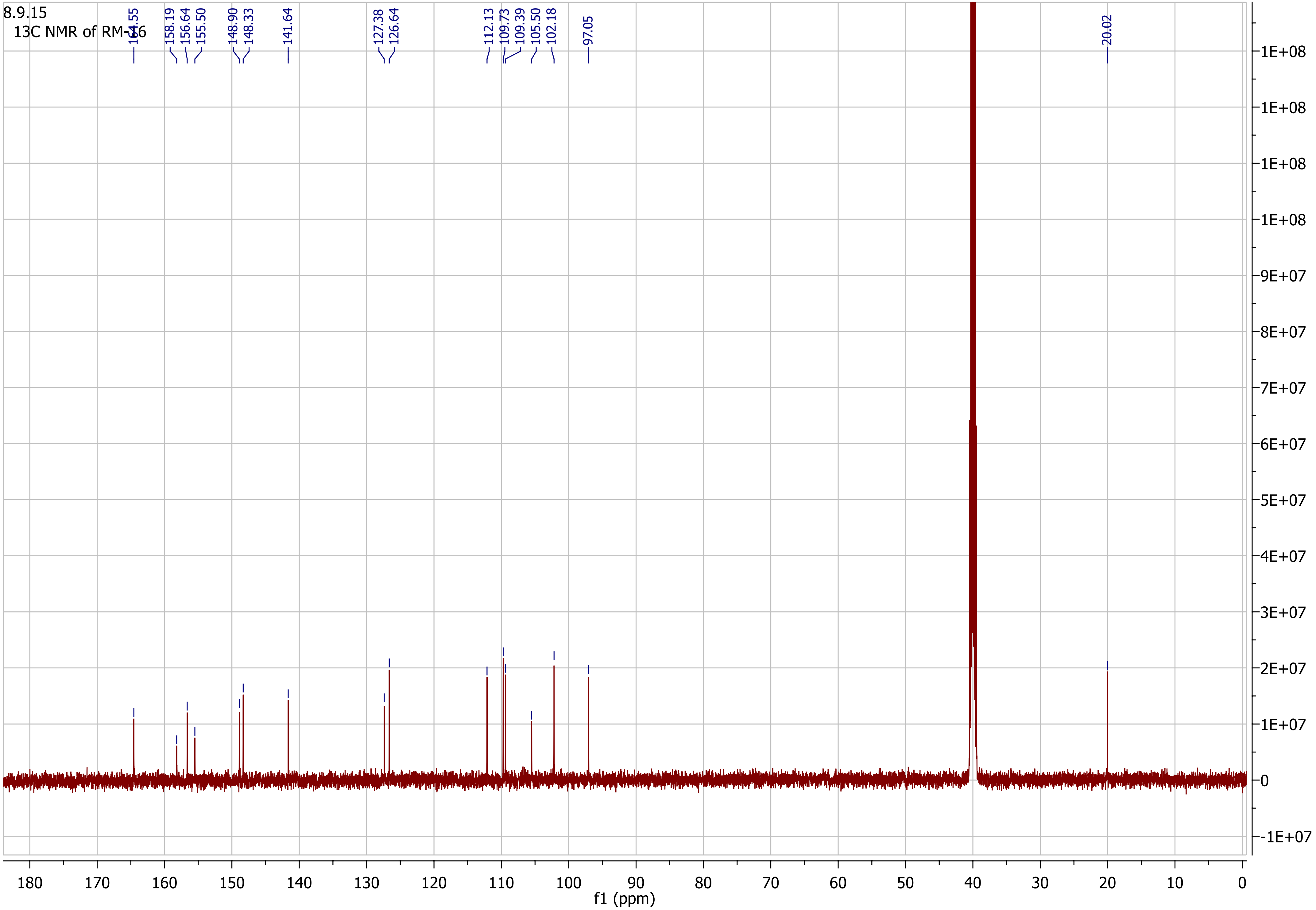

8.9.15
13C NMR of RM-66
164.55
158.19
156.64
155.50
148.90
148.33
141.64
127.38
126.64
112.13
109.73
109.39
105.50
102.18
97.05
20.02
f1 (ppm)

Compound 7

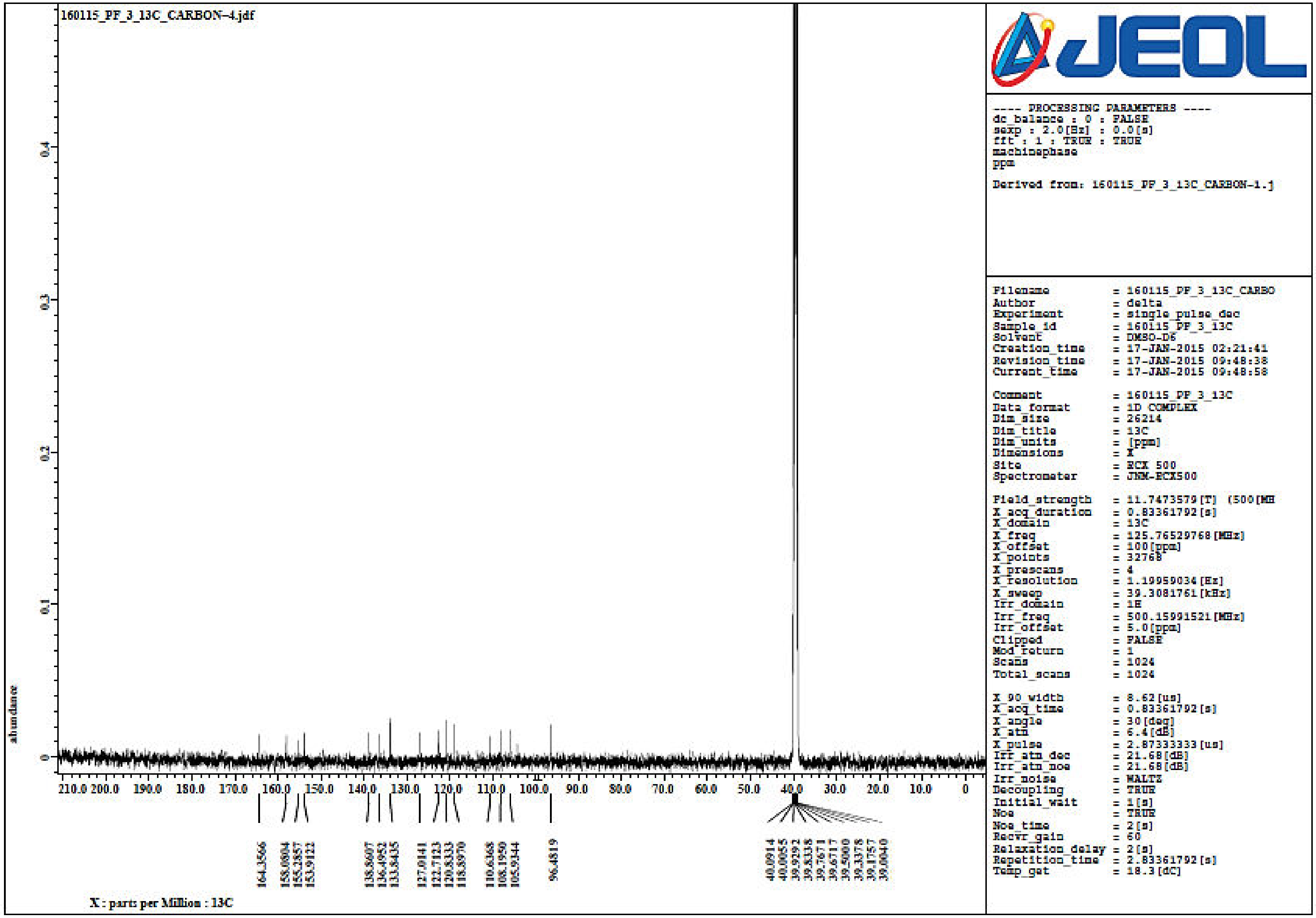

JEOL

```
---- PROCESSING PARAMETERS ----
dc_balance : 0 : FALSE
sexp : 2.0[Hz] : 0.0[s]
fft : 1 : TRUE : TRUE
machinephase
ppm

Derived from: 160115_PF_3_13C_CARBON-1.j

Filename            = 160115_PF_3_13C_CARBO
Author              = delta
Experiment          = single_pulse_dec
Sample_id           = 160115_PF_3_13C
Solvent             = DMSO-D6
Creation_time       = 17-JAN-2015 02:21:41
Revision_time       = 17-JAN-2015 09:48:38
Current_time        = 17-JAN-2015 09:48:58

Comment             = 160115_PF_3_13C
Data_format         = 1D COMPLEX
Dim_size            = 26214
Dim_title           = 13C
Dim_units           = [ppm]
Dimensions          = X
Site                = ECX 500
Spectrometer        = JNM-ECX500

Field_strength      = 11.7473579[T] (500[MH
X_acq_duration      = 0.83361792[s]
X_domain            = 13C
X_freq              = 125.76529768[MHz]
X_offset            = 100[ppm]
X_points            = 32768
X_prescans          = 4
X_resolution        = 1.19959034[Hz]
X_sweep             = 39.3081761[kHz]
Irr_domain          = 1H
Irr_freq            = 500.15991521[MHz]
Irr_offset          = 5.0[ppm]
Clipped             = FALSE
Mod_return          = 1
Scans               = 1024
Total_scans         = 1024

X_90_width          = 8.62[us]
X_acq_time          = 0.83361792[s]
X_angle             = 30[deg]
X_atn               = 6.4[dB]
X_pulse             = 2.87333333[us]
Irr_atn_dec         = 21.68[dB]
Irr_atn_noe         = 21.68[dB]
Irr_noise           = WALTZ
Decoupling          = TRUE
Initial_wait        = 1[s]
Noe                 = TRUE
Noe_time            = 2[s]
Recvr_gain          = 60
Relaxation_delay    = 2[s]
Repetition_time     = 2.83361792[s]
Temp_get            = 18.3[dC]
```

Compound 8

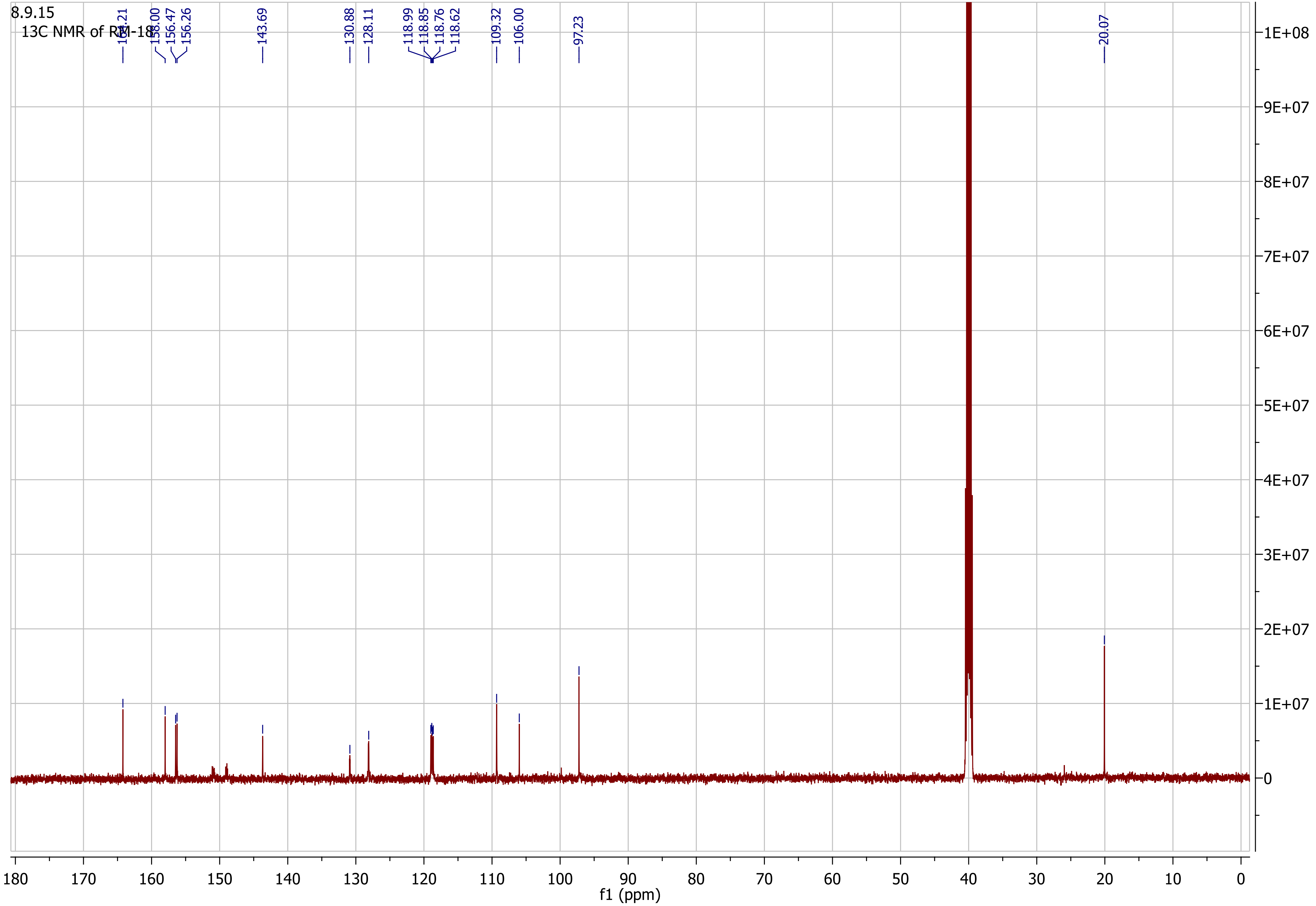
8.9.15
13C NMR of RM-18
164.21
158.00
156.47
156.26
143.69
130.88
128.11
118.99
118.85
118.76
118.62
109.32
106.00
97.23
20.07
1E+08
9E+07
8E+07
7E+07
6E+07
5E+07
4E+07
3E+07
2E+07
1E+07
0
180
170
160
150
140
130
120
110
100
90
80
70
60
50
40
30
20
10
0
f1 (ppm)

Compound 9

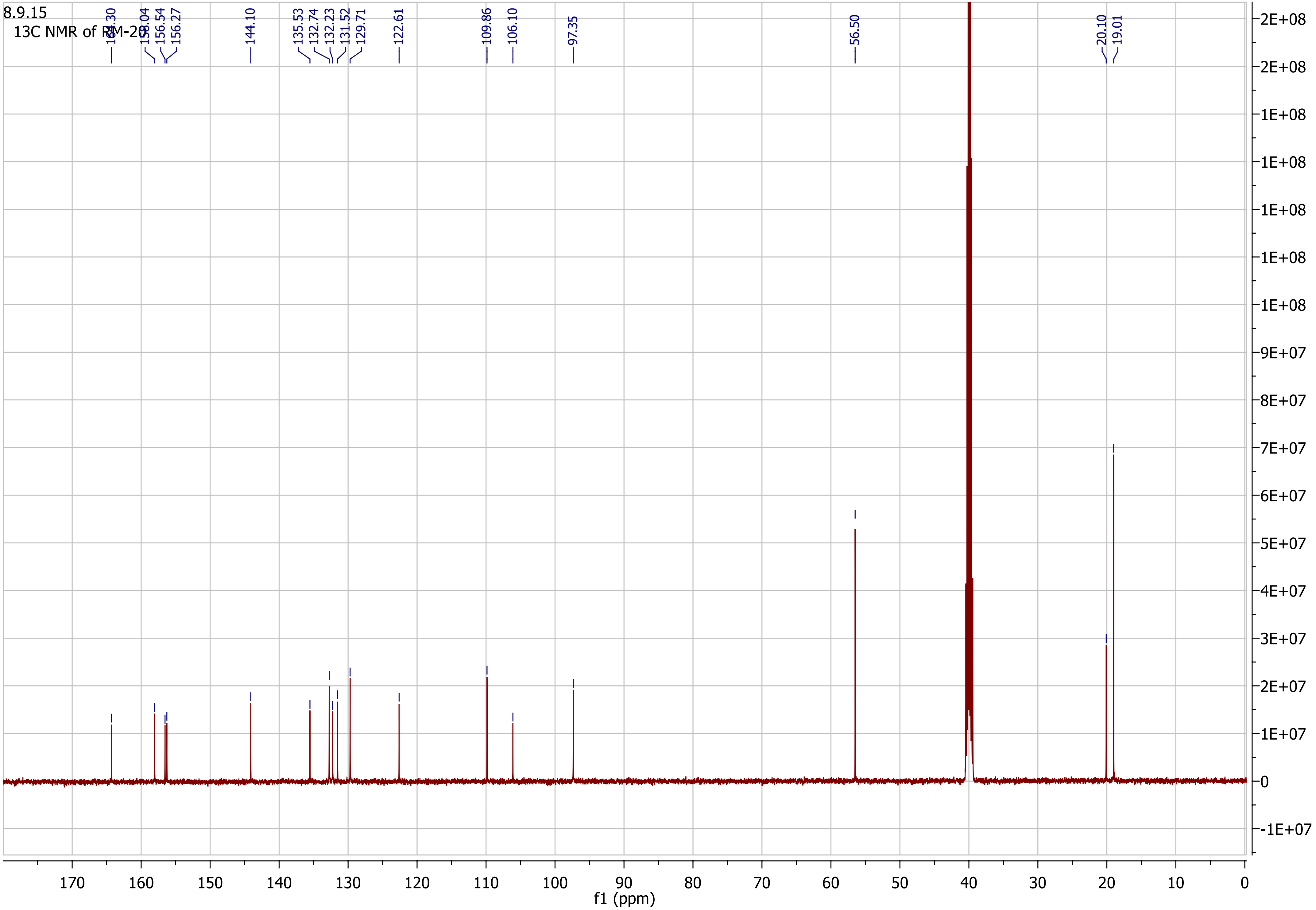
8.9.15
13C NMR of RM-20
164.30
158.04
156.54
156.27
144.10
135.53
132.74
132.23
131.52
129.71
122.61
109.86
106.10
97.35
56.50
20.10
19.01
2E+08
2E+08
1E+08
1E+08
1E+08
1E+08
1E+08
9E+07
8E+07
7E+07
6E+07
5E+07
4E+07
3E+07
2E+07
1E+07
0
-1E+07
170 160 150 140 130 120 110 100 90 80 70 60 50 40 30 20 10 0
f1 (ppm)

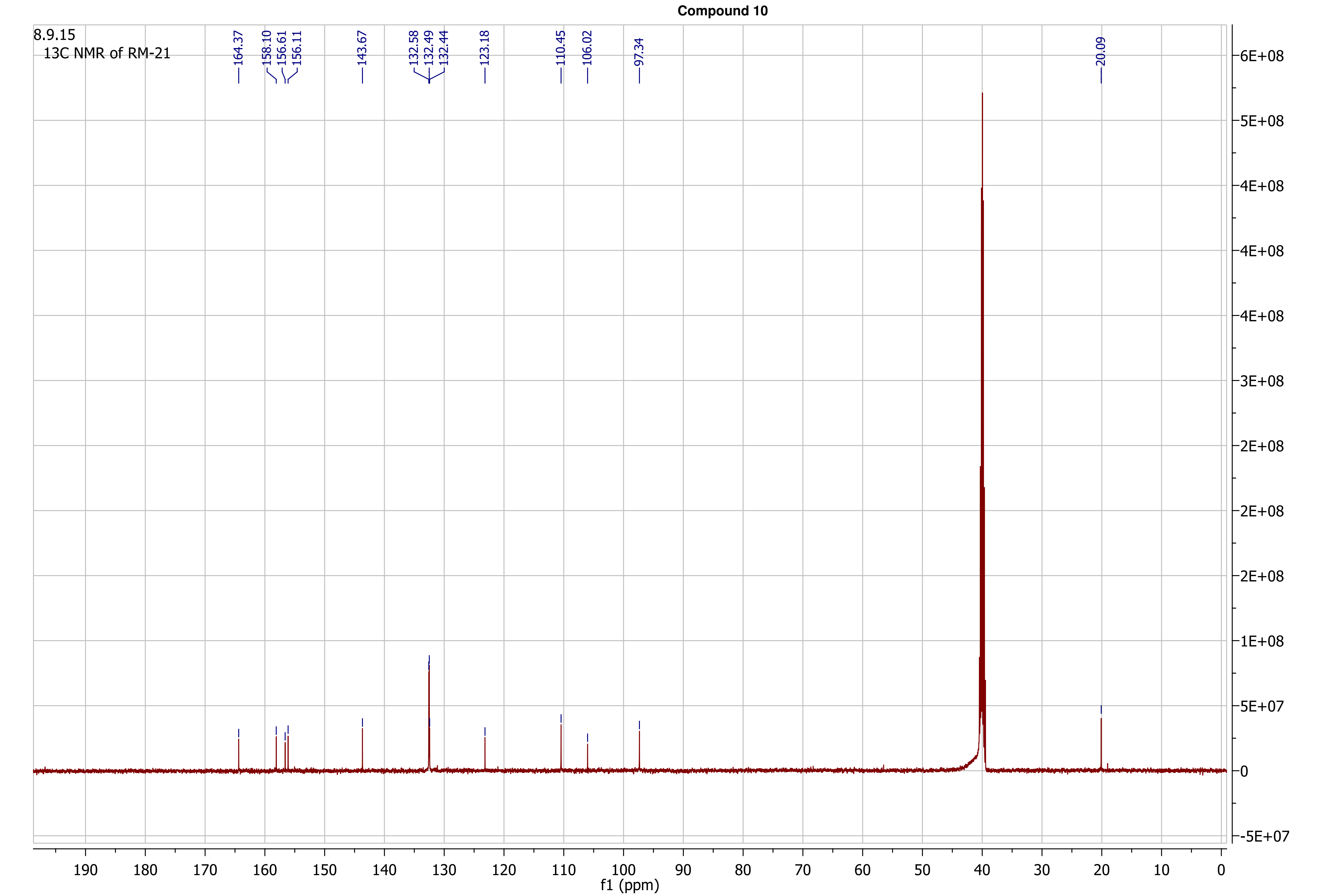

Compound 10
8.9.15
13C NMR of RM-21
164.37
158.10
156.61
156.11
143.67
132.58
132.49
132.44
123.18
110.45
106.02
97.34
20.09
f1 (ppm)

Compound 11

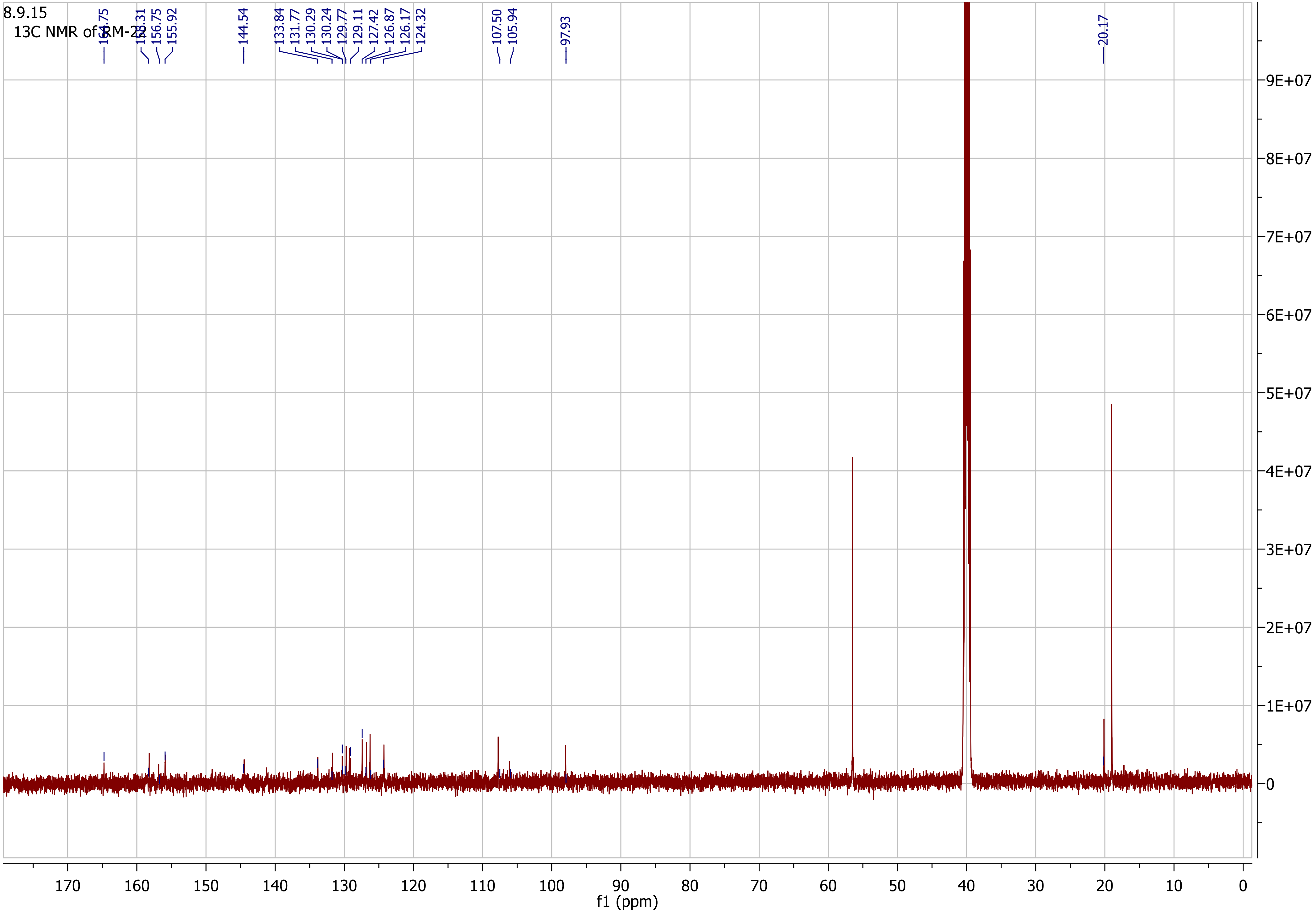
8.9.15
13C NMR of RM-22
164.75
158.31
156.75
155.92
144.54
133.84
131.77
130.29
130.24
129.77
129.11
127.42
126.87
126.17
124.32
107.50
105.94
97.93
20.17
9E+07
8E+07
7E+07
6E+07
5E+07
4E+07
3E+07
2E+07
1E+07
0
170
160
150
140
130
120
110
100
90
80
70
60
50
40
30
20
10
0
f1 (ppm)

Compound 13

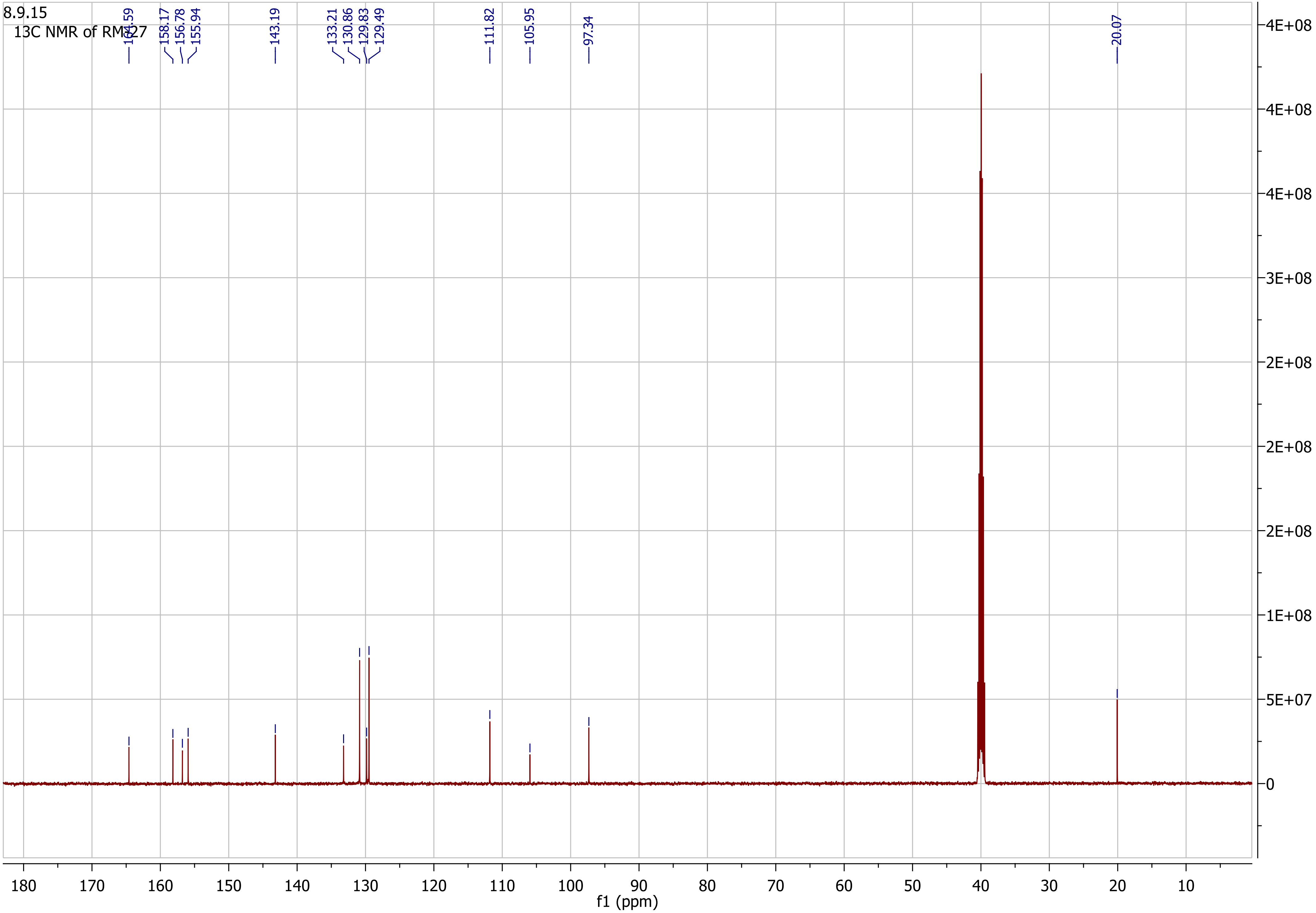
8.9.15
13C NMR of RM-27
164.59
158.17
156.78
155.94
143.19
133.21
130.86
129.83
129.49
111.82
105.95
97.34
20.07
4E+08
4E+08
4E+08
3E+08
2E+08
2E+08
2E+08
1E+08
5E+07
0
180
170
160
150
140
130
120
110
100
90
80
70
60
50
40
30
20
10
f1 (ppm)

Compound 14

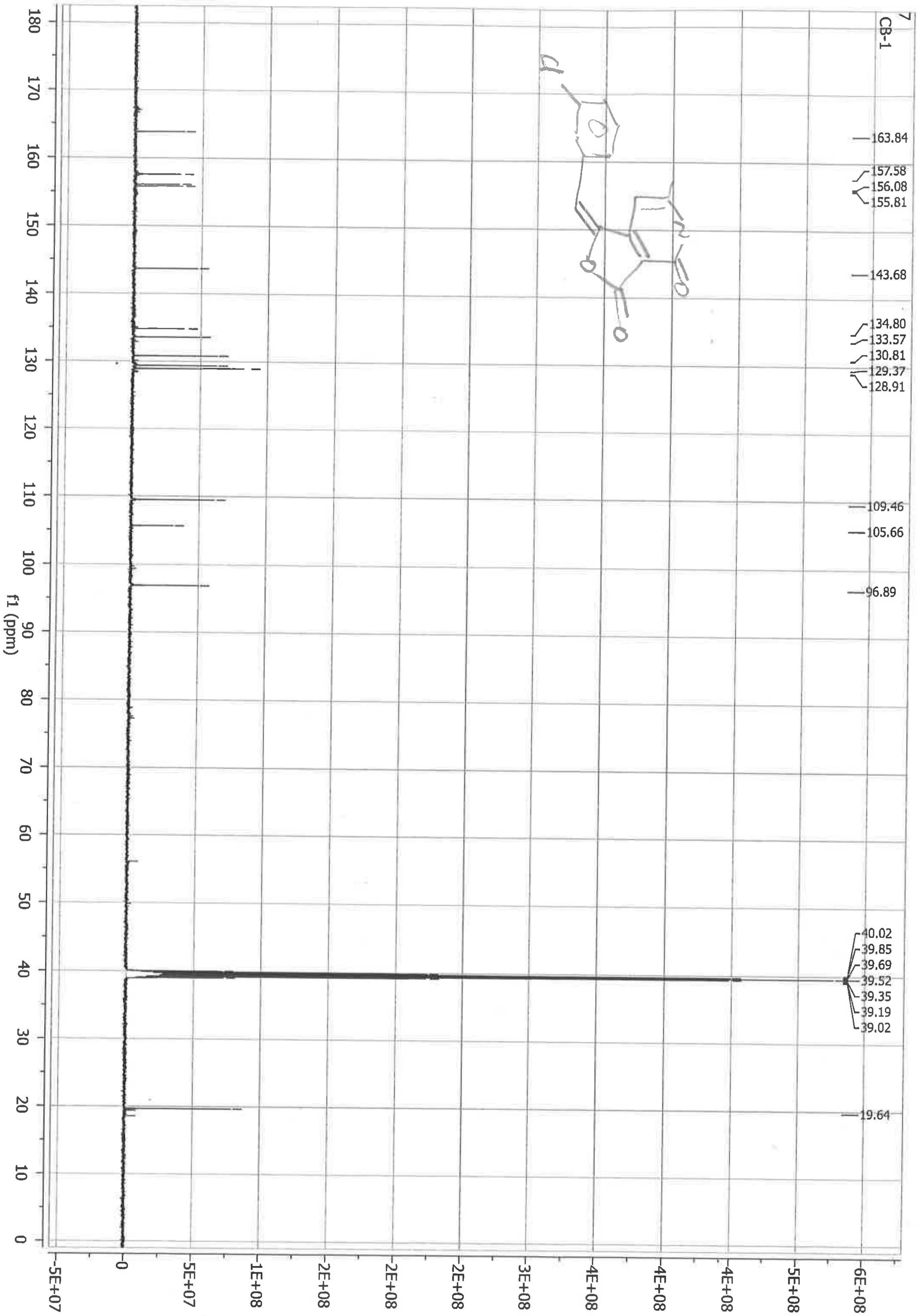
CB-1
163.84
157.58
156.08
155.81
143.68
134.80
133.57
130.81
129.37
128.91
109.46
105.66
96.89
40.02
39.85
39.69
39.52
39.35
39.19
39.02
19.64
f1 (ppm)
180
170
160
150
140
130
120
110
100
90
80
70
60
50
40
30
20
10
0
-5E+07
0
5E+07
1E+08
2E+08
2E+08
2E+08
3E+08
4E+08
4E+08
4E+08
5E+08
6E+08

Compound 15

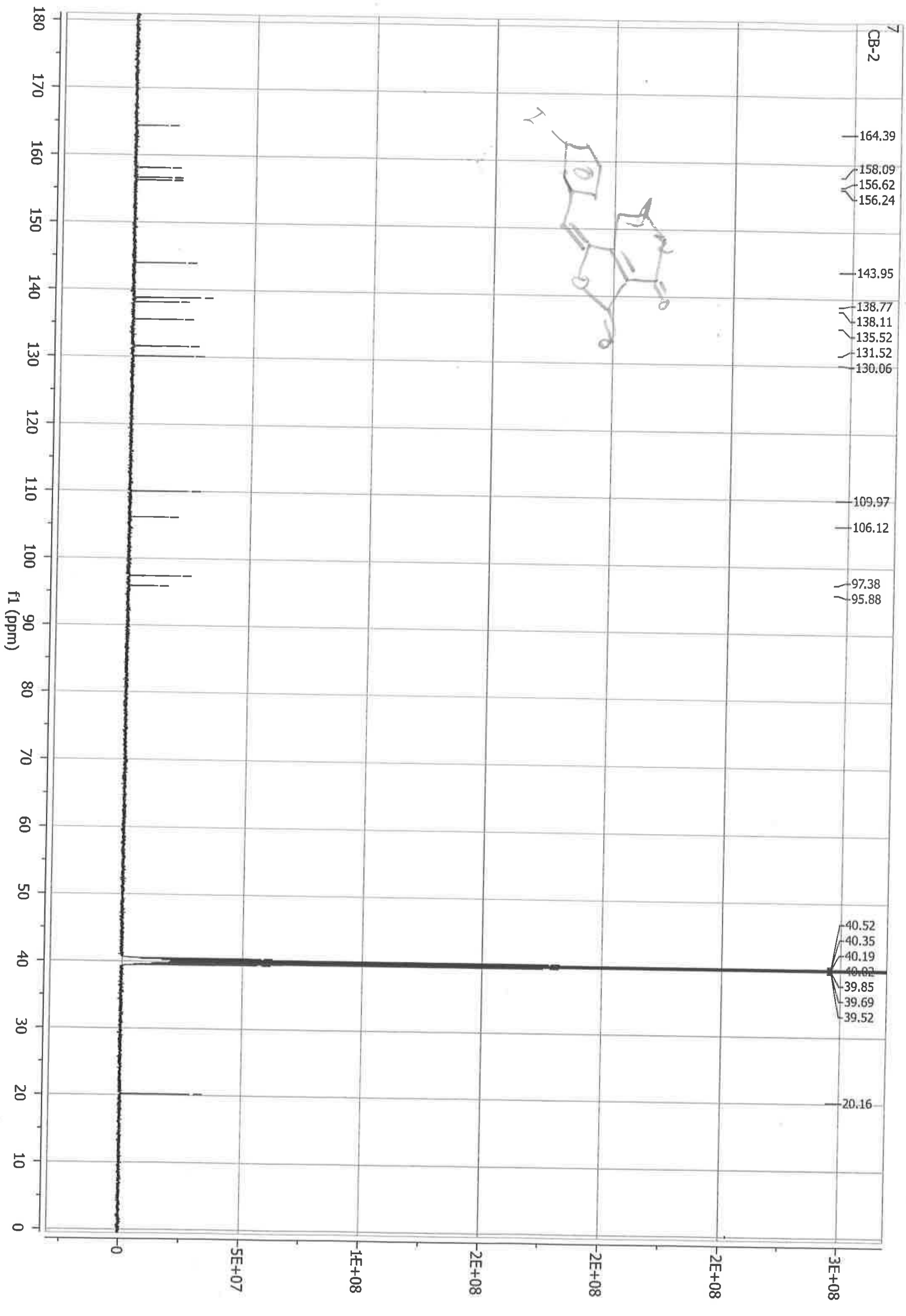

CB-2
164.39
158.09
156.62
156.24
143.95
138.77
138.11
135.52
131.52
130.06
109.97
106.12
97.38
95.88
40.52
40.35
40.19
40.02
39.85
39.69
39.52
20.16
f1 (ppm)
180
170
160
150
140
130
120
110
100
90
80
70
60
50
40
30
20
10
0
5E+07
1E+08
2E+08
2E+08
2E+08
3E+08

Compound 17

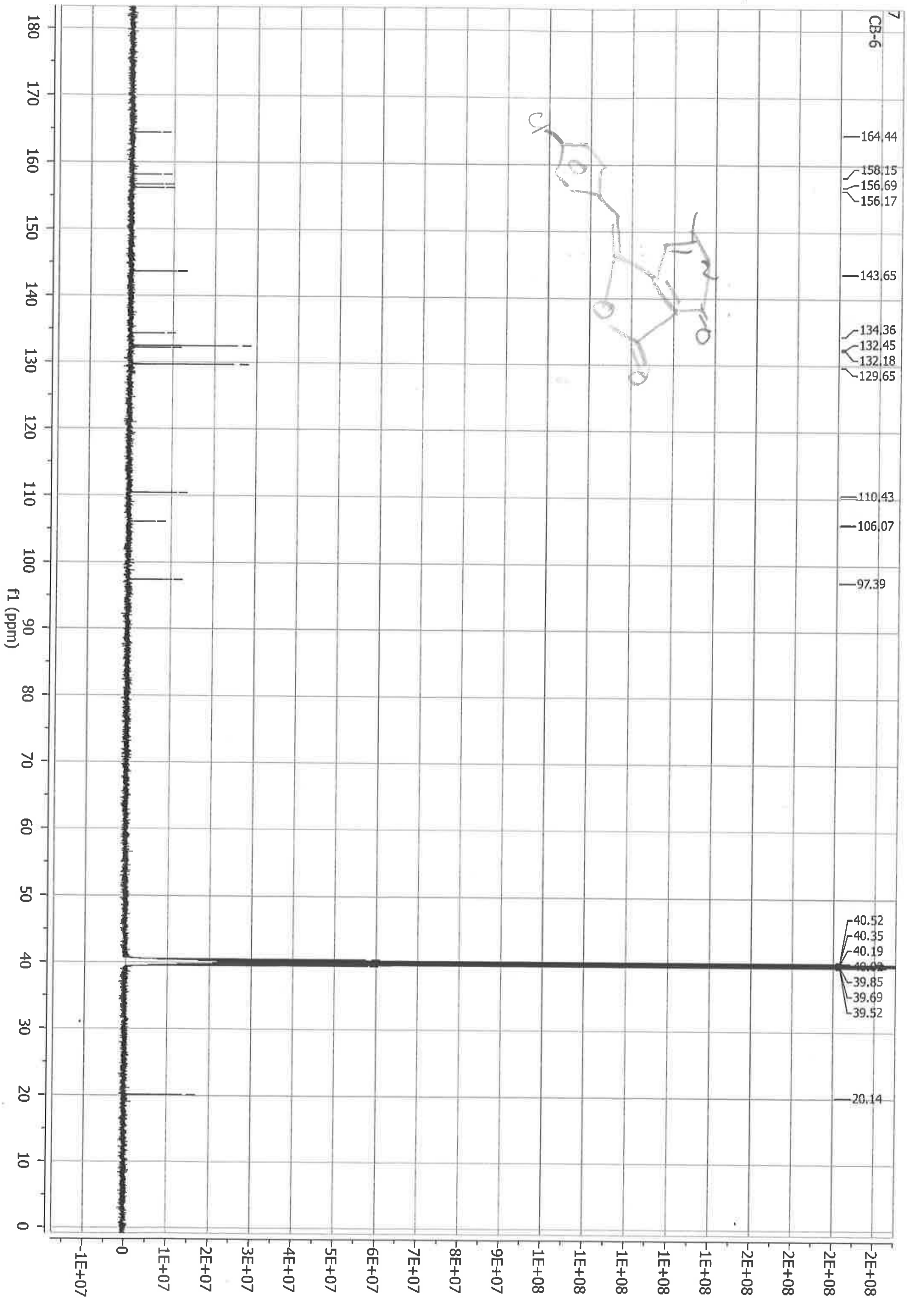

CB-6
164.44
158.15
156.69
156.17
143.65
134.36
132.45
132.18
129.65
110.43
106.07
97.39
40.52
40.35
40.19
39.85
39.69
39.52
20.14
f1 (ppm)
Cl

Compound 18

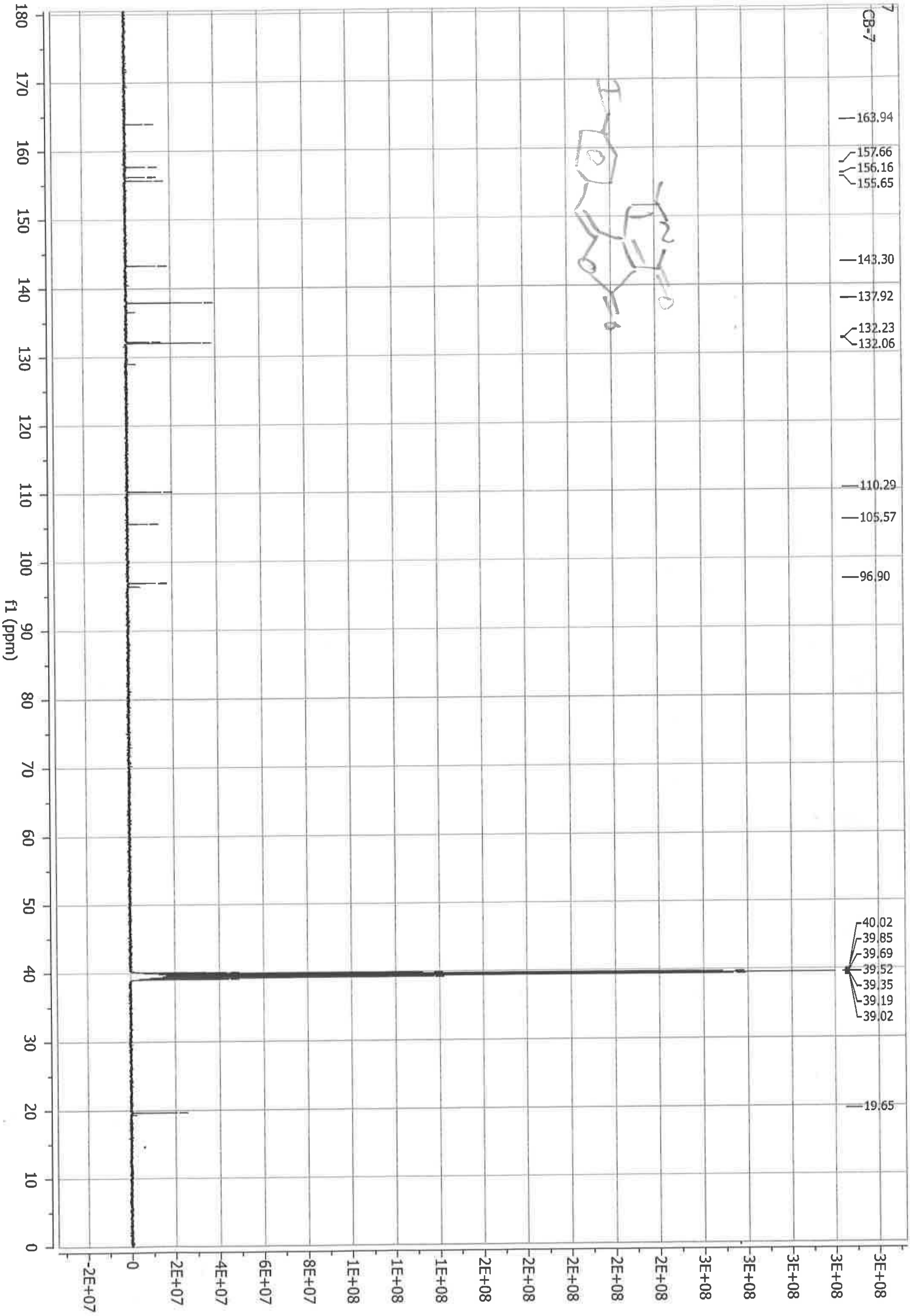
CB-7
163.94
157.66
156.16
155.65
143.30
137.92
132.23
132.06
110.29
105.57
96.90
40.02
39.85
39.69
39.52
39.35
39.19
39.02
19.65
f1 (ppm)

Compound 19

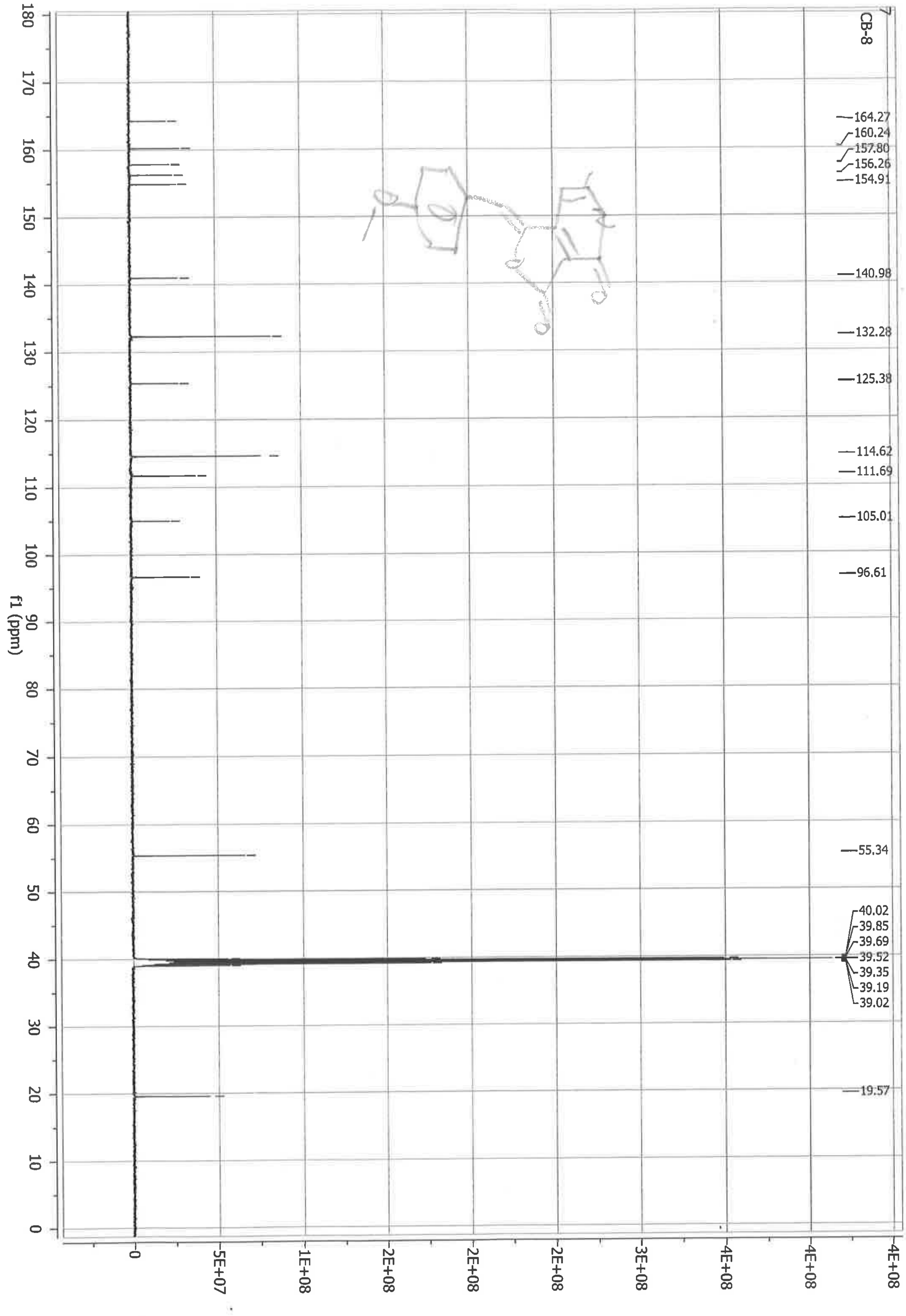

CB-8
164.27
160.24
157.80
156.26
154.91
140.98
132.28
125.38
114.62
111.69
105.01
96.61
55.34
40.02
39.85
39.69
39.52
39.35
39.19
39.02
19.57
f1 (ppm)
180
170
160
150
140
130
120
110
100
90
80
70
60
50
40
30
20
10
0
5E+07
1E+08
2E+08
3E+08
4E+08

Compound 20

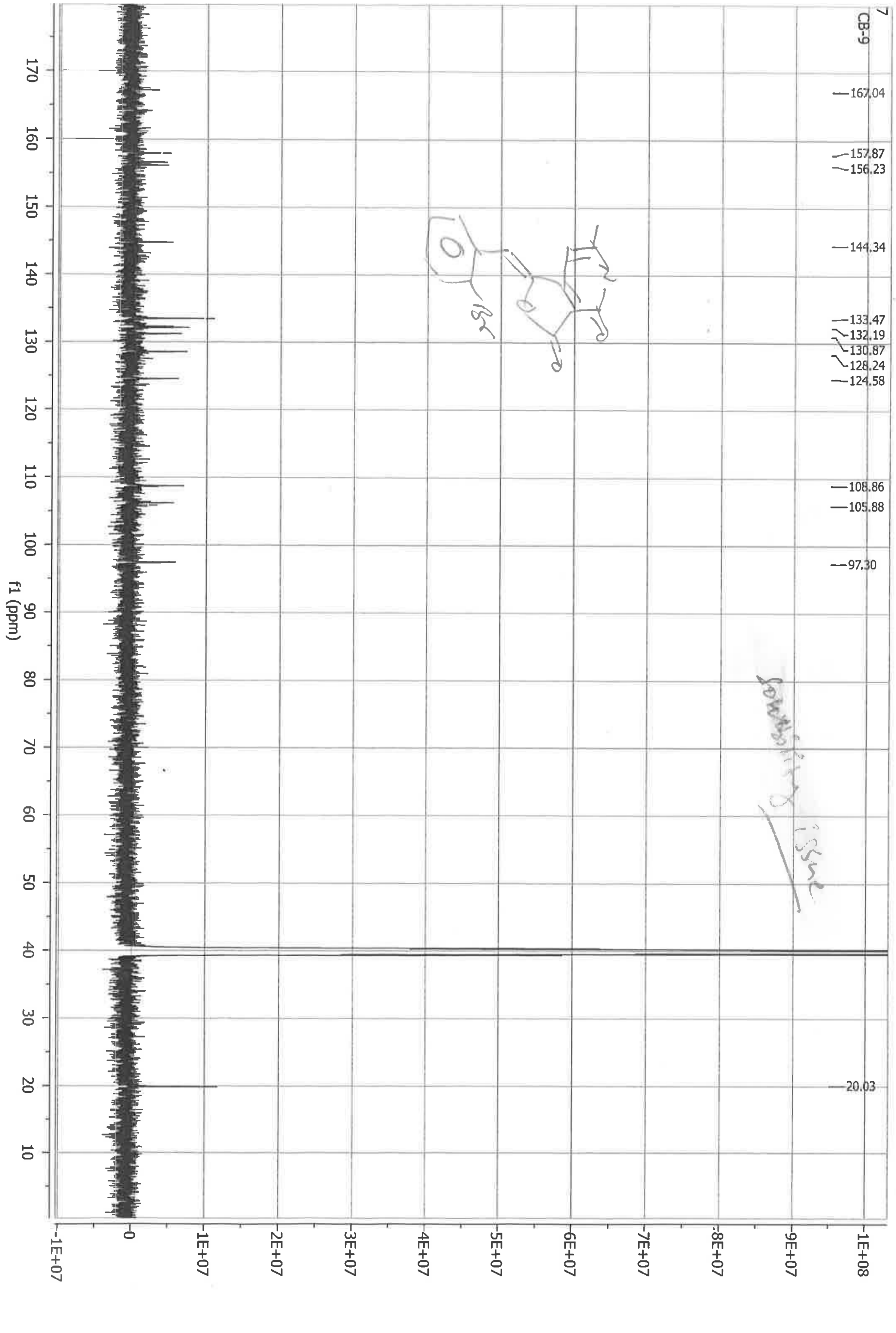

CB-9
167.04
157.87
156.23
144.34
133.47
132.19
130.87
128.24
124.58
108.86
105.88
97.30
20.03
f1 (ppm)

Compound 21

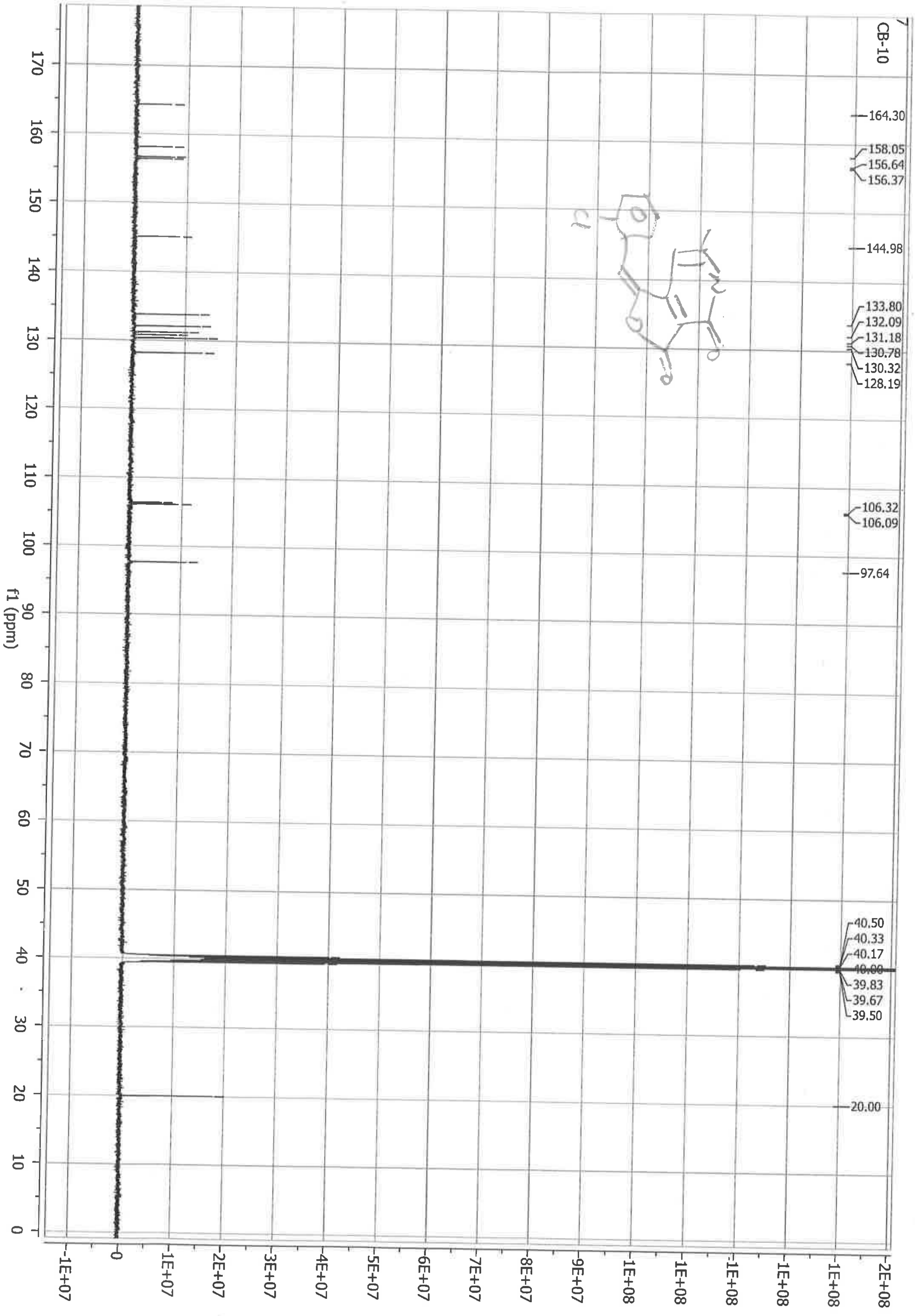
CB-10
164.30
158.05
156.64
156.37
144.98
133.80
132.09
131.18
130.78
130.32
128.19
106.32
106.09
97.64
40.50
40.33
40.17
40.00
39.83
39.67
39.50
20.00
f1 (ppm)
